# Supplementary material for: Specific targeting of cancer vaccines to antigen-presenting cells via an endogenous TLR2/6 ligand derived from cysteinyl-tRNA synthetase 1
Source: Mol Ther. 2024 Jul 25;32(10):3597–617. doi: 10.1016/j.ymthe.2024.07.014 (PMC11489552; doi:10.1016/j.ymthe.2024.07.014)
Supplement: Document S1. Figures S1–S18 and Tables S1–S4 [file mmc1.pdf]

YMTHE, Volume *32*

Supplemental Information

Specific targeting of cancer vaccines to antigen-presenting cells via an endogenous TLR2/6 ligand derived from cysteinyl-tRNA synthetase 1

Hyeong Yun Kim, Seongmin Cho, Sang Bum Kim, Ee Chan Song, Wonchul Jung, Yun Gyeong Shin, Ji Hun Suh, Jihye Choi, Ina Yoon, Uijoo Kim, Hamin Ban, Sunkyo Hwang, Jeongwon Mun, Joohee Park, Nayoung Kim, Youngjin Lee, Myung Hee Kim, and Sunghoon Kim

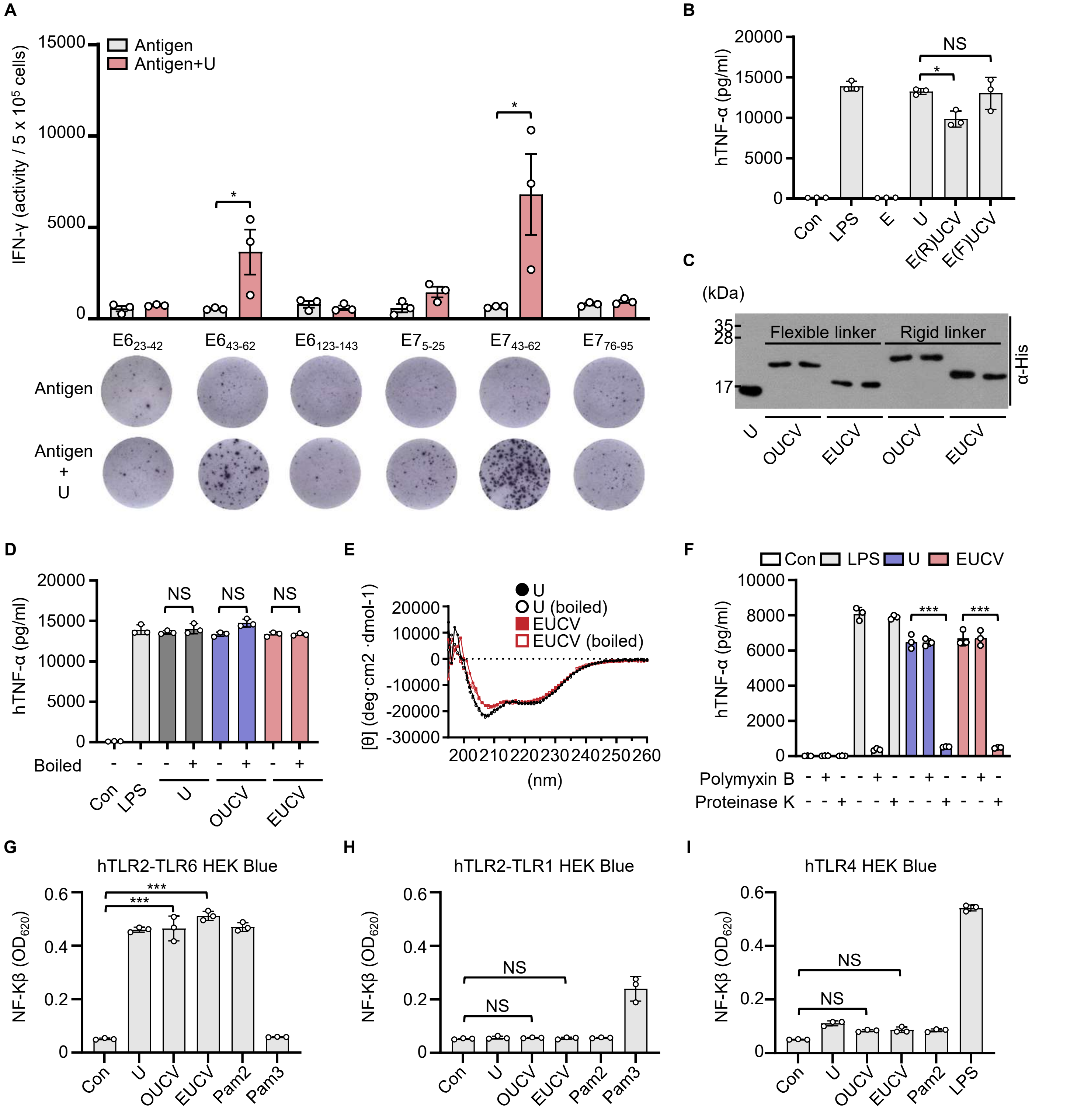

Fig S1. Optimization and physicochemical characterization of the UCV. (**A**) C57BL/6 mice were immunized twice at 2-week intervals with each of the HPV16 E6 or E 7 peptide antigens in combination with U (lower). Each peptide-specific IFN-γ-producing T-cell from the spleen was quantified using ELISpot after 1 week from final immu nization. (**B**) Production of TNF-α was measured by ELISA after the treatment of 100 nM of U, E(R)UCV, or E(F)UCV on PMA-differentiated THP-1 cells for 4 h. Some cel ls were treated with the E7 peptide and LPS as negative and positive controls, respectively. (**C**) Western blot analysis of U, EUCV, and OUCV (1 μg of total protein per lan e). Target bands were detected with an anti-His antibody. (**D**) PMA-differentiated THP-1 cells were treated with 100 nM of native and boiled U, EUCV, and OUCV for 4 h, TNF-α production was measured by ELISA. (**E**) Circular dichroism (CD) spectroscopy analysis of U and EUCV under native or boiled conditions at 195–260 nm. (**F**) PMA -differentiated THP-1 cells were treated with 100 nM U and EUCV for 4 h. U or EUCV were preincubated with proteinase K (50 μg/ml) for 1 h. Before treating U and EUC V, some cells were preincubated with polymyxin B (10 μg/ml) for 1 h, and LPS was used as a positive control. After 4 h of treatment, TNF-α production was measured by ELISA. (**G**–**I**) U, EUCV, and OUCV were treated with (**G**) hTLR2/6-, (**H**) hTLR2/1-, and (**I**) hTLR4- expressing HEK-Blue cells. SEAP activity was measured at OD_{620}. Pam 2CSK4, Pam3CSK4, and LPS were used as positive controls. Data are representative of three independent experiments, and the results are presented as mean ± SD. S tatistical significance was analyzed using Student's t-test (**P* < 0.05, ***P* < 0.01, ****P* < 0.001).

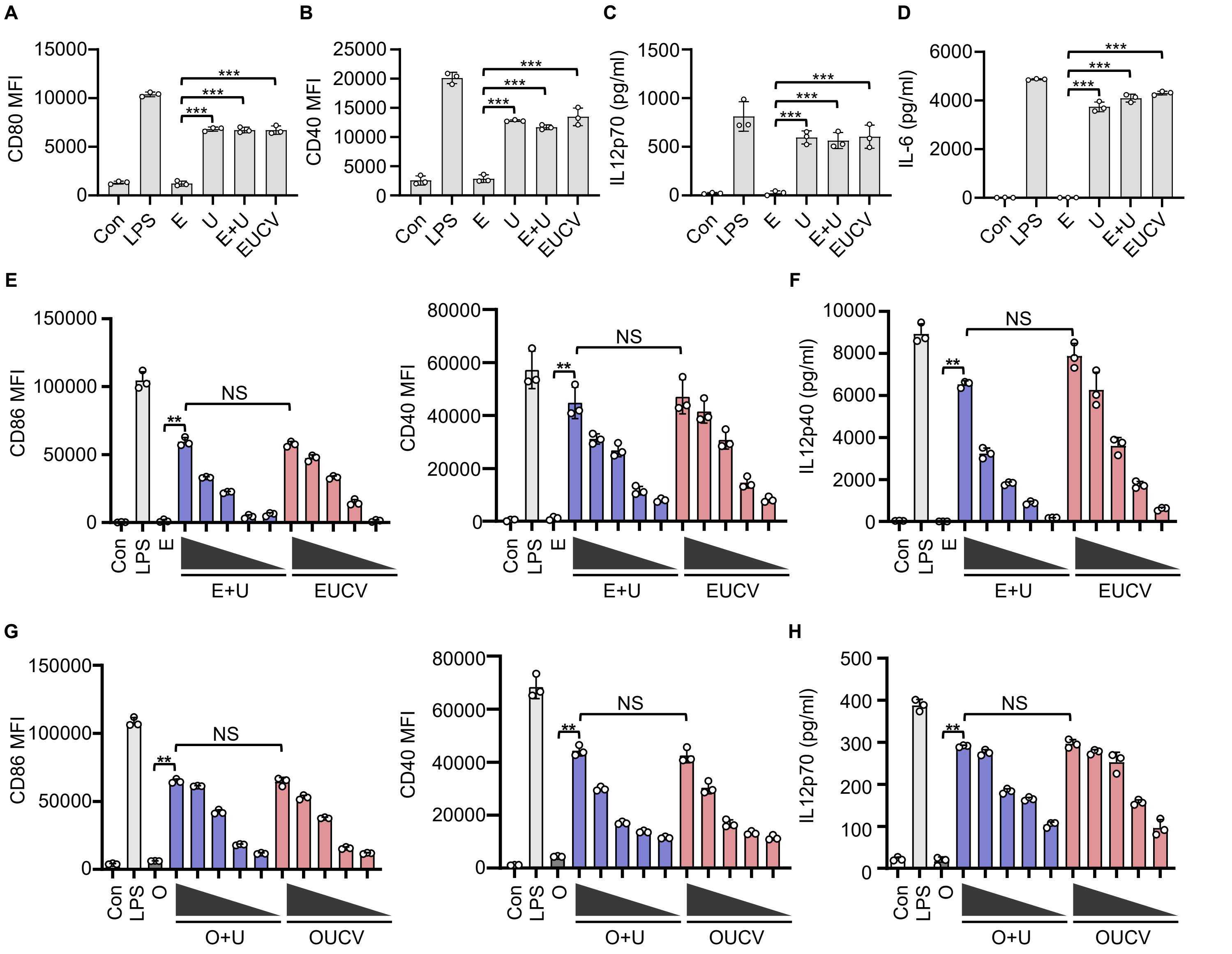

Fig S2. Comparison of the immune stimulation activities among UCV in APCs. (**A**–**D**) BMDCs were incubated with 100 nM E, U, E+U, and EUCV for 24 h. Costimulatory molecules were analyzed from the CD11c^{+} gating population, and (**A**) CD80 or (**B**) CD40 expression was evaluated by flow cytometry. The production of (**C**) IL-12p70 and (**D**) IL-6 was quantified by ELISA. E and LPS were used as negative and positive controls, respectively. (**E**, **F**) BMDCs were incubated with 100 nM E, E+U, and EUCV for 24 h. (**E**) Costimulatory molecules were analyzed from the CD11c^{+} gating population, and CD86 or CD40 expression was evaluated by flow cytometry. (**F**) IL-12p40 production was quantified by ELISA. E and LPS were used as negative and positive controls, respectively. (**G**, **H**) BMDCs were incubated with 100 nM O, O+U, and OUCV for 24 h. (**G**) Costimulatory molecules were analyzed from the CD11c^{+} gating population, and CD86 and CD40 expression was evaluated by flow cytometry. (**H**) IL-12p70 production was quantified by ELISA. O and LPS were used as negative and positive controls, respectively. Data are representative of three independent experiments, and the results are presented as the mean ± SD. Statistical significance was analyzed using Student's t-test (***$P < 0.001$). NS, not significant.

A

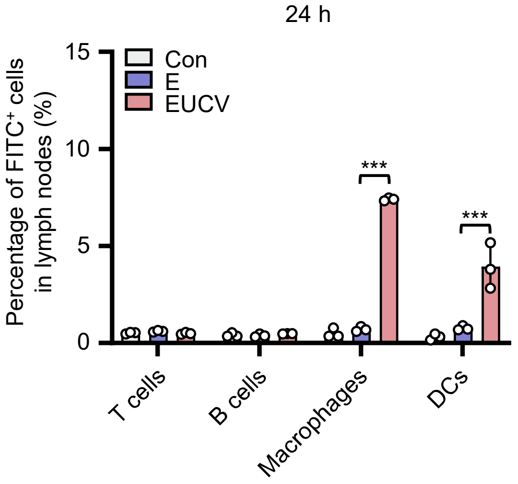

Fig S3. Comparison of antigen uptake among different cell types in lymph nodes. (**A**) C57BL/6 mice were subcutaneously injected with 5 nmol of FAM-labeled E or EUCV. Immune cells were harvested from excised inguinal lymph nodes at 24 h after injection, and the 5-FAM signal in various cell types was assessed by flow cytometry (n = 3 per group). Data are representative of three independent experiments, and the results are presented as the mean ± SEM. Statistical significance was analyzed usi ng two-way ANOVA (*P < 0.05, **P < 0.01, ***P < 0.001).

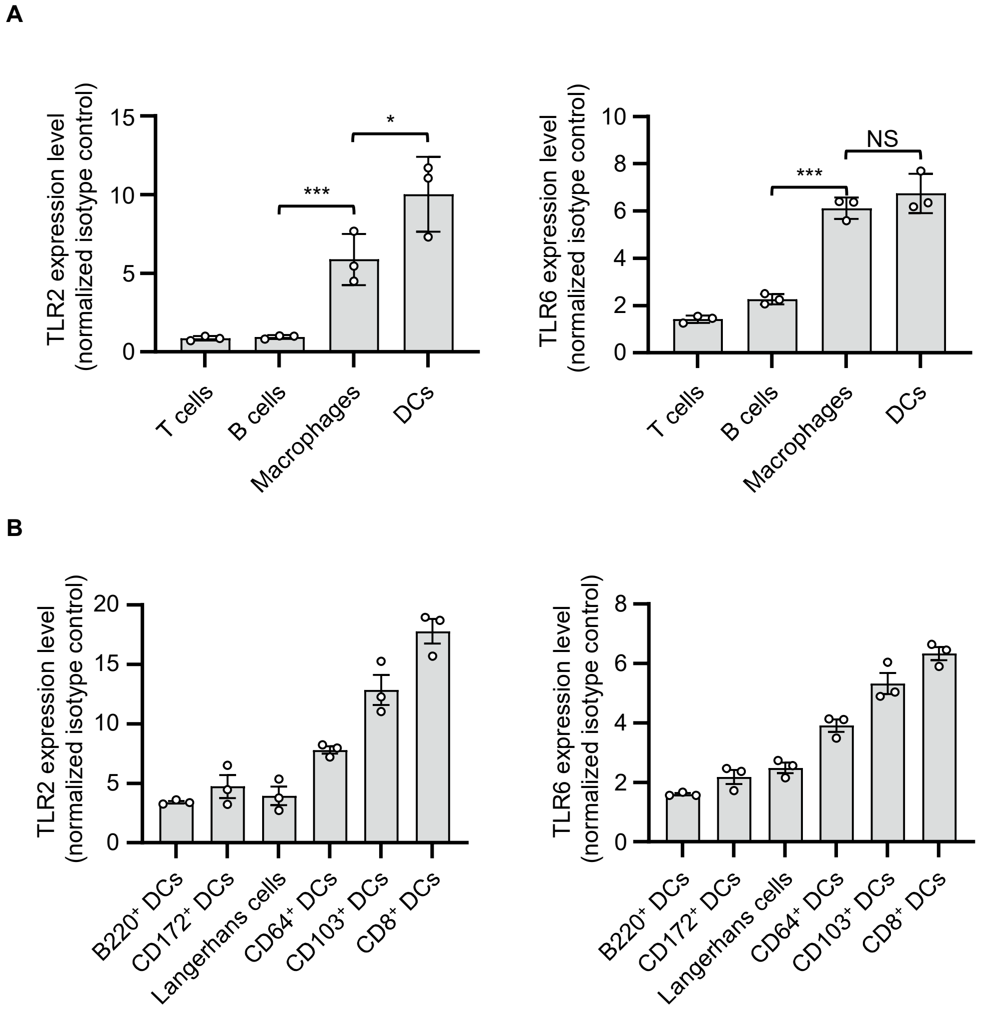

Fig S4. Comparison of TLR2 and TLR6 expression levels by DC subtype. Immune cells were isolated from lymph nodes extracted from naïve mice. TLR2 and TLR6 expression for each isolated (**A**) Immune cells and (**B**) DC subtype was measured using flow cytometry, and then normalized with isotype controls. Data are representative of three independent experiments, and the results are presented as the mean ± SD. Statistical significance was analyzed using Student's t-test (*P < 0.05, **P < 0.01, ***P < 0.001).

A

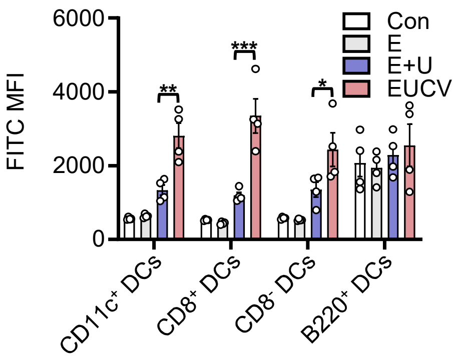

Fig S5. Evaluation of antigen uptake by DC subtypes. (**A**) C57/BL6 mice were subcutaneously injected with 5 nmol of FAM-labeled E, E+U, and EUCV at the right bac k. Antigen uptake was evaluated from DC subtypes residing in the inguinal LN 24 h after subcutaneous injection (*n* = 4 per group). Data are representative of three independent experiments, and the results are presented as the mean ± SD. Statistical significance was analyzed using Student's t-test (*P < 0.05, **P < 0.01, ***P < 0.001).

A

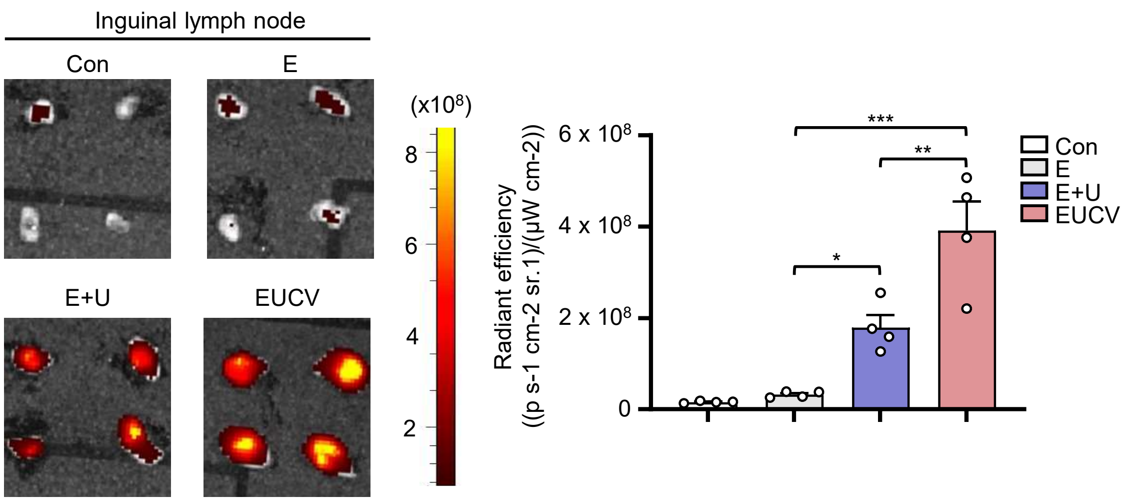

Fig S6. Analysis of vaccine localization patterns in inguinal lymph nodes among different E7 vaccine treatments. (**A**) C57BL/6 mice were subcutaneously injecte d with 5 nmol FAM-labeled E, E+U, and EUCV. After 24 h, lymph nodes isolated from each mouse were imaged, and the signal intensities were quantified using IVIS (n = 4 per group). Data are representative of three independent experiments, and the results are presented as mean ± SEM. Statistical significance was analyzed using Stude nt's t-test (*P < 0.05, **P < 0.01, ***P < 0.001).

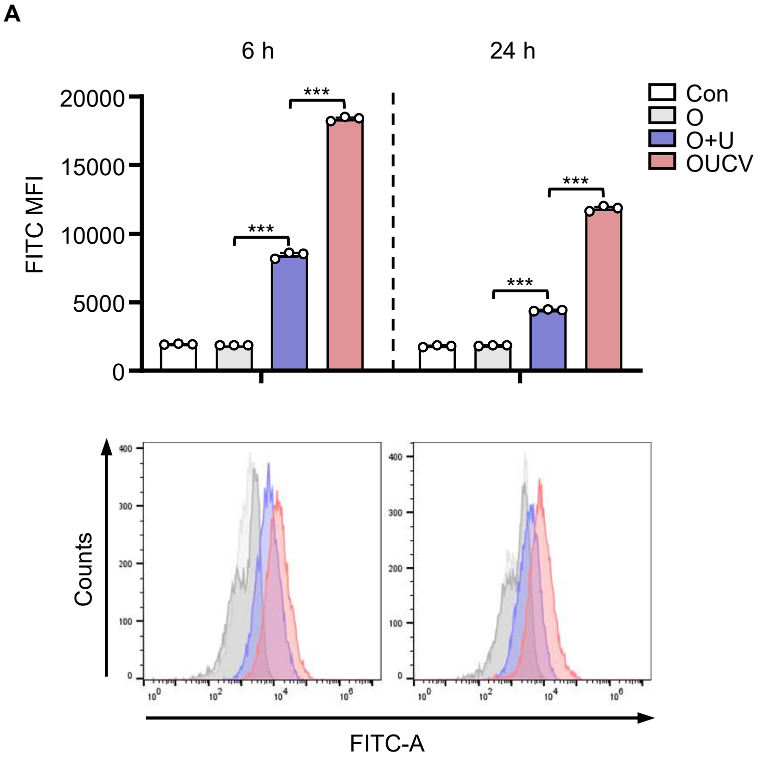

Fig S7. Comparison of OVA uptake among different OVA-based vaccine treatments. (**A**) To investigate the time-dependent uptake of antigens, isolated BMDCs (3× 10^5) were seeded in 24-well plates and incubated with FAM-labeled O, O+U, and OUCV at 37 °C and 5% CO_2 for 6 or 24 h. BMDCs were harvested and stained with 1 μ g/ml APC-conjugated anti-CD11c antibody for 30 min at 4 °C, and antigen uptake was analyzed by flow cytometry. Data are representative of three independent experime nts, and the results are presented as the mean ± SD. Statistical significance was analyzed using Student's t-test (*$P < 0.05$, **$P < 0.01$, ***$P < 0.001$).

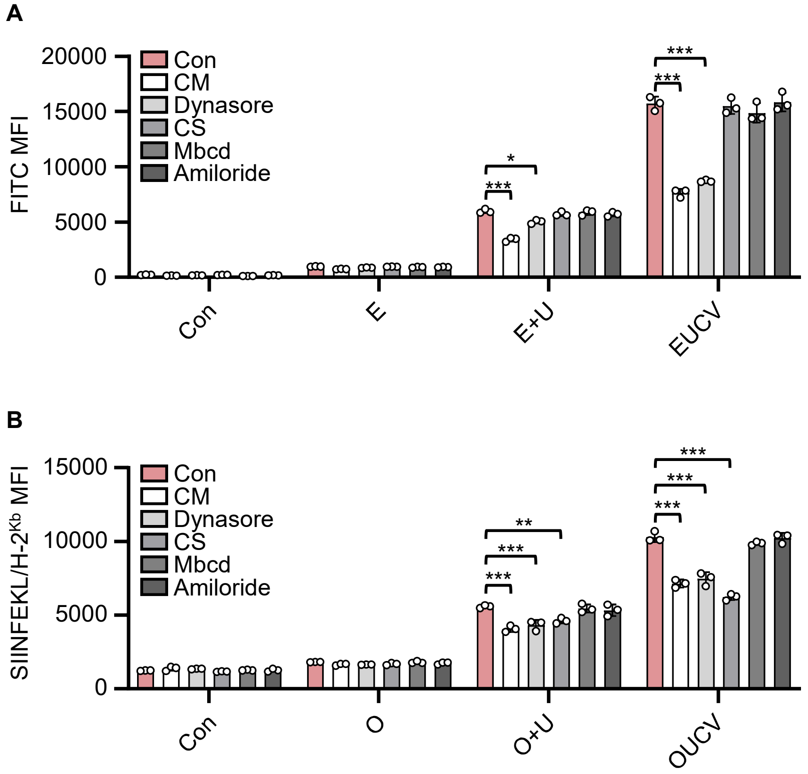

Fig S8. Analysis of the Impact of Inhibitors on Internalization or Antigen Presentation. (**A**) BMDCs isolated from naïve mice were treated with CM (20 μM), Dynaso re (10 μM), CS (1 μM), Mbcd (1 μM) and Amiloride (1 mM) for 30 minutes. To assess antigen uptake, FAM-labeled E, E+U, and EUCV were each treated at 100 nM, and after 24 hours, the FITC+ signal was analyzed by flow cytometry to evaluate the uptake. (**B**) Antigen presentation was evaluated by treating BMDCs with O, O+U, and OUCV after applying each inhibitor, and then measuring the SIINFEKL/H-2Kb signal via flow cytometry 24 hours later. Data are representative of three independent experiments, and the results are presented as the mean ± SD. Statistical significance was analyzed using Student's t-test (*P < 0.05, **P < 0.01, ***P < 0.001).

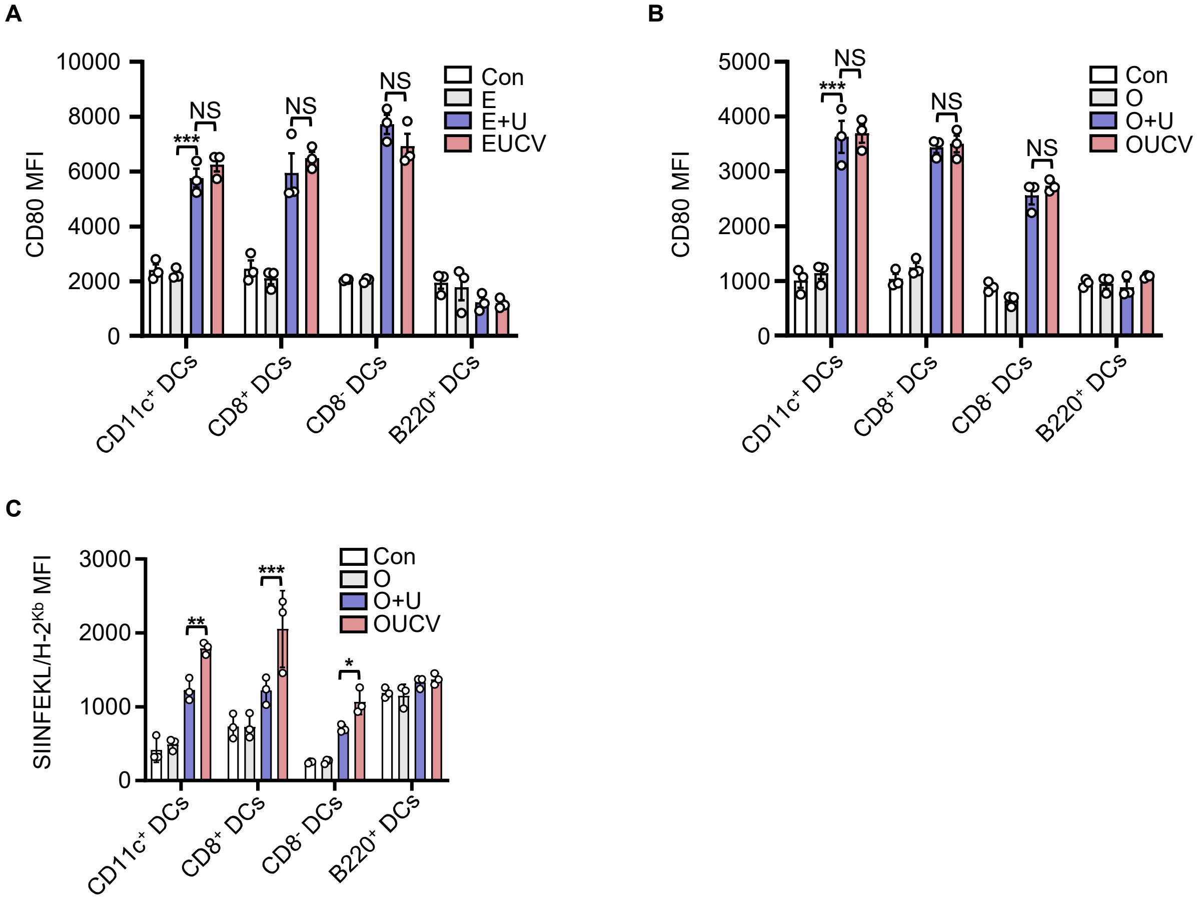

Fig S9. Comparison of costimulatory molecule expression and antigen presentation on DCs injected with different E7- or OVA-based vaccines. (**A**, **B**) Expressi on of the costimulatory molecule CD80 was determined in DC subtypes residing in inguinal lymph nodes 24 h after subcutaneous injection with E7- (**A**) and OVA-based (**B**) vaccines (*n* = 3 per group). (**C**) Flow cytometric analysis of SIINFEKL/H-2Kb expression on LN-residing DC subtypes was performed after subcutaneous injection with 5 nmol of O, O+U, or OUCV, respectively (*n* = 3 per group). Data are representative of three independent experiments, and the results are presented as the mean ± SD. Statistical significance was analyzed using Student's t-test (*P < 0.05, **P < 0.01, ***P < 0.001).

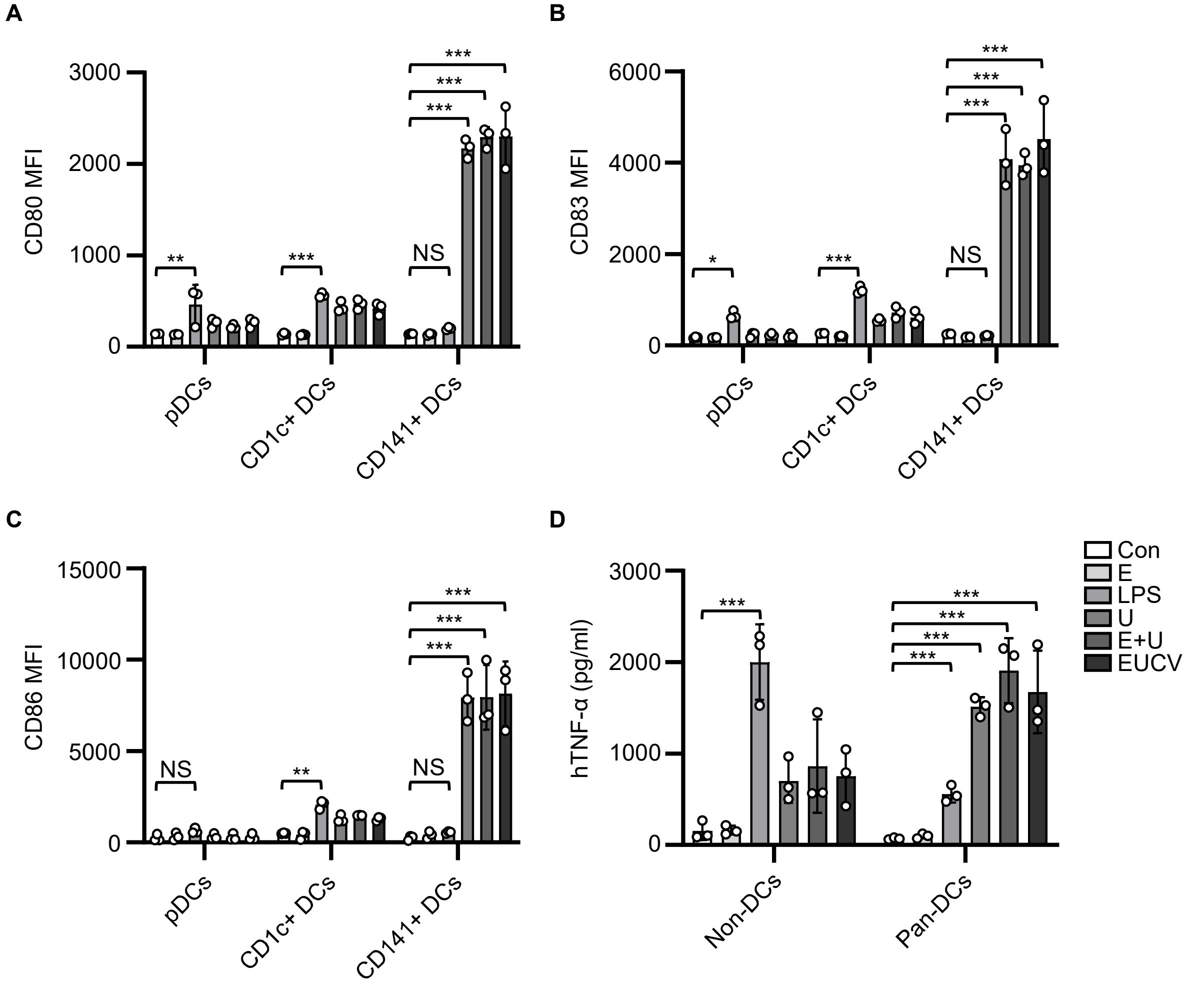

Fig S10. Comparison of the immune stimulation activities of the UCV in Human PBMC isolated DCs. The expression of costimulatory molecules (**A**) CD80, (**B**) CD 83, and (**C**) CD86 was measured using flow cytometry 24 h after treating Pan-DCs isolated from human PBMCs with E, LPS, Poly(I:C), U, E+U, and EUCV, respectively (*n* = 3). (**D**) The inflammatory cytokine hTNF-α levels were quantified using ELISA in Pan-DCs and Non-DCs isolated from human PBMCs, treated with E, LPS, Poly(I:C), U, E+U, and EUCV for 24 hours (*n* = 3). Data are representative of three independent experiments, and the results are presented as the mean ± SD. Statistical significance was analyzed using Student's t-test (*P < 0.05, **P < 0.01, ***P < 0.001).

A

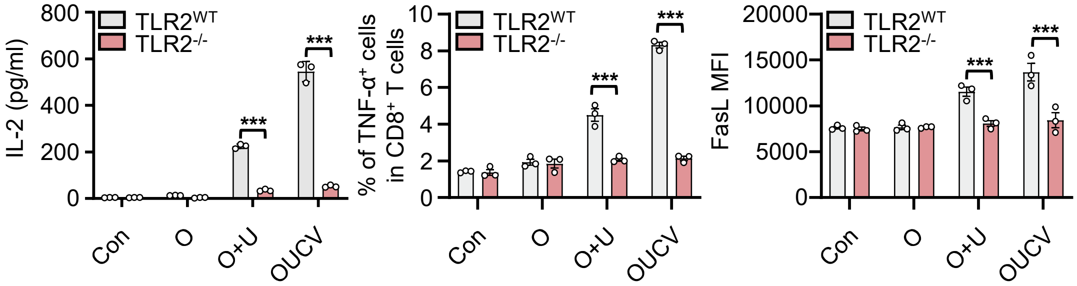

Fig S11. Comparison of CD8^{+} T-cell stimulation among different OVA-based vaccine treatments. (**A**) BMDCs from TLR2 WT and TLR2$^{-/-}$ mice were incubated for 24 h with O, O+U, or OUCV and cocultured overnight in the presence of B3Z CD8^{+} T cells. T-cell activation was quantified by IL-2 expression using ELISA, and TNF-α^{+} CD8^{+} T cells population or FasL expression using flow cytometry. Data are representative of three independent experiments, and the results are presented as the mean ± SEM. Statistical significance was analyzed using Student's t-test (****P* < 0.001). NS, not significant.

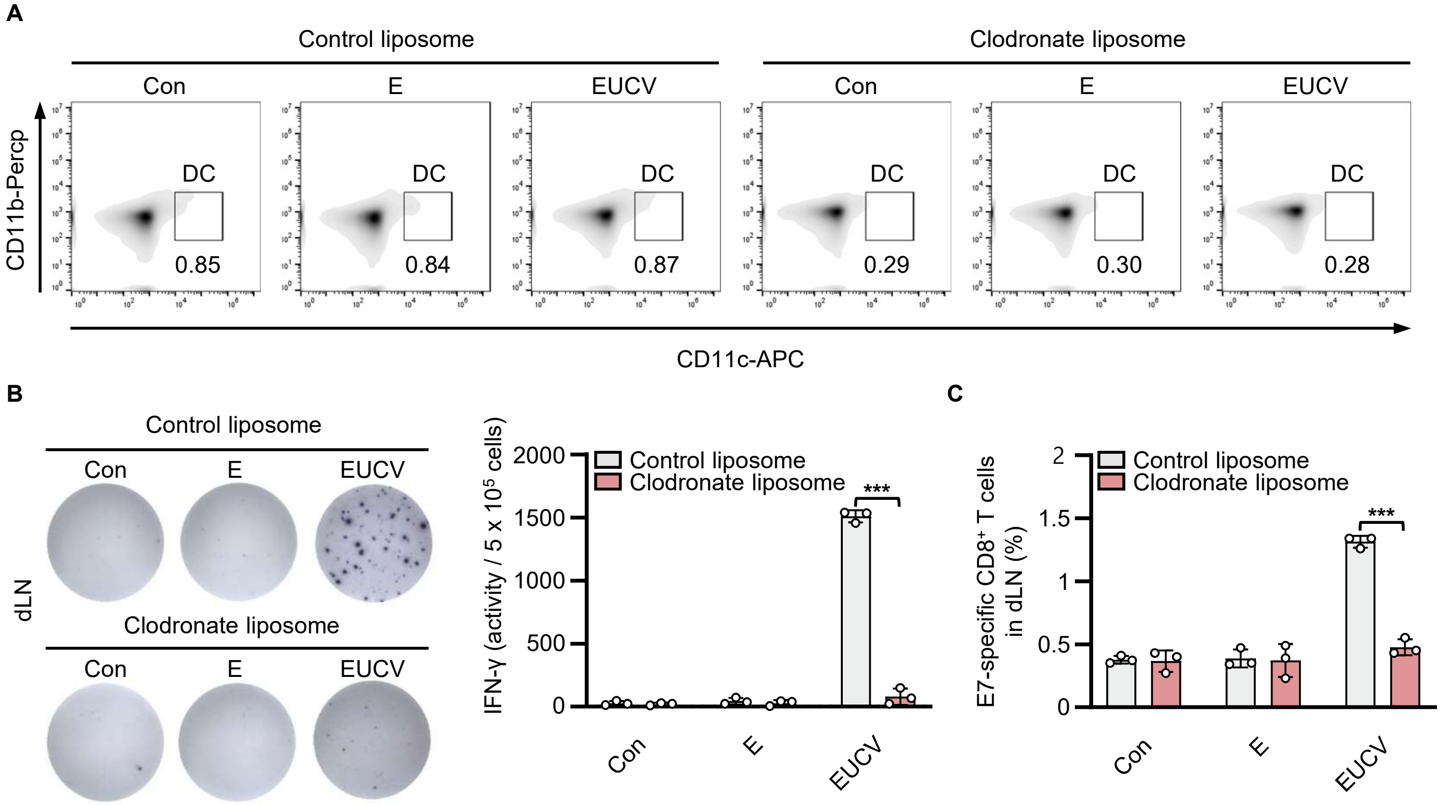

Fig S12. Investigation of DC-dependent Immune Response of EUCV using DC depletion. (**A-C**) C57/BL6 mice were administered 200 μL each of control liposomes and clodronate liposomes via intraperitoneal injection once per week. One day after liposome injection, E and EUCV were each administered at 5 nmol. (**A**) A week after t he last injection, mouse lymph nodes were isolated to confirm DC depletion by flow cytometry (n=3 per group). (**B**) The isolated immune cells (5×10^5 cells) from lymph node were ex vivo stimulated with the E7 epitope (2 μg/ml) for 48 h and analyzed by an ELISpot reader (n = 3 per group). (**C**) Percentages of E7-specific CD8+ T cells in the lymph node were measured using E7 tetramers by flow cytometry (n = 3 per group). Data are representative of three independent experiments, and the results are pr esented as the mean ± SEM. Statistical significance was analyzed using Student's t-test (****P* < 0.001). NS, not significant.

A

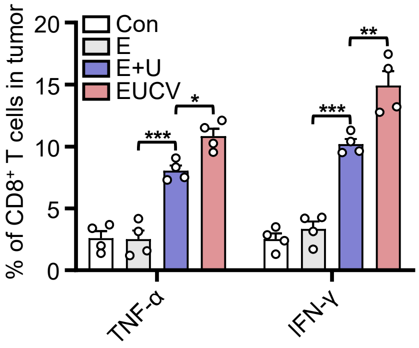

Fig S13. Comparison of CD8+ T-cell stimulation among different E7-based vaccine treatments. (**A**) C57BL/6 mice were subcutaneously inoculated with 1×10^5 TC-1 cells. On day 6 and 13, mice were subcutaneously injected with 5 nmol of E, E+U, and EUCV, and tumors were excised on day 20, and TILs were isolated from tumor tiss ues. The frequencies of TNF-α+ or IFN-γ+ CD8+ T cells were assessed by flow cytometry. Data are representative of three independent experiments, and the results are presented as the mean ± SEM. Statistical significance was analyzed using Student's t-test (*P < 0.05, **P < 0.01, ***P < 0.001). TNF-α, tumor necrosis factor-alpha; IFN-γ, interferon-gamma;

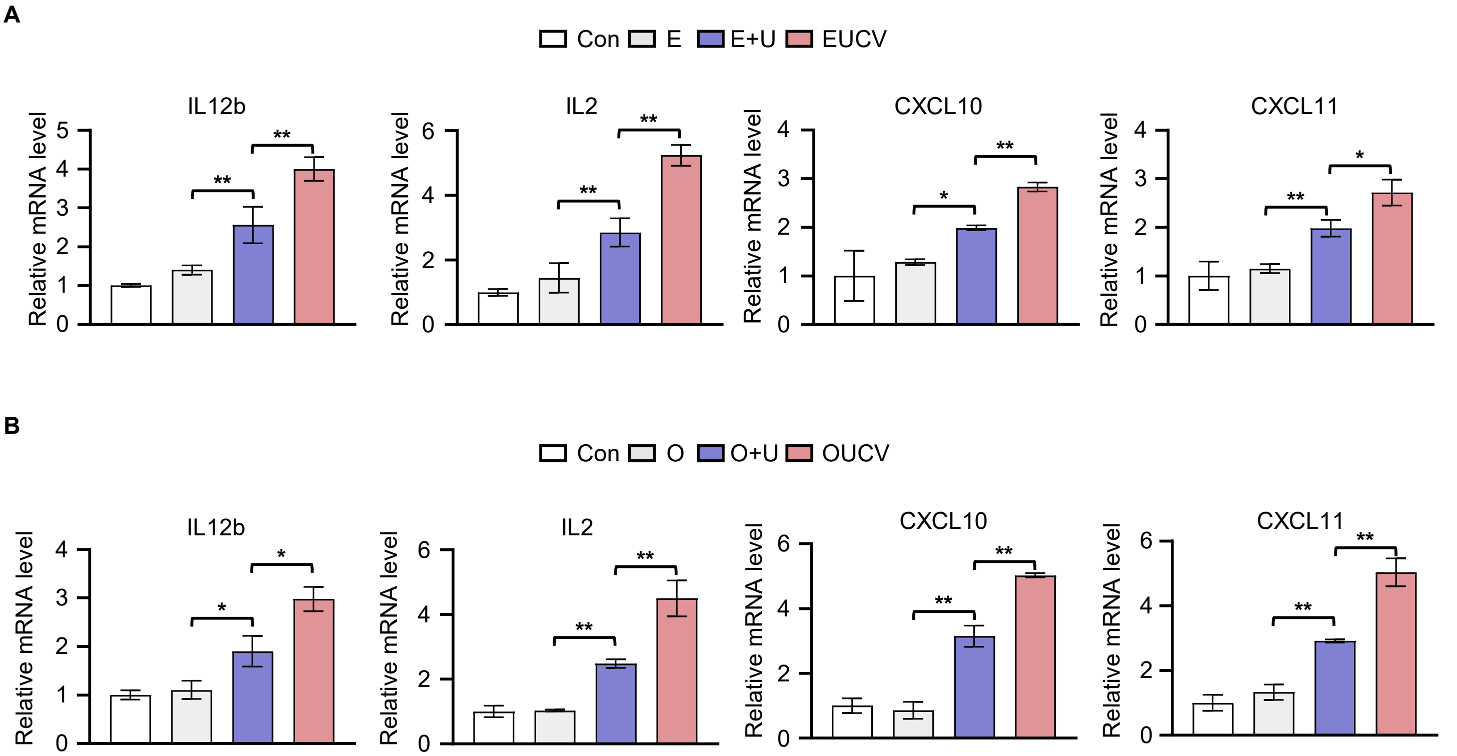

Fig S14. Analysis of CD8⁺ T Cell activation-related gene expression using tumor tissue. (**A-B**) The assessment of gene expression was performed via qPCR using resected TC-1 tumor tissue (**A**) and E.G7-OVA tumor tissue (**B**). Data are representative of three independent experiments, and the results are presented as the mean ± SEM. Statistical significance was analyzed using Student's t-test (*P < 0.05, **P < 0.01, ***P < 0.001).

A

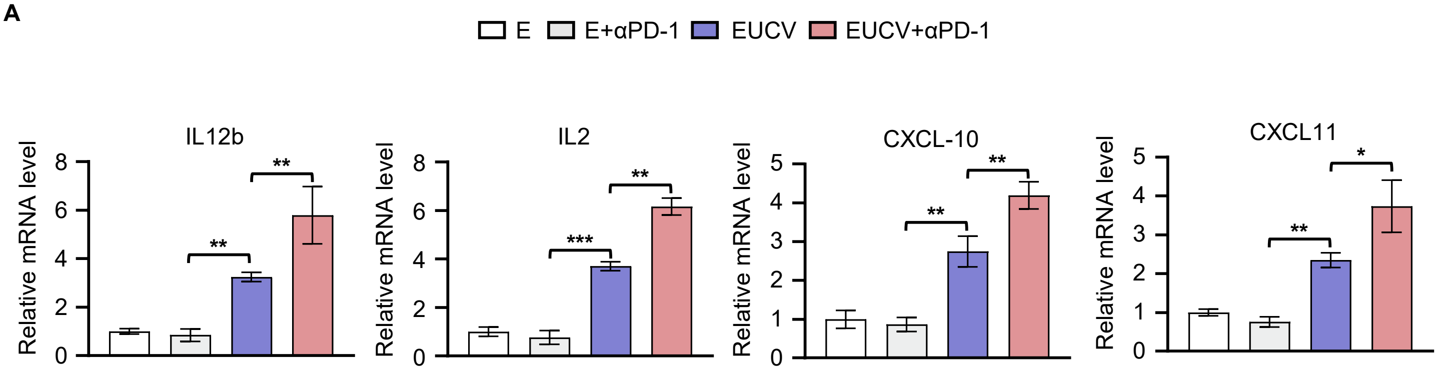

Fig S15. Investigation of CD8⁺ T cell-related gene expression following combined administration of EUCV and PD-1 in a TC-1 tumor model. (**A**) The analysis of gene expression was performed via qPCR using resected tumor tissue. Data are representative of three independent experiments, and the results are presented as the mean ± SEM. Statistical significance was analyzed using Student's t-test (*P < 0.05, **P < 0.01, ***P < 0.001).

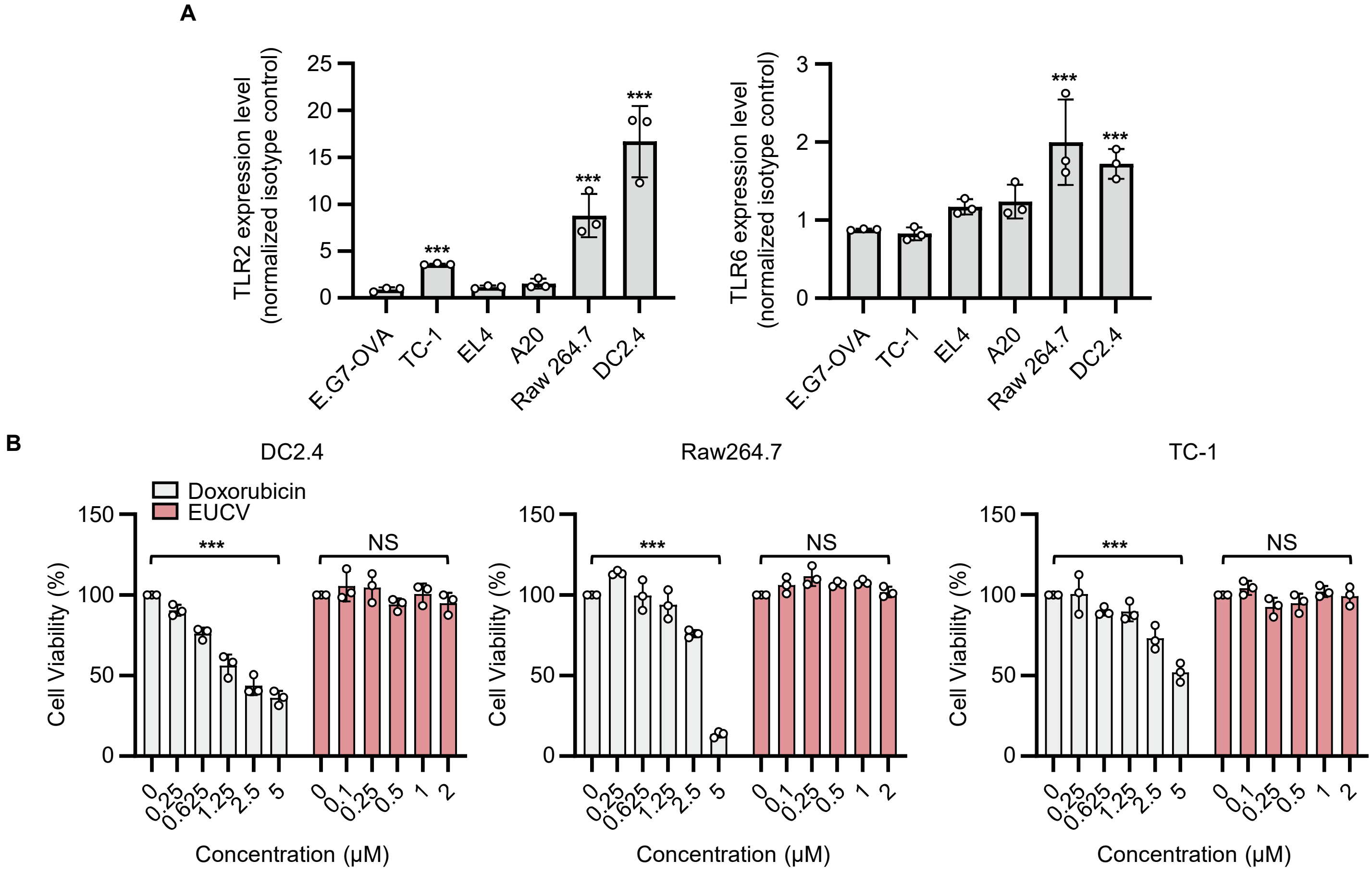

Fig S16. Detection of TLR2 and TLR6 expression levels and verification of EUCV cytotoxicity in cell line. (**A**) TLR2 and TLR6 expression levels were measured by flow cytometry in mouse cancer cell lines E.G7-OVA (lymphoma) and TC-1 (lung cancers), along with immune cell lines EL4 (T cells), A20 (B cells), Raw 264.7 (Macrophages), and DC2.4 (Dendritic cells). The measured signals were normalized using each isotype control. (**B**) DC2.4, Raw264.7, TC-1 cells were seeded onto 96-w ell plates for 24 h before treating with different concentrations of EUCV and Doxorubicin. Cell viability was measured using the Cell Counting Kit-8 (CCK-8) 18 hours after treatment. Data are representative of three independent experiments, and the results are presented as the mean ± SEM. Statistical significance was analyzed using Stud ent's t-test (*P < 0.05, **P < 0.01, ***P < 0.001).

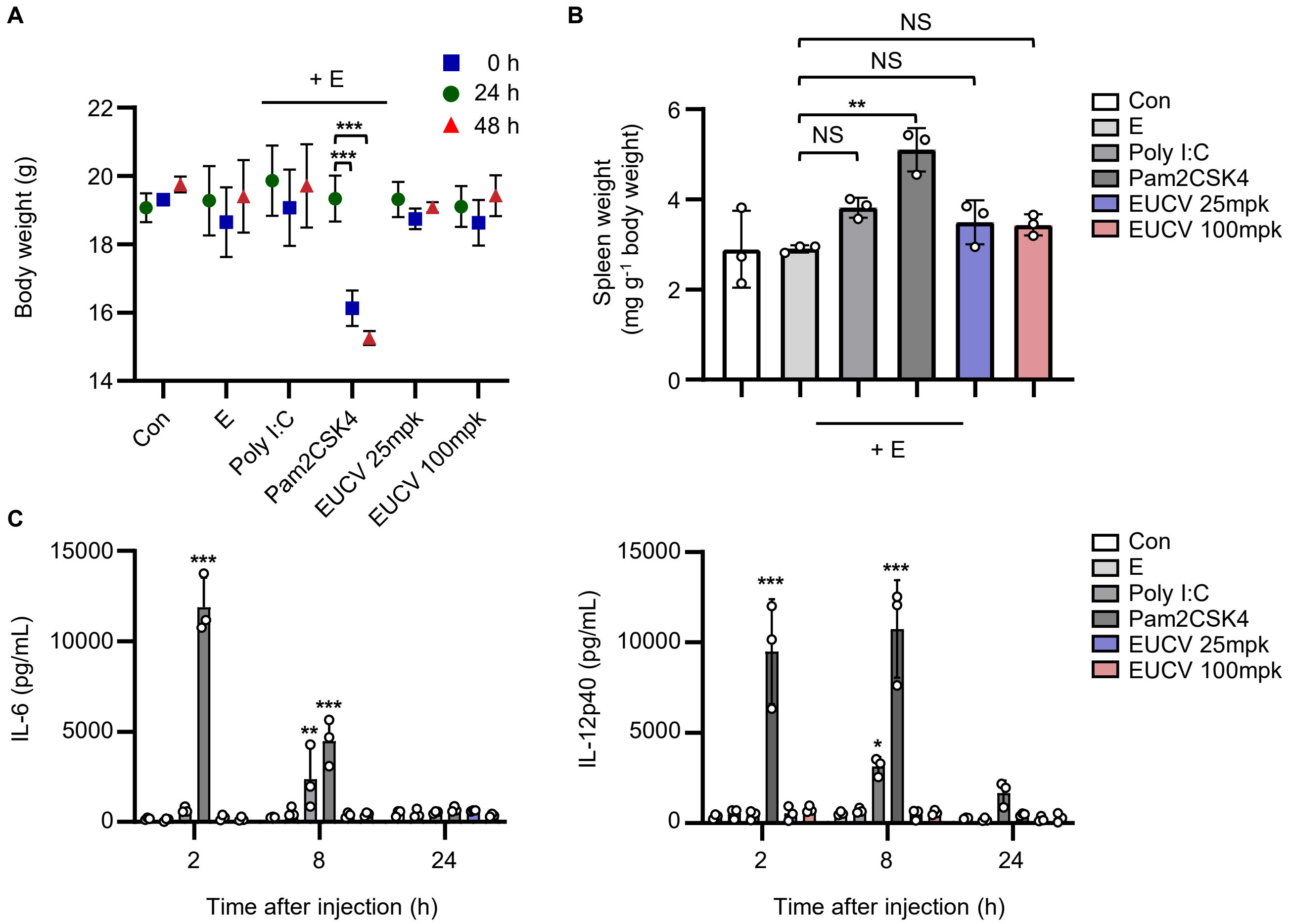

Fig S17. Evaluation of In vivo safety of EUCV. (**A-C**) C57BL/6 mice were administered E, Poly I:C, Pam2CSK4, or EUCV (25 or 100 mpk). (**A**) Body weight was measured every 24 hours, and 48 hours after administration, (**B**) the mice were euthanized to measure spleen weight. (**C**) Proinflammatory cytokines IL-6 and IL-12p40 were measured using ELISA with serum extracted from the mice. Data are representative of three independent experiments, and the results are presented as the mean ± SEM. Statistical significance was analyzed using Student's t-test (*P < 0.05, **P < 0.01, ***P < 0.001).

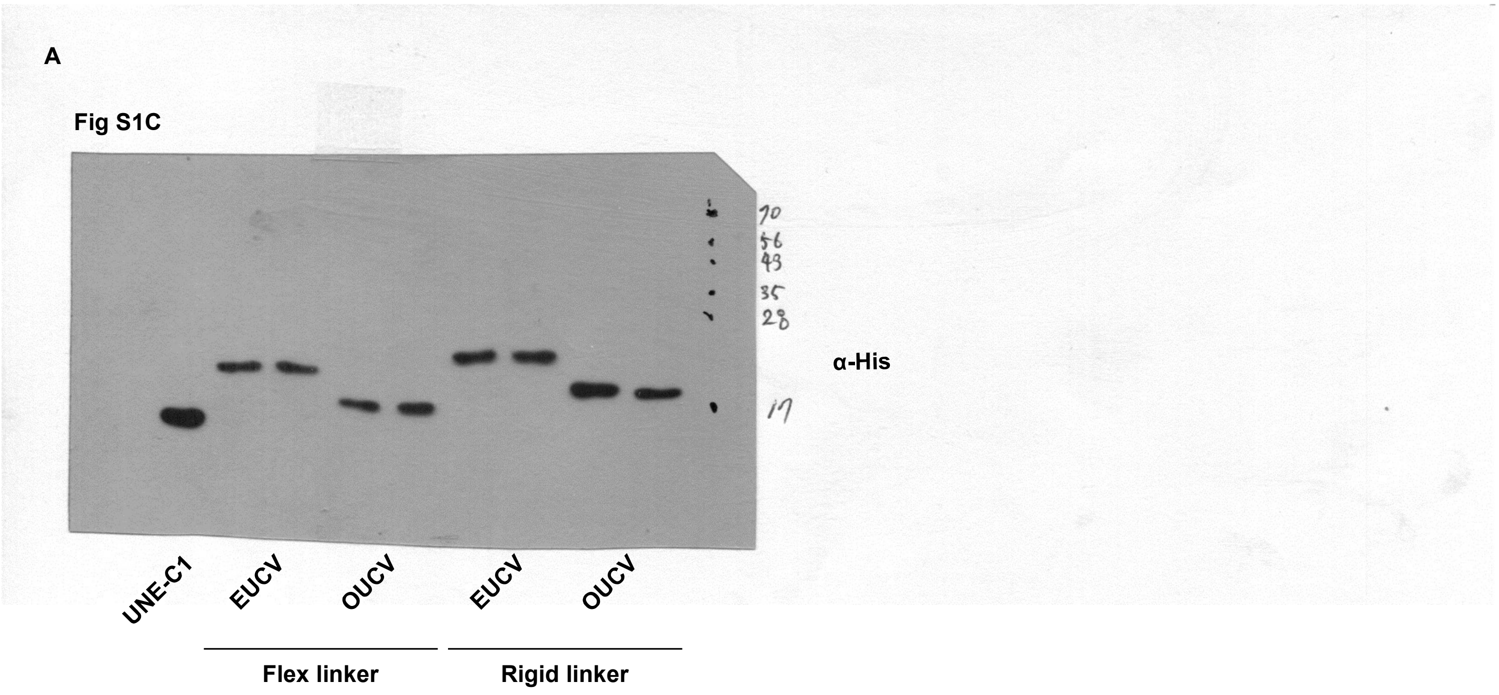

Fig S18. Raw data for Western blots included in the figures. (**A**) Uncropped image of western blot displayed in Fig S1C.

Table S1. The amino acid sequences of SLP antigens and the UCVs used in this study.

Abbreviation	Sequence
E (HPV16 E7 oncoprotein, 43~62 aa)	GQAEPDRAHYNIVTFCCKCD
O (Ovalbumin, 247~264 aa + AAAAK)	DEVSGLEQLESIINFEKLAAAAAK
EUCV (HPV 16 E7 43-62 aa conjugated UNE-C1)	GQAEPDRAHYNIVTFCCKCDGGGGSGGGGSGGGGSARQNHLFEQYREKRPEAAQLLEDVQAALKPFSVKLNETTDPDKKQMLERIQHAVQLATEPLEKAVQSRLTGEEVNSCVEVLLEEAKDLLSDWLDSTLGCDVTDNSIFSKLPKFWEGDFHRDME
OUCV (Ovalbumin 247-264 aa +AAAAK conjugated UNE-C1)	DEVSGLEQLESIINFEKLAAAAAKGGGGSGGGGSGGGGSARQNHLFEQYREKRPEAAQLLEDVQAALKPFSVKLNETTDPDKKQMLERIQHAVQLATEPLEKAVQSRLTGEEVNSCVEVLLEEAKDLLSDWLDSTLGCDVTDNSIFSKLPKFWEGDFHRDME

Table S2. The DNA sequences of the UCVs used in this study.

Plasmid	Sequence
pET28a-E(F)UCV → The sequence of 6xHis-E7$_{43-62}$-(GGGGS)$_3$-UNE-C1-6xHis as shown in left was inserted into pET28a back bone using NdeI and XhoI. 20729.93	TATACCATGGGCAGCAGCCATCATCATCATCATCACAGCAGCGGCCTGGTGCCGCGCGGCAGC<br>CATATG<br>GGTCAAGCTGAACCAGATCGTGCTCACTATAACATTGTTACTTTCTGTTGTAAATGTGAT<br>GGTGGTGGTGGTTCTGGTGGTGGTGGTTCTGGTGGTGGTGGTTCT<br>GCCCGGCAGAACCACCTGTTCGAGCAGTATCGGGAGAAGAGGCCTGAAGCGGCACAGCTCTTGGA<br>GGATGTTCAGGCCGCCCTGAAGCCATTTTCAGTAAAATTAAATGAGACCACGGATCCCGATAAAAAG<br>CAGATGCTCGAACGGATTCAGCACGCAGTGCAGCTTGCCACAGAGCCACTTGAGAAAGCTGTGCAG<br>TCCAGACTCACGGGAGAGGAAGTCAACAGCTGTGTGGAGGTGTTGCTGGAAGAAGCCAAGGATTTG<br>CTCTCTGACTGGCTGGATTCTACACTTGGCTGTGATGTCACTGACAATTCCATCTTCTCCAAGCTGCC<br>CAAGTTCTGGGAGGGGGACTTCCACAGAGACATGGAA<br>CTCGAGCACCACCACCACCACCACTGA
pET28a-E(R)UCV → The sequence of 6xHis-E7$_{43-62}$-(EAAAK)$_3$-UNE-C1-6xHis as shown in left was inserted into pET28a back bone using NdeI and XhoI.21.195	TATACCATGGGCAGCAGCCATCATCATCATCATCACAGCAGCGGCCTGGTGCCGCGCGGCAGC<br>CATATG<br>GGTCAAGCTGAACCAGATCGTGCTCACTATAACATTGTTACTTTCTGTTGTAAATGTGAT<br>GAAGCTGCTGCTAAAGAAGCTGCTGCTAAAGAAGCTGCTGCTAAA<br>GCCCGGCAGAACCACCTGTTCGAGCAGTATCGGGAGAAGAGGCCTGAAGCGGCACAGCTCTTGGA<br>GGATGTTCAGGCCGCCCTGAAGCCATTTTCAGTAAAATTAAATGAGACCACGGATCCCGATAAAAAG<br>CAGATGCTCGAACGGATTCAGCACGCAGTGCAGCTTGCCACAGAGCCACTTGAGAAAGCTGTGCAG<br>TCCAGACTCACGGGAGAGGAAGTCAACAGCTGTGTGGAGGTGTTGCTGGAAGAAGCCAAGGATTTG<br>CTCTCTGACTGGCTGGATTCTACACTTGGCTGTGATGTCACTGACAATTCCATCTTCTCCAAGCTGCC<br>CAAGTTCTGGGAGGGGGACTTCCACAGAGACATGGAA<br>CTCGAGCACCACCACCACCACCACTGA
pET28a-O(F)UCV → The sequence of 6xHis-OVA$_{247-264(AAAAK)}$-(GGGGS)$_3$-UNE-C1-6xHis as shown in left was inserted into pET28a back bone using NdeI and XhoI. 21006.25	TATACCATGGGCAGCAGCCATCATCATCATCATCACAGCAGCGGCCTGGTGCCGCGCGGCAGC<br>CATATG<br>GATGAAGTTTCTGGTTTAGAACAATTAGAATCTATTATTAACTTCGAAAAATTAGCTGCTGCTGCTGCT<br>AAA<br>GGTGGTGGTGGTTCTGGTGGTGGTGGTTCTGGTGGTGGTGGTTCT<br>GCCCGGCAGAACCACCTGTTCGAGCAGTATCGGGAGAAGAGGCCTGAAGCGGCACAGCTCTTGGA<br>GGATGTTCAGGCCGCCCTGAAGCCATTTTCAGTAAAATTAAATGAGACCACGGATCCCGATAAAAAG<br>CAGATGCTCGAACGGATTCAGCACGCAGTGCAGCTTGCCACAGAGCCACTTGAGAAAGCTGTGCAG<br>TCCAGACTCACGGGAGAGGAAGTCAACAGCTGTGTGGAGGTGTTGCTGGAAGAAGCCAAGGATTTG<br>CTCTCTGACTGGCTGGATTCTACACTTGGCTGTGATGTCACTGACAATTCCATCTTCTCCAAGCTGCC<br>CAAGTTCTGGGAGGGGGACTTCCACAGAGACATGGAA<br>CTCGAGCACCACCACCACCACCACTGA
pET28a-O(R)UCV → The sequence of 6xHis-OVA$_{247-264(AAAAK)}$-(EAAAK)$_3$-UNE-C1-6xHis as shown in left was inserted into pET28a back bone using NdeI and XhoI. 21471.97	TATACCATGGGCAGCAGCCATCATCATCATCATCACAGCAGCGGCCTGGTGCCGCGCGGCAGC<br>CATATG<br>GATGAAGTTTCTGGTTTAGAACAATTAGAATCTATTATTAACTTCGAAAAATTAGCTGCTGCTGCTGCT<br>AAA<br>GAAGCTGCTGCTAAAGAAGCTGCTGCTAAAGAAGCTGCTGCTAAA<br>GCCCGGCAGAACCACCTGTTCGAGCAGTATCGGGAGAAGAGGCCTGAAGCGGCACAGCTCTTGGA<br>GGATGTTCAGGCCGCCCTGAAGCCATTTTCAGTAAAATTAAATGAGACCACGGATCCCGATAAAAAG<br>CAGATGCTCGAACGGATTCAGCACGCAGTGCAGCTTGCCACAGAGCCACTTGAGAAAGCTGTGCAG<br>TCCAGACTCACGGGAGAGGAAGTCAACAGCTGTGTGGAGGTGTTGCTGGAAGAAGCCAAGGATTTG<br>CTCTCTGACTGGCTGGATTCTACACTTGGCTGTGATGTCACTGACAATTCCATCTTCTCCAAGCTGCC<br>CAAGTTCTGGGAGGGGGACTTCCACAGAGACATGGAA<br>CTCGAGCACCACCACCACCACCACTGA

Table S3. The amino sequences of SLPs and epitope peptides used in this study.

Type	HPV 16 Protein	Start	End	Peptide sequence
SLP	E6	23	42	CTELQTTIHDIILECVYCKQ
	E6	43	62	QLLRREVYDFAFRDLCIVYR
	E6	123	143	QRHLDKKQRFHNIRGRWTGRC
	E7	5	25	TPTLHEYMLDLQPETTDLYCY
	E7	43	62	GQAEPDRAHYNIVTFCCKCD
	E7	76	95	IRTLEDLLMGTLGIVCPICS
Epitope peptide	E6	29	37	TIHDIILEC
	E6	49	57	VYDFAFRDL
	E6	129	138	KQRFHNIRGR
	E7	11	19	YMLDLQPETT
	E7	49	57	RAHYNIVTF
	E7	82	90	LLMGTLGIV

Table S4. The DNA sequences of PCR primers used in this study.

Target	Sequence (5' → 3')
Mus IFN-γ	F: ACTGGCAAAAGGATGGTGAC<br>R: TGAGCTCATTGAATGCTTGG
Mus Perforin	F: GATGTGAACCCTAGGCCAGA<br>R: GGTTTTTGTACCAGGCGAGA
Mus Granzyme B	F: GACAACACTCTTGACGCTGG<br>R: TGATCTCCCCTGCCTTTGTCC
Mus CXCL-9	F: CTTGAGCCTAGTCGTGATAAC<br>R: CCAGCTTGGTGAGGTCTATC
Mus Il12b	F: CCAGAGACATGGAGTCATAG<br>R: AGATGTGAGTGGCTCAGAGT
Mus IL-2	F: GCGGCATGTTCTGGATTTGACTC<br>R: CCACCACAGTTGCTGACTCATC
Mus Granulysin	F: GATAAGCCCACCCAGAGAAGTG<br>R: CGTGACCTCCCCGTCCTA
Mus FasL	F: CACAAATCTGTGGCTACCG<br>R: GCCCATATCTGTCCAGTAG
Mus CXCL10	F: CCACGTGTTGAGATCATTGC<br>R: AGTAGCAGCTGATGTGACG
Mus CD69	F: AGGCTTGTACGAGAAGTTGGA<br>R: AGTTCACCAGAATATCGCTTCAG
Mus tbx21	F: CAACCAGCACCAGACAGAGA<br>R: CCACATCCACAAACATCCTG
Mus GAPDH	F: TCAATGAAGGGGTCGTTGAT<br>R: CGTCCCGTAGACAAAATGGT
