# Supplementary material for: Specific targeting of cancer vaccines to antigen-presenting cells via an endogenous TLR2/6 ligand derived from cysteinyl-tRNA synthetase 1
Source: Mol Ther. 2024 Jul 25;32(10):3597–617. doi: 10.1016/j.ymthe.2024.07.014 (PMC11489552; doi:10.1016/j.ymthe.2024.07.014)
Supplement: Document S2. Article plus supplemental information [file mmc2.pdf]

Molecular Therapy
Original Article

Specific targeting of cancer vaccines to antigen-presenting cells via an endogenous TLR2/6 ligand derived from cysteinyl-tRNA synthetase 1

Hyeong Yun Kim,[1,8] Seongmin Cho,[1,8] Sang Bum Kim,[6] Ee Chan Song,[1] Wonchul Jung,[1] Yun Gyeong Shin,[1] Ji Hun Suh,[1] Jihye Choi,[1] Ina Yoon,[2] Uijoo Kim,[1] Hamin Ban,[1] Sunkyo Hwang,[1] Jeongwon Mun,[1] Joohee Park,[1] Nayoung Kim,[1] Youngjin Lee,[7] Myung Hee Kim,[7] and Sunghoon Kim[1,3,4,5]

[1]Institute for Artificial Intelligence and Biomedical Research (AIBI), Medicinal Bioconvergence Research Center, College of Pharmacy, Yonsei University, Incheon 21983, Republic of Korea; [2]Yonsei Institute of Pharmaceutical Sciences, College of Pharmacy, Yonsei University, Incheon 21983, Republic of Korea; [3]College of Medicine, Gangnam Severance Hospital, Yonsei University, Seoul 06273, Republic of Korea; [4]Institute for Convergence Research and Education in Advanced Technology, Yonsei University, Incheon 21983, Republic of Korea; [5]Interdisciplinary Graduate Program in Integrative Biotechnology & College of Medicine, Gangnam Severance Hospital, Yonsei University, Incheon 21983, Republic of Korea; [6]College of Pharmacy, Sahmyook University, Seoul 01795, Republic of Korea; [7]Microbiome Convergence Research Center, Korea Research Institute of Bioscience and Biotechnology (KRIBB), Daejeon 34141, Republic of Korea

Cancer vaccines have been developed as a promising way to boost cancer immunity. However, their clinical potency is often limited due to the imprecise delivery of tumor antigens. To overcome this problem, we conjugated an endogenous Toll-like receptor (TLR)2/6 ligand, UNE-C1, to human papilloma virus type 16 (HPV-16)-derived peptide antigen, E7, and found that the UNE-C1-conjugated cancer vaccine (UCV) showed significantly enhanced antitumor activity *in vivo* compared with the noncovalent combination of UNE-C1 and E7. The combination of UCV with PD-1 blockades further augmented its therapeutic efficacy. Specifically, the conjugation of UNE-C1 to E7 enhanced its retention in inguinal draining lymph nodes, the specific delivery to dendritic cells and E7 antigen-specific T cell responses, and antitumor efficacy *in vivo* compared with the noncovalent combination of the two peptides. These findings suggest the potential of UNE-C1 derived from human cysteinyl-tRNA synthetase 1 as a unique vehicle for the specific delivery of cancer antigens to antigen-presenting cells via TLR2/6 for the improvement of cancer vaccines.

INTRODUCTION

Aminoacyl tRNA synthetases (ARSs) are essential enzymes in protein synthesis, connecting amino acids to their corresponding tRNAs.[1] Some eukaryotic ARSs have diversified their roles, often acquiring new domains during evolution, which can be shared across multiple or specific ARSs. For instance, tryptophanyl-tRNA synthetase 1 binds to Toll-like receptor 4 (TLR4)/myeloid differentiation factor 2, whereas glycyl-tRNA synthetase 1 binds to cadherin EGF LAG seven-pass G-type receptor 2 on immune cells, both modulating immune activities.[2,3] In addition, human cysteinyl-tRNA synthetase 1 (CARS1) possesses unique domains (UNE-C1 and UNE-C2) with distinct roles not found in other tRNA synthetases.[4] Previous research has highlighted the significance of UNE-C1 in CARS1 catalytic activity, its potential for TLR 2/6-mediated immune stimulation, and its impressive thermostability.[5] However, the potential utilization of UNE-C1 as cancer vaccines with superior characteristics remains largely unexplored, including their application in boosting the efficacy of anti-tumor vaccines through precisely targeting dendritic cells (DCs), and improving the uptake and stability of peptide vaccines.

Cancer vaccine development has traditionally focused on protein-based antigens, which expose multiple epitopes. This exposure potentially leads to immune tolerance or even elevates the risk of autoimmunity as potential side effects.[6] To address these concerns, a synthetic long peptide (SLP) vaccine was introduced, aiming to mitigate potential risks and improve efficacy. SLP vaccines have become the preferred choice for tumor antigens in various vaccine studies, and this preference is attributed to their ability to enhance antigen processing and presentation, boost immunogenicity, and lower the risk of resistance.[7,8] However, while SLP vaccines elicit immune responses in clinical trials, significant clinical benefits are often lacking. These clinical failures are mainly considered to result from insufficient uptake into lymphatic systems and a short *in vivo* antigen half-life.[9] When peptide vaccines with low molecular size are subcutaneously administered, they are prone to entering the bloodstream rather than the lymphatic system. These vaccines may be taken up by antigen-presenting cells (APCs) in remote lymphoid organs, which

Received 5 January 2024; accepted 18 July 2024;
https://doi.org/10.1016/j.ymthe.2024.07.014.

[8]These authors contributed equally

Correspondence: Sunghoon Kim, PhD, Professor and Director. Medicinal Bioconvergence Research Center Institute for Artificial Intelligence and Biomedical Research College of Pharmacy & College of Medicine The Interdisciplinary Graduate Program in Integrative Biotechnology Yonsei University 85 Songdogwahak-ro, Yeonsu-gu, Incheon 21983, Korea
E-mail: sunghoonkim@yonsei.ac.kr

Published by Elsevier Inc. on behalf of The American Society of Gene and Cell Therapy.
This is an open access article under the CC BY license (http://creativecommons.org/licenses/by/4.0/).

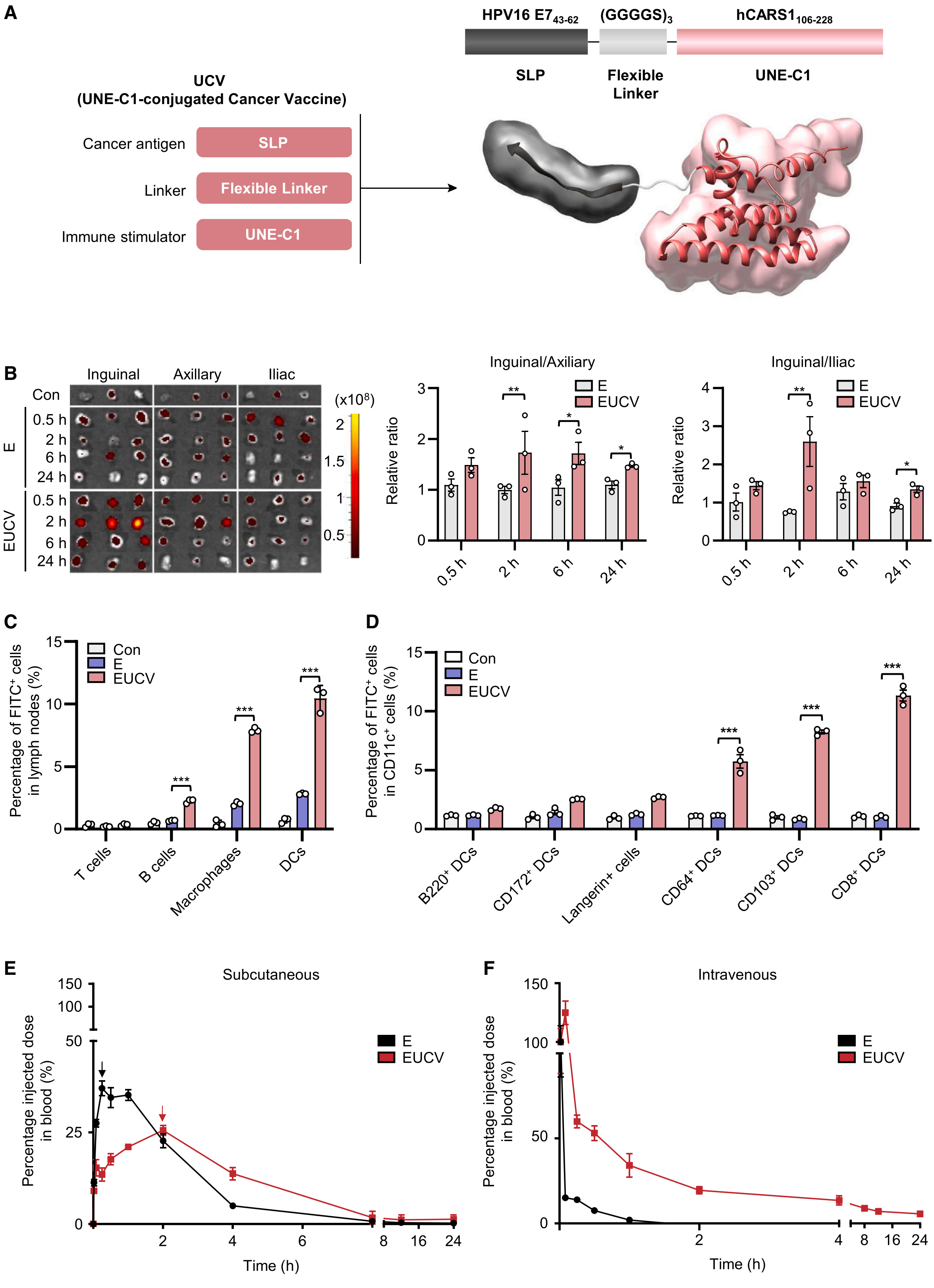

(legend on next page)

could potentially foster immune tolerance instead of eliciting a robust, antigen-specific immune response against the designated antigen.[10,11] Moreover, peptide and protein antigens entering the bloodstream can be degraded by various proteolytic enzymes, potentially diminishing their stable supply to APCs. One of the promising strategies devised to overcome the bioavailability problem of peptide vaccines, especially regarding organ-specific targeted delivery, is the use of carrier proteins.[12,13] This strategy significantly reduces antigen exposure to the bloodstream and enables the vaccine to concentrate in the lymph nodes (LNs), thus promoting an optimal antigen-specific immune response.

APCs present in LNs play a central role in eliciting specific T cell responses to exogenous antigens. Hence, the coordinated delivery of antigens and immunostimulants to the LNs is crucial to preventing immune tolerance and enhancing efficacy.[14] To deliver vaccines accumulated in the LNs to DCs, attempts have been made to link antigens with guide peptides or pattern recognition receptor ligands to target cell surface receptors. However, because receptor expression varies with DC subtype, target receptor expression on specific cells must be considered to elicit an optimal antigen-specific immune response. To stimulate an antigen-specific immune response, accompanying antigen delivery with immune stimulation, such as costimulation factors or cytokine secretion, is essential. Hence, the selection of an adjuvant must match the receptor expression levels of the targeted cells, ensuring that antigen delivery and effective immune responses occur within the same cells.[15] Injected vaccines can be taken up by migratory APCs or transported via lymphatic vessels to resident APCs in LNs.[16] Specifically, CD11c$^+$ and CD8$^+$ type 1 conventional dendritic cells (cDC1s) excel in cross-presentation, cytokine production, and costimulatory molecule expression, efficiently regulating adaptive immunity.[17] cDC1s play an important role in activating antitumor immunity, and their performance correlates with improved patient outcomes.[18] Collectively, efficient delivery systems and targeted stimulation of specific cells are emphasized as essential factors for improving the efficacy of cancer vaccines.

In this study, we explored the applicability potential of a novel immune booster for cancer vaccines by using the unique immune-stimulating activity and physicochemical properties of UNE-C1. To achieve this, we developed a conjugate vaccine by covalently linking UNE-C1 to SLPs and evaluated its efficacy in a mouse model of cervical cancer. The conjugate vaccine, inheriting the characteristics of UNE-C1, accumulates in draining lymph nodes (dLNs) and activates or internalizes TLR2-expressing APCs. Combining the vaccine with PD-1 inhibitors synergistically improved survival rates and achieved complete regression (CR). These findings highlight the potential of UNE-C1 as an immune-stimulating vehicle to deliver cancer antigens to APCs for improved therapeutic efficacy.

RESULTS

Construction and characterization of UNE-C1-conjugated cancer vaccine

The therapeutic efficacy of cancer antigens can be enhanced, and their potential adverse effects can be reduced if they are covalently linked to certain immune modulators that can specifically deliver them to APCs.[13] Here, we tested this possibility using UNE-C1, an immune activator via TLR2/6, by coupling it to a cancer antigen derived from human papilloma virus type 16 (HPV-16) via a designed peptide linker (Figure 1A). We then investigated how UNE-C1 conjugation to the antigens would affect the retention of the antigens in the inguinal LN near the injected site through lymphatic drainage, specific delivery of the antigens to DCs, and the priming of the antigen-specific CD8$^+$ T cells.

To construct the optimal UNE-C1-conjugated cancer vaccine (UCV), we screened the SLP antigens derived from HPV-16-derived oncoprotein E6 or E7. We synthesized different E6- and E7-derived SLPs derived from oncoproteins and compared their capability of inducing antigen-specific immune responses. We immunized mice with each of the SLPs alone or in combination with UNE-C1 (U stands for UNE-C1 hereafter).[19] We observed that the highest interferon gamma (IFN-γ) activity was observed in the mice administered the $E7_{43-62}$ SLP and U (Figure S1A). We then connected the $E7_{43-62}$ SLP to U with either a rigid “EAAAK” or flexible “GGGGS" peptide linker and treated them to phorbol 12-myristate 13-acetate (PMA)-differentiated THP-1 cells to measure the induced tumor necrosis factor alpha (TNF-α) secretion.[20] While the $E7_{43-62}$ SLP and U linked with GGGGS showed comparable activity with U alone, the two peptides linked with EAAAK slightly reduced the TNF-α-inducing activity (Figure S1B). We thus selected GGGGS peptide linkers for the construction of the conjugated vaccine, designated as

Figure 1. Design of the UNE-C1-conjugated cancer vaccine and its biodistribution and pharmacokinetic profile

(A) We designed the conjugated cancer vaccines to covalently link the cancer antigens, linkers, and immune stimulators. For example, we selected $E7_{43-62}$ from HPV-16 oncoprotein E7 as the cancer antigen, the three repeats of (GGGGS) as the linker peptide, and U obtained from human CARS1 as the immune stimulator and fused them into one peptide entity (upper panel). This conjugated cancer vaccine is expected to show enhanced anticancer efficacy with increased local retention in draining lymph nodes, specific delivery to DCs, and enhanced priming of CD8$^+$ T cells (lower panel). (B–D) C57BL/6 mice were subcutaneously injected with 5 nmol of FAM-labeled E or EUCV. (B) Inguinal, axillary, and iliac lymph nodes were excised, and the presence of each peptide was monitored by fluorescence intensity through IVIS at the indicated time interval (n = 3 per group). The fluorescence signals in axillary or iliac lymph nodes were determined as ratios to those in the inguinal lymph node (n = 3 per group). (C and D) After 2 h of immunization, immune cells were harvested from the excised inguinal lymph nodes, and the 5-FAM signals in T cells, B cells, macrophages and DCs, and DC subtypes were assessed via flow cytometry (n = 3 per group). (E and F) 5-FAM-labeled E or EUCV were either (E) subcutaneously or (F) intravenously injected into C57BL/6 mice (n = 3 per group). Less than 50 μL of blood was collected, and the antigen concentration in plasma was quantified for 24 h after injection. The data were used to determine drug concentrations in plasma over time (fit data ± mean with error). Data are representative of three independent experiments, and the results are presented as the mean ± SEM. Statistical significance was analyzed using Student’s t test (*$p < 0.05$, **$p < 0.01$, ***$p < 0.001$). U, UNE-C1; E, HPV-16 $E7_{43-62}$ peptide; EUCV, HPV-16 $E7_{43-62}$ peptide conjugated UNE-C1; IVIS, *in vivo* imaging system; DCs, dendritic cells.

EUCV, for further experiments (E stands for $E7_{43-62}$ hereafter). To determine whether U can generally be used for other cancer antigens, U was fused to the ovalbumin (OVA) epitope (SIINFEKL) using the same linker (O stands for $OVA_{247-264(AAAAK)}$ hereafter), and it was confirmed that each EUCV and OUCV protein was stably produced (Figures S1C and S18). We then observed that the OUCV conjugate vaccine also induced a comparable level of immune activity with U or EUCV (Figure S1D). Given that U has high thermal stability, we examined if this characteristic was also displayed by the conjugated vaccines EUCV and OUCV. Both maintained immune activity (Figure S1D) and circular dichroism (CD) spectra (Figure S1E), even after boiling, confirming the thermal stability of the conjugated vaccines. To rule out the possibility of lipopolysaccharide (LPS) contamination, we treated EUCV and U with polymyxin B or protease K and reexamined their TNF-α-inducing activity. The results showed that only proteinase K treatment abated the activity, indicating that the immune response triggered by EUCV was attributed to U but not LPS contamination (Figure S1F). To determine whether the two conjugated vaccines would work via TLR2/6 as U, we treated them to HEK cells expressing each of TLR2/6, TLR2/1, and TLR4. We found that, like U and Pam2CSK4 (a known TLR2/6 agonist), they induced nuclear factor κB in TLR2/6-expressing cells but not in other cells (Figures S1G–S1I). TLR agonists play a crucial role in activating both the innate and adaptive immune systems by inducing inflammatory cytokines and costimulatory molecules.[21] To explore the ability of EUCV to stimulate APCs, we exposed bone marrow-derived dendritic cells (BMDCs) to E alone, E+U (combination), and EUCV in equivalent doses. After 24 h, we found that EUCV and U induced the costimulatory molecules CD80 and CD40 at comparable levels, which is crucial for inducing an adaptive immune response (Figures S2A and S2B). Moreover, both EUCV and U also triggered the secretion of pro-inflammatory cytokines, IL-12p70 and IL-6, while this response was absent in the E group (Figures S2C and S2D). The expression of other inflammatory markers was also observed to be increased in UCVs based on E or O antigens in a dose-dependent manner (Figures S2E–S2H). These findings suggest that UCV can stimulate APCs, promoting innate immunity through upregulated costimulatory molecules and secreted pro-inflammatory cytokines.

Enhanced retention of UCVs in the lymphatic system

Considering the challenges posed by systemic antigen degradation and absorption after vaccination,[13] we examined whether the conjugation of U to the antigen can alleviate these limitations. To monitor the biodistribution of the cancer vaccines, we labeled E and EUCV with FAM, administered them via subcutaneous routes, and monitored their distributions over time. While E exhibited diffused distribution to axillary and iliac LNs beyond inguinal LNs near the injection site, EUCV was preferentially detected in the inguinal dLNs. The higher retention of EUCV apparently results from its larger size compared with E alone.[13] Notably, the signals from EUCV persisted for up to 24 h, indicating a prolonged residence within the inguinal dLNs (Figure 1B). The extended residence of the conjugated vaccine in the inguinal dLNs substantially increased the preferred uptake of E to macrophages and DCs in this region, highlighting the specificity of EUCV to APCs (Figure 1C). Interestingly, while a 2.5% higher signal was observed in DCs compared with macrophages at 2 h after administration, a 3.4% higher signal was observed in macrophages compared with that in DCs at 24 h (Figure S3A). This suggests that the proteins taken up are processed more rapidly in DCs than in macrophages, resulting in a lower signal at later time points.[22] To determine whether selective antigen uptake of UCVs at APCs, such as DCs and macrophages, is influenced by TLR2/6 expression levels, we measured TLR2/6 expression in T cells, B cells, macrophages, and DCs residing in the inguinal dLNs. The results revealed that both macrophages and DCs exhibited relatively high TLR2 and TLR6 expression levels, with DCs displaying higher TLR2 expression than macrophages (Figure S4A). Subsequently, we subcutaneously administered FAM-labeled E and EUCV to mice to investigate antigen uptake to cDC1, which is known for its role in cross-presentation. The investigation into antigen uptake was conducted among various DC subtypes in the LNs, including cDC1, with their compositions distinguished as follows: $CD8^+$ DCs ($CD8^+$ cDC1), $CD103^+$ DCs ($CD103^+$ cDC1), $CD172^+$ DCs (cDC2), $B220^+$ DCs (plasmacytoid DCs, pDCs), $Langerin^+$ cells (Langerhans cells), and $CD64^+$ DCs (monocyte-derived DCs). Antigen uptake was significantly enhanced in the $CD8^+$ DCs, $CD103^+$ DCs, and $CD64^+$ DCs of the EUCV-administered mouse group but not in $B220^+$ DCs, $CD172^+$ DCs, and $Langerin^+$ cells (Figures 1D and S5A). These three cell types play a crucial role in cytotoxic T lymphocyte (CTL) responses against cancer, especially with $CD8^+$ and $CD103^+$ DCs sharing functional homology with human cDC1 ($CD141^+$ DCs).[23,24] We also examined the differences in antigen uptake among DC subtypes in relation to TLR2/6 expression levels, confirming that $CD8^+$ DCs, $CD103^+$ DCs, and $CD64^+$ DCs displayed higher TLR2/6 expression levels than other DCs (Figure S4B). This suggests that antigen uptake in different DCs is influenced by the expression levels of TLR2/6, which is the target receptor for UCV.

Next, to measure the blood concentrations of vaccines administered via the subcutaneous route, blood samples were collected via orbital hemorrhage at the indicated time points and used to measure the blood levels of E and EUCV. The results showed that the concentration of E peaked at 0.25 h after the injection, whereas that of EUCV peaked at 2 h, indicating a delayed systemic distribution of the conjugated vaccine (Figure 1E). When administered intravenously, the half-life of EUCV (0.334 h) was approximately 15.9 times longer than that of E (Figure 1F). These results suggest that E is rapidly distributed throughout the body, regardless of the route, whereas EUCV is well retained in the lymphatic system when administered subcutaneously and shows an extended half-life in the blood compared with E alone.

To investigate the effects of U on antigen distribution, we subcutaneously injected mice with each of FAM-labeled E, E++U, and EUCV and analyzed the inguinal dLNs 24 h later. A 4.4-fold stronger E signal was observed in the LNs from the mice injected with E++U than in those with E alone (Figure S6A). This result appears to result from

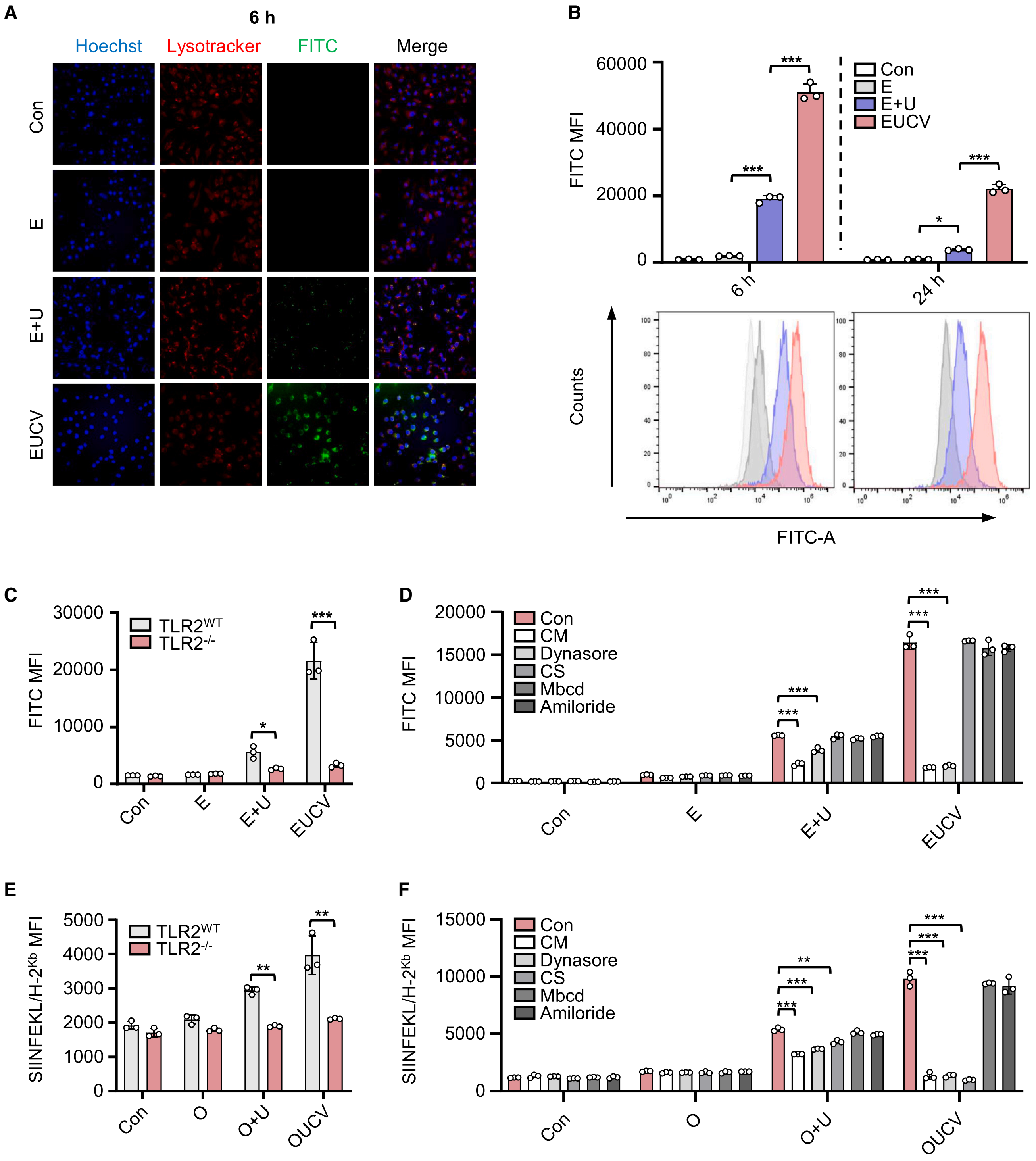

Figure 2. Pathway analysis of the UCV for antigen internalization and antigen presentation

(A and B) 5-FAM-labeled E and EUCV were treated to BMDCs for 6 and 24 h (each 100 nM). (A) The cells with the internalized and surface-presented antigen were visualized and analyzed using confocal microscopy and (B) flow cytometry, respectively. (C) BMDCs from naive or TLR2$^{-/-}$ C57BL/6 mice were treated with 5-FAM-labeled E, E+U, and EUCV for 24 h. Antigen uptake was analyzed from the CD11c^{+} gating population, and the FITC signal was evaluated using flow cytometry. (D) BMDCs from naive C57BL/6 mice were preincubated with chlorpromazine (CM, 40 μM), dynasore (20 μM), cathepsin S inhibitor (CS, 2 μM), methyl-β-cyclodextrin (Mbcd, 2 μM), and amiloride(2 mM) for 30 min. FAM-labeled E, E+U, and EUCV were treated to BMDCs for 24 h, and antigen uptake was detected using flow cytometry. (E) Antigen presentation was analyzed from

(legend continued on next page)

the enhanced antigen uptake ability of DCs activated by U.[25] Interestingly, the covalent coupling of U with E further increased the E signal 10.8-fold compared with E alone. This additional improvement appears to be attributed to the increased molecular weight of E by covalent linkage to U, which restricted the rapid systematic diffusion of E. In conclusion, these results suggest that the covalent coupling of U to cancer antigens can favor their accumulation in the lymphatic system, providing an enhanced opportunity to stimulate the adaptive immunity required for cancer immunology.

Enhanced antigen uptake and presentation by UCVs

Targeting DCs for antigen delivery enhances vaccination efficacy due to their superior antigen processing and presentation capabilities.[26] To evaluate cellular uptake of the antigen, we labeled E and EUCV with FAM, treated them to BMDCs, and compared the amounts of the labeled antigen in the cells 6 h after treatment. We found that the fluorescence intensity of EUCV was significantly higher than that of E alone and EUCV (Figure 2A). Furthermore, the higher uptake of EUCV was sustained for up to 24 h compared with that of E alone or E+U (Figure 2B). A similar pattern was observed with O+U and OUCV (Figure S7A). This improved antigen uptake is presumably due to TLR2-specific delivery of U-activated DCs.[27] To investigate TLR2 dependency of antigen uptake, BMDCs from TLR2 wild-type (WT) and knockout (KO) mice were treated with E, E+U, and EUCV. Antigen uptake was significantly reduced in TLR2 KO BMDCs compared with that in WT BMDCs, confirming the TLR2 dependency of EUCV uptake (Figure 2C).

To identify the specific endocytic pathway involved in the uptake of the conjugated vaccines, we used specific inhibitors at several concentrations, including the clathrin-mediated endocytosis inhibitor chlorpromazine (CM), the dynamin-dependent endocytosis inhibitor (dynasore), transporters associated with the antigen processing (TAP)-independent major histocompatibility complex class I (MHC class I) cross-presentation inhibitor (cathepsin S inhibitor [CS]), caveolae-mediated endocytosis inhibitor (methyl-β-cyclodextrin [Mbcd]), and macropinocytosis inhibitor (amiloride). At various concentrations, CM and dynasore reduced E+U and EUCV uptake, whereas no change was noted in the groups treated with CS, Mbcd, or amiloride (Figures 2D and S8A). These results suggest that the mechanism underlying UCV uptake can be regulated by clathrin and dynamin. In addition, to examine the processing and presentation pathway of the conjugated vaccine, we employed a CS, an inhibitor of the TAP-independent pathway, and observed no effect on antigen uptake, suggesting that U-mediated antigen uptake is a separate process from antigen processing and presentation.[28]

To further validate the TLR2 dependency of U-mediated antigen uptake and presentation, we used O to determine the effect on antigen presentation and found that O presentation was significantly reduced in TLR2 KO BMDCs compared with that in the TLR2 WT BMDCs (Figure 2E). In addition, antigen presentation was reduced in BMDCs treated with CM and dynasore, confirming that the limitation in uptake led to a reduction in antigen presentation (Figures 2F and S8B). The reduction in antigen presentation upon CS treatment suggests that the antigens conjugated with U are processed via a TAP-independent processing and presentation pathway. Furthermore, the expression of CD80, a key indicator of innate immune stimulation, showed similar patterns across the E+U and EUCV groups as well as the O+U and OUCV groups (Figures S9A and S9B). This suggests that TLR2 is an important mediator in the antigen uptake and cellular activation functions of UCV.

To compare antigen-presenting ability *in vivo*, we subcutaneously administered O, O+U, and OUCV to mice. OUCV-administered mice showed increased antigen presentation of the SIINFEKL epitope through H-$2K^b$ in $CD11c^+$, $CD8^+$, and $CD8^-$ DCs, but not in $B220^+$ DCs, compared with those administered O+U (Figure S9C). These findings support previous reports that $CD8^+$ DCs exhibit stronger responsiveness to TLR2 agonists compared with $B220^+$ DCs.[29] Collectively, the conjugated vaccine demonstrated its effectiveness in promoting intracellular delivery through TLR2/6 and clathrin-dynamin-mediated endocytosis and is processed through the TAP-independent pathway for antigen presentation.

Thereafter, we investigated whether cellular stimulation of UCV work in human immune systems, extending beyond the mouse immune system, by examining human DC subtypes. We isolated human pan-DC (Lin^-, HLA-DR^+, $CD11c^+$, $CD123^+$, $CD1c^+$, and $CD141^+$) from human PBMCs, and subsequently treated it with E, U, E+U, and EUCV at 100 nM, respectively.[30] As positive control, we used LPS. After 24 h treatment, the EUCV group showed enhanced expression levels of activation markers CD80, CD83, and CD86 in human $CD141^+$ DCs (HLA-DR^+, $CD11c^+$, and $CD141^+$), which share functional homology with mouse $CD8^+$ and $CD103^+$ DCs (cDC1) (Figures S10A–S10C).[31] Meanwhile, in the $CD1c^+$ DCs (HLA-DR^+, $CD11c^+$, and $CD1c^+$), which share homology with mouse $CD172^+$ DCs (cDC2), the EUCV group displayed relatively low levels of activation markers, and virtually no activity was observed in the pDCs (HLA-DR^+, $CD11c^-$, and $CD123^+$). To measure the release of inflammatory cytokines, non-DCs and pan-DCs were seeded at equal cell numbers and treated with E, U, E+U, and EUCV at 100 nM each. After 24 h, the released TNF-α was quantified using ELISA, showing relatively higher levels in pan-DCs (Figure S10D). To summarize, UCV can intensively activate human $CD141^+$ DCs, which share

the $CD11c^+$ gating population from BMDCs after pretreatment with 100 nM of O, O+U, or OUCV, and the SIINFEKL/H-$2K^b$ signal was evaluated using flow cytometry. (F) Flow cytometry analysis of SIINFEKL/H-2Kb expression in BMDCs was conducted after pretreating with CM (40 μM), dynasore (20 μM), CS (2 μM), Mbcd (2 μM), and amiloride (2 mM). After 30 min of pretreatment, 100 nM of O, O+U, or OUCV was used for the assay. Data are representative of three independent experiments, and results are presented as the mean ± SD and SEM. Statistical significance was analyzed using Student's t test or two-way ANOVA (*$p < 0.05$, **$p < 0.01$, ***$p < 0.001$). U, UNE-C1; O, $ovalbumin_{247–264A4K}$ peptide; BMDC, bone marrow-derived dendritic cell; CD, cluster of differentiation; LN, lymph node.

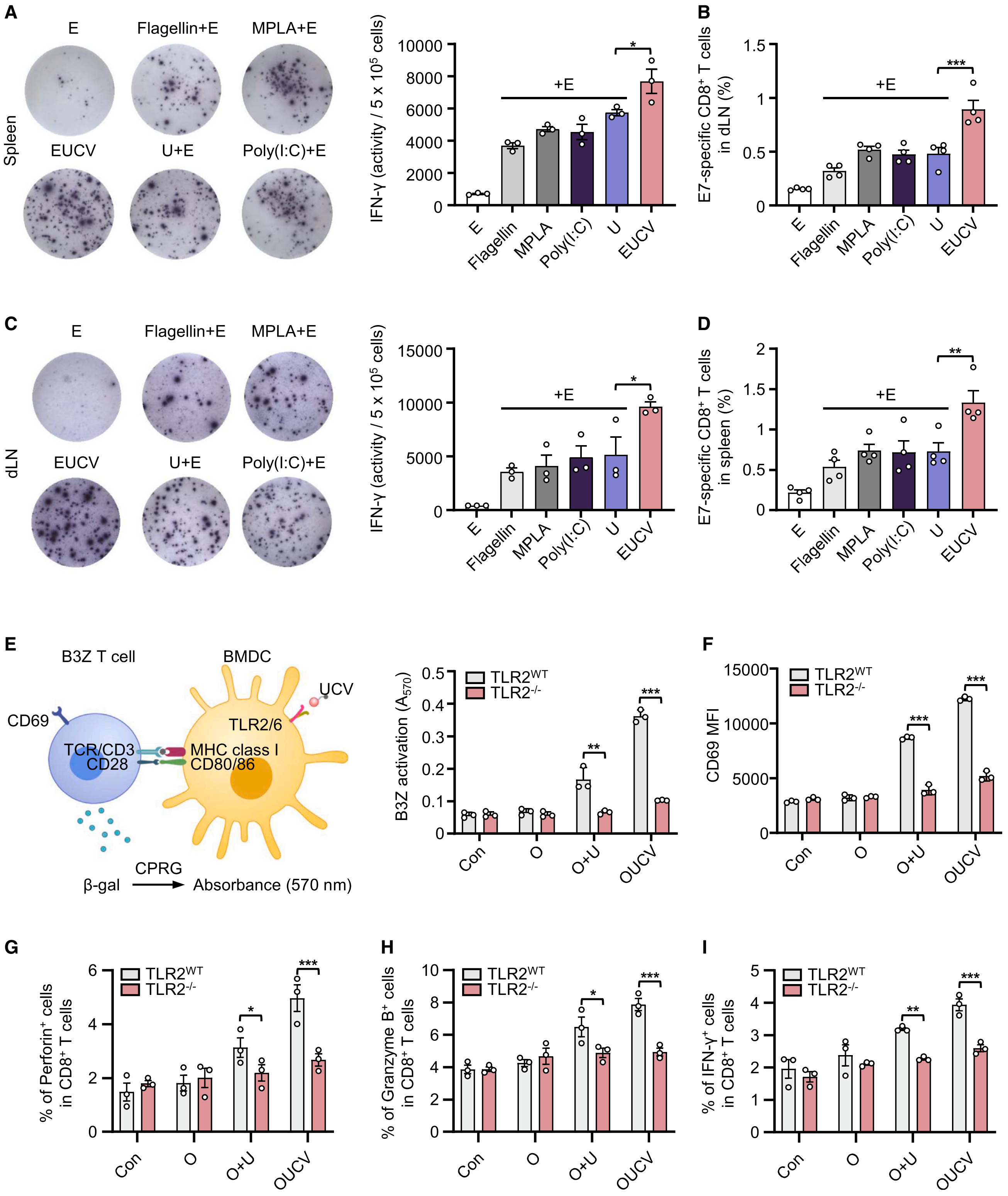

(legend on next page)

functional homology with mouse cDC1, indicating that the immunological outcomes observed in mouse models are potentially applicable to human systems.

Antigen-specific T cell priming elicited by UCVs

Next, we tested the ability of the UCV to induce antigen-specific immune responses using an *ex vivo* IFN-γ enzyme-linked immunospot (ELISpot) assay. We subcutaneously injected the conjugated vaccine and several TLR agonists into mice twice at 1-week intervals. A week after the final administration, we isolated the cells from the spleen or inguinal dLNs and re-stimulated them with the $E7_{49–57}$ epitope to measure levels of IFN-γ expression and antigen-specific T cell priming using the H-$2D^b$ $E7_{49–57}$ tetramer. Consequently, EUCV showed a more robust response than other TLR agonists, specifically inducing 1.64-fold higher expression of IFN-γ and 1.8-fold stronger priming of antigen-specific T cells than E+U in the spleen (Figures 3A and 3B). Enhanced immune response patterns were also consistently observed in the inguinal dLNs, where the conjugated vaccine was localized (Figures 3C and 3D), highlighting the importance of lymphatic circulation for immune cell priming.[32] To examine the impact of the conjugated vaccine on antigen-specific T cell responses, we treated TLR2 WT or TLR2 KO BMDCs with O, O+U, and OUCV for 24 h. These cells were then subjected to a T cell activation assay by co-culturing with pulsed BMDCs and B3Z T cell hybridoma, a $CD8^+$ T cell line with a reporter function triggered by the OVA epitope. After 24 h, T cell activation was quantified by measuring LacZ production using the CPRG substrate.[33] The results demonstrated that OUCV treatment elicited notably stronger T cell activation in TLR2 WT BMDCs than O+U treatment, whereas this activation was absent in TLR2 KO BMDCs (Figure 3E). In addition, the expression of $CD8^+$ T cell functional markers related to $CD8^+$ T cell activation and proliferation (CD69 and IL-2), cytotoxicity (Perforin, Granzyme B, and FasL), and effector function (IFN-γ and TNF-α) was significantly increased by OUCV treatment; however, this effect was abolished in TLR2 KO cells (Figures 3F–3I and S11A). To further elucidate the relationship between antigen delivery within the dLNs by UCV and CD8+ T cell stimulation, we investigated antigen-specific immune responses using a depletion model. In brief, we subcutaneously injected mice with control and clodronate liposomes and, 24 h later, we administered con, E, and EUCV twice at 1-week intervals. A week after the final injection, dLNs analysis revealed clodronate liposome-depleted DCs (Figure S12A). Following the re-stimulation of cells isolated from dLNs with equal cell counts and analysis via ELISpot, the results indicated that the antigen-specific immune response upregulated by EUCV was significantly inhibited by clodronate (Figure S12B). In addition, antigen-specific $CD8^+$ T cell analysis within the dLNs exhibited a significant reduction in the population previously increased by EUCV (Figure S12C). Since clodronate liposomes can be taken up by macrophages as well as DCs, we do not rule out that the depletion of macrophages could also contribute to the efficacy reduction of UCV. Whatever, these results suggest that UCV promotes cross-presentation to $CD8^+$ T cells in a TLR2-dependent manner, indicating that antigen uptake in the dLNs is crucial for this cross-presentation process.

Enhanced therapeutic efficacy of UCVs

Based on the ability of UCVs to induce effective antigen-specific immune responses via the specific delivery of antigens to DCs in a TLR2-dependent manner, we investigated whether these characteristics are reflected as an improved efficacy in mouse tumor models. After implanting TC-1 cells to the right flank of C57BL/6 mice on day 0, we subcutaneously injected saline, E, E+U, and EUCV on day 6 and measured tumor growth at 2-day intervals. Among them, EUCV most effectively suppressed tumor growth (Figure 4A), suggesting that its improved biodistribution and specific antigen delivery were well reproduced as antitumor activity. To further understand the mechanism underlying the antitumor effect shown by UCV, we analyzed tumor-infiltrated lymphocytes (TILs) isolated from tumor tissues. The group treated with EUCV showed an increased number of infiltrated $CD8^+$ T cells and antigen-specific $CD8^+$ T cells (Figures 4B and 4C) compared with those treated with control, E, and E+U. Furthermore, EUCV significantly enhanced the number of activated TNF-α^+ and IFN-γ^+ $CD8^+$ T cells within TILs (Figure S13A). Using isolated tumor tissue, we analyzed gene expression related to cytotoxicity (Perforin, Granzyme B, Granulysin, and FasL), inflammatory or chemotactic cytokines (IFN-γ, CXCL9, CXCL10, and CXCL11), and $CD8^+$ T cell activation and proliferation (CD69, Tbx21, IL-2, and IL-12b). The analysis revealed that mice treated with EUCV had relatively higher gene expression levels than all other groups, and the E+U group also exhibited a significant increase (Figures 4D and S14A). In addition, we also tested our hypothesis using an E.G7-OVA cancer model. A mouse model was constructed by subcutaneously injecting E.G7-OVA cells into the right flank of C57BL/6 mice on day 0. We then subcutaneously delivered saline, O, O+U, and OUCV to the opposite side of the cancer cell injection sites on days 3 and 10. As shown above with EUCV, OUCV mostly

Figure 3. Evaluation of improved antigen-specific $CD8^+$ T cell immune response and TLR2-dependent cross-presentation of UCV

(A and B) (A) C57BL/6 mice were immunized with the indicated reagents on days 0 and 7. On day 14, spleen and dLNs tissues were harvested from immunized mice, and immune cells were isolated. The isolated immune cells (5×10^5 cells) from spleen were *ex vivo* stimulated with the E7 epitope (2 μg/mL) for 48 h and analyzed using an ELISpot reader ($n = 3$ per group). (B) Percentages of E7-specific $CD8^+$ T cells in the spleen were measured using E7 tetramers by flow cytometry ($n = 3$ per group). (C) Immune cells (5×10^5 cells) isolated from dLNs were stimulated *ex vivo* with the E7 epitope for 48 h and analyzed using an ELISpot reader ($n = 3$ per group). (D) Percentages of E7-specific $CD8^+$ T cells in the dLNs were measured using E7 tetramers by flow cytometry ($n = 3$ per group). (E–I) BMDCs from TLR2 WT and $TLR2^{-/-}$ mice were incubated for 24 h with O, O+U, or OUCV and co-cultured overnight in the presence of B3Z $CD8^+$ T cells. T cell activation was determined based on (E) β-galactosidase activity, (F) CD69 expression, (G) $Perforin^+$, (H) Granzyme B^+, and (I) IFN-γ^+ $CD8^+$ T cell population via absorbance measurement and flow cytometry, respectively. Data are representative of three independent experiments, and the results are presented as the mean ± SEM. Statistical significance was analyzed using Student's t test ($*p < 0.05$, $**p < 0.01$, $***p < 0.001$). B3Z, B3xZ.8 $CD8^+$ T cells; CPRG, chlorophenol red-β-D-galactopyranoside; TCR, T cell receptor; E7 epitope, HPV16 $E7_{49–57}$.

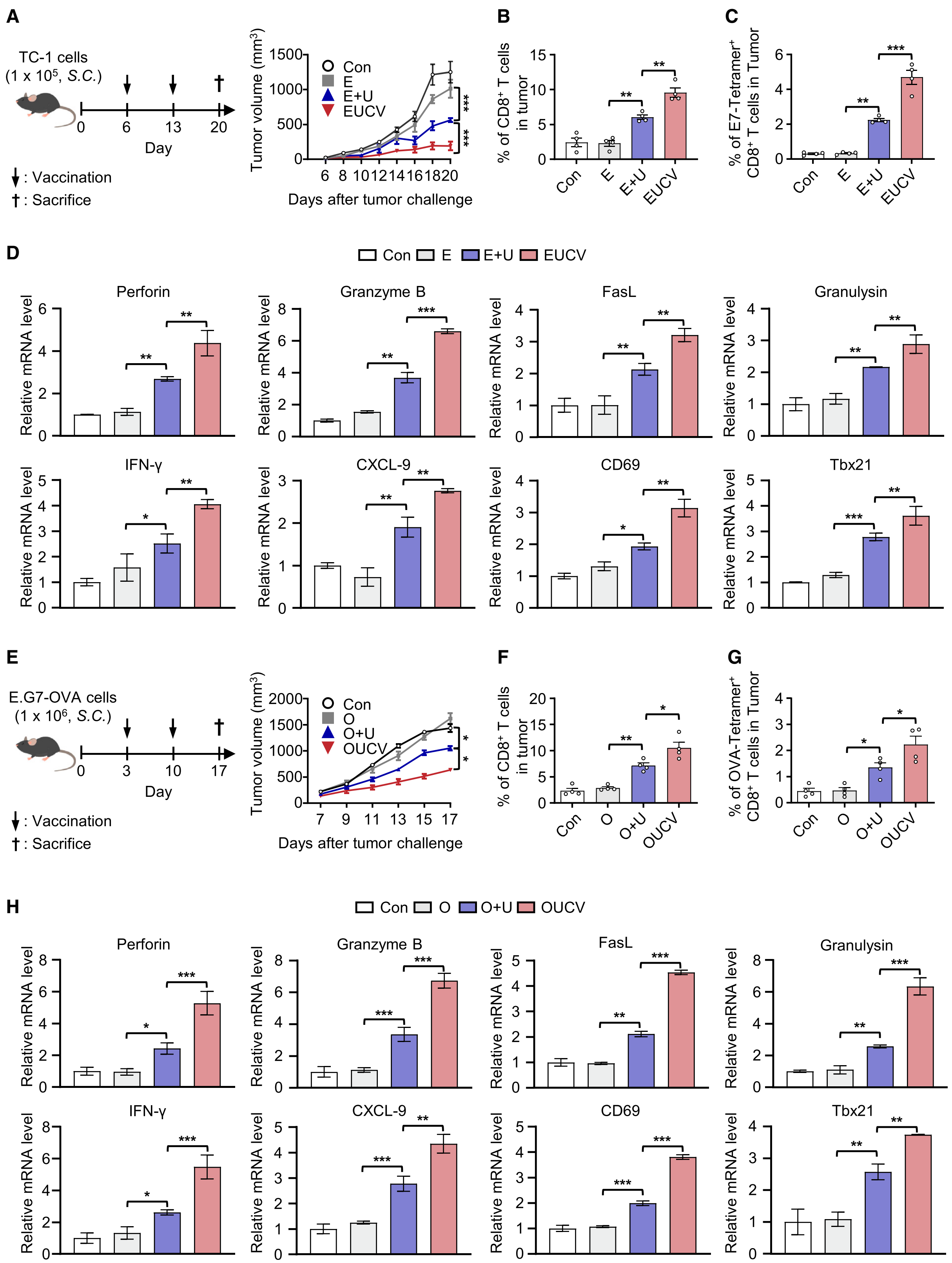

(legend on next page)

strongly suppressed tumor growth (Figure 4E), showing the highest numbers of infiltrated $CD8^+$ T cells and antigen-specific $CD8^+$ T cells (Figures 4F and 4G). We also isolated tumor tissue from mice to analyze the same genes as in the previous analysis, and the results indicated that most of these genes were upregulated in the OUCV-treated group (Figures 4H and S14B). These data suggest that UCVs could promote robust $CD8^+$ T cell priming through cross-presentation both *in vitro* and *in vivo*, leading to tumor regression via the recruitment and activation of antigen-specific $CD8^+$ T cells.

Synergistic efficacy of UCV and anti-PD-1 blockade

Immune checkpoint inhibitors (ICIs) have shown efficacy against cervical cancer by targeting PD-1, PD-L1, and CTLA-4.[34] However, their use as monotherapy may result in T cell dysfunction and impair clinical potency, prompting exploration into combination therapies with other immunotherapies or chemotherapy.[35,36] Therefore, we tested whether EUCV could synergize with PD-1 ICIs. We administered E, E+αPD-1 blockade, EUCV, and EUCV+αPD-1 blockade to mice with TC-1 tumors and monitored tumor growth. Neither E alone nor the combination of E+αPD-1 blockade significantly suppressed tumor growth. While EUCV showed strong tumor-suppressive activity, it failed to eliminate residual tumor growth. In contrast, combined treatment with EUCV and αPD-1 blockade completely inhibited tumor growth during the observation period (Figure 5A). While the E– and E+αPD-1-treated mouse groups experienced 100% mortality by day 26, the EUCV-treated group exhibited 25% survival until day 60, with 12.5% of CR (Figure 5B). In the EUCV+αPD-1-treated group, final survival was sustained at 50%, with 37.5% of CR (Figure 5C). These results suggest that EUCV can enhance the limited efficacy of PD-1, at least in the cervical cancer mouse model. To investigate the synergistic effect of EUCV and αPD-1 blockade in more detail, we analyzed the expression of the genes related to $CD8^+$ T cell functionality. The combination of EUCV and αPD-1 blockade induced the expression of various genes involved in $CD8^+$ T cell effector functions (Figures 5D and S15A).[37] Immunohistochemistry revealed significantly increased T cell infiltration inside the tumor in the EUCV+αPD-1 group, and the ratio of functional T cells was also significantly higher than in the other groups (Figure 5E). Therefore, the combination of EUCV and αPD-1 blockade could induce synergistic antitumor efficacy by enhancing extended T cell function in the tumor microenvironment (TME).

Toxicity analysis of UCV

TLR expression is observed in various tissues and cells, implying that TLR ligand exposure to the entire system can cause toxicity.[38] Therefore, for the successful development of cancer vaccines in clinical settings, a TLR ligand-conjugated cancer vaccine with *in vivo* safety and efficacy is warranted. To determine whether UCV induces cytotoxicity by binding to its target receptor, we examined TLR2/6 expression levels in various cell lines *in vitro*. This evaluation involved the mouse cancer cell lines E.G7-OVA (lymphoma) and TC-1 (lung cancer), along with the following immune cell lines: EL4 (T cells), A20 (B cells), Raw 264.7 (macrophages), and DC2.4 (dendritic cells). The results revealed elevated TLR2 expression in DC2.4, Raw 264.7, and TC-1 cells as well as significantly increased TLR6 expression exclusively in DC2.4 and Raw 264.7 cells (Figure S16A). Thereafter, we assessed the *in vitro* cytotoxicity of EUCV in DC2.4, Raw 264.7, and TC-1 cells, which exhibited significant TLR2/6 expression. While doxorubicin exhibited concentration-dependent toxicity across all three cell lines, EUCV did not display toxicity at any concentration (Figure S16B). To evaluate the *in vivo* safety of UCV, we administered EUCV at 25 mpk (25.2 nmol) and 100 mpk (100.6 nmol) to mice. We used TLR2/6 agonist Pam2CSK4 (100.6 nmol), TLR3 agonist poly(I:C) (2.5 nmol), U (100.6 nmol), and E (100.6 nmol) as controls. EUCV did not affect body weight at either dose, while Pam2CSK4 induced weight loss and splenomegaly (Figures S17A and S17B). Furthermore, on analyzing bloodstream cytokines to assess systemic toxicity, significant inflammatory cytokine expression was observed in the poly(I:C) and Pam2CSK4 groups, whereas EUCV did not induce systemic toxicity at either dose (Figure S17C). These results suggest that UCV is a potential novel cancer vaccine with a higher safety profile *in vitro* and *in vivo* than other agonists.

DISCUSSION

Effective cancer vaccines rely on precise targeting of antigen delivery and immunostimulants to elicit long-lasting anticancer immunity. While several types of APCs are involved in antigen presentation, achieving optimal efficacy necessitates the specific targeting of antigens/immunostimulants to specialized APCs responsible for maintaining the delicate balance between immunity and tolerance of $CD8^+$ T cells.[16,21] In this study, we found that the cervical cancer antigen, E7, conjugated with U, was mainly taken up by myeloid cells and extensively internalized by resident $CD8^+$ DCs and migratory $CD103^+$ DCs (Figure 1D). Because murine cDC1 subtypes, including $CD8^+$ DC and $CD103^+$ DCs, are known to uptake exogenous antigens

Figure 4. Induction of antigen-specific T cell response and tumor regression by the UCVs in a subcutaneous cervical cancer and lymphoma model
(A) C57BL/6 mice were subcutaneously inoculated with 1×10^5 TC-1 cells. On days 6 and 13, mice were subcutaneously injected with 5 nmol of E, E+U, and EUCV, and tumor volumes were measured 3 times a week using a caliper (n = 4 per group). (B–D) Tumors were excised on day 20, and TILs were isolated from tumor tissues. The frequencies of (B) $CD8^+$ T cells and (C) E7-specific $CD8^+$ T cells were assessed using flow cytometry. (D) The expression levels of genes related to $CD8^+$ T cell activation, including Perforin, Granzyme B, FasL, Granulysin, IFN-γ, CXCL-9, CD69, and Tbx21, were analyzed using qRT-PCR. (E) C57BL/6 mice were subcutaneously inoculated with 1×10^6 E.G7-OVA cells. On days 3 and 10, mice were subcutaneously injected with 5 nmol of O, O+U, and OUCV, and tumor volumes were measured 2–3 times a week using a caliper (n = 4–5 per group). (F–H) Tumors were excised on day 17, and TILs were isolated from tumor tissue. The frequencies of (F) $CD8^+$ T cells and (G) OVA-specific $CD8^+$ T cells were assessed using flow cytometry. (H) The expression levels of inflammatory cytokines and genes related to cell activation were analyzed using qRT-PCR. Data are representative of three independent experiments, and the results are presented as the mean ± SEM. Statistical significance was analyzed using Student's t test (*$p < 0.05$, **$p < 0.01$, ***$p < 0.001$). S.C., subcutaneous; TIL, tumor-infiltrated lymphocyte.

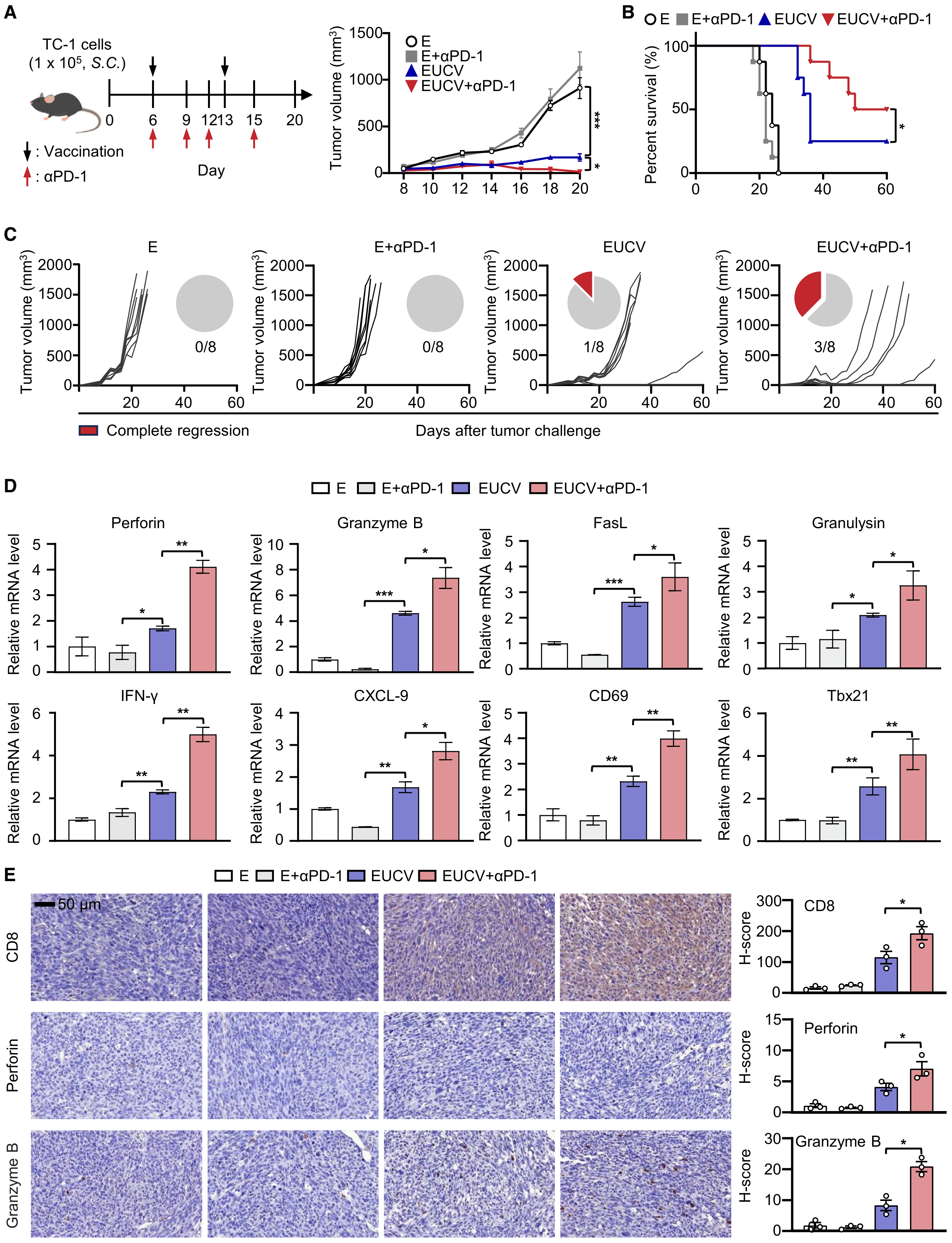

(legend on next page)

and cross-present them to CD8+ T cells, we hypothesized that UCVs may also facilitate the priming of antigen-specific CD8+ T cells.[39] Furthermore, as CD103+ DCs and CD8+ DCs can share antigens through synapses, targeting both cell types with UCVs is expected to enhance the interaction of these cells with CD8+ T cells.[40] Additional findings demonstrated that UCVs induced BMDCs to express inflammatory cytokines (IL-12p70 or IL-6) and costimulatory molecules (CD80 or CD86) while increasing the recruitment of DC subsets *in vivo*. Because the recruitment and activation of DCs, alongside antigen internalization, are required for CD8+ T cell priming, these results support the hypothesis that UCVs can act as a mediator facilitating cDC1-CD8+ T cell interactions.[41] Intriguingly, murine CD8+ DCs, the specific target cells of UCV, share high conservation with human CD141+ DCs, which play a pivotal role in cross-priming CD8+ T cells in the human immune system.[42] CD141+ DCs, a subtype of human cDC1s, have clinical relevance to the induction of anticancer immunity and prognosis of patients with cancer, exhibiting substantial expression of TLR2 and 6.[43–45] As revealed by the results, EUCV exhibited greater internalization in CD141+ DCs than in CD1c+ DCs, displaying consistency with the observed specific internalization of UCV in the mouse cDC1 subset (Figure S10). Moreover, innate immune response analysis also indicated a stronger response in CD141+ DCs, suggesting that internalization and immune response induction by UCV is potentially applicable not only to mouse systems but also to human systems. Consequently, the profile of UCV target cells shown in the murine model suggests promise for potential clinical applications.

To effectively target antigens to DCs, the antigens should be highly accumulated in the dLNs. However, SLPs with antigenic properties often disperse widely throughout the body, lowering the chance of reaching dLNs. This raises biodistribution challenges and can result in protease degradation and undesired immune tolerance.[14,46] To address these concerns, we explored the potential of U conjugation to enhance the pharmacokinetic properties of the conjugated SLPs.[13] The covalent linkage of U to SLPs decelerates the systemic distribution of the conjugated antigens. Biodistribution analysis showed that UCVs exhibited a propensity to accumulate in the nearby inguinal dLNs, whereas the antigens alone dispersed to distant LNs (Figure 1B). Importantly, the localized vaccine was taken up by migratory CD103+ DCs and resident CD8+ DCs. This suggests that the administered vaccine either directly reaches the dLNs and is absorbed by resident APCs or is taken up by migratory APCs at the injection site and then delivered to the dLNs. Compared with a mixture of SLPs and U, the conjugated vaccine displayed superior localization in the inguinal dLNs and demonstrated enhanced antigen-presentation capabilities (Figure S9C). The similarity in CD80 expression, a crucial costimulatory molecule, underscores the importance of direct antigen delivery in facilitating cross-presentation. Notably, UCVs outperformed other TLR agonist mixtures by robustly inducing the production of IFN-γ, along with enhanced priming of E7-specific CD8+ T cells *in vivo* (Figures 3A–3D). Furthermore, our experiments with BMDCs from TLR2 WT and KO mice highlighted the necessity for coordinated target cell activation and antigen delivery to sustain CD8+ T cell responses (Figure 3E).

As expected, the DC depletion experiment revealed that the antigen-specific immune response of UCV was abolished, underscoring the importance of activation and antigen delivery to target cells (Figure S12). However, because clodronate depletes not only DCs but also macrophages, specific selection is challenging. While macrophages can process external antigens and exhibit cross-presentation, their efficiency in these functions is generally less robust than that of DCs.[47] Therefore, even though macrophages may play a role in the clodronate-mediated suppression of the immune response in UCV treatments, DCs are still the primary target cells due to their superior capacity for antigen presentation and activation of T cells. To induce DC-specific deficiency, the CD11c-DTR transgenic mouse system should be used; however, owing to the limitations of experimental techniques, a broader range of deficiency using clodronate has been adopted. Ultimately, UCV has the potential to effectively induce antigen-specific CD8+ T cell responses by leveraging the TLR2/6 affinity conferred by U, thus restricting systemic exposure of antigens, and promoting lymphatic localization and efficient antigen delivery based on its increased molecular size.

Our findings indicate that UCV exploited the TLR2/6 and clathrin-dynamin-mediated endocytosis pathways for internalization. This observation aligns with previous research, where the TLR2 ligand Pam2IDG was also found to enter cells through clathrin-mediated endocytosis, with TLR2 playing a central role.[28] Moreover, cells with a relatively high uptake of UCV also displayed elevated TLR2/6 expression. Exogenous antigens can be absorbed by cells via processes such as phagocytosis, pinocytosis, and receptor-mediated endocytosis, with the latter significantly enhancing antigen uptake and cross-presentation.[48] Thus, the increased antigen uptake specifically observed in DCs with high TLR2/6 expression, especially the aforementioned CD8+, CD103+, and CD64+ DCs, suggests that this

Figure 5. Synergistic effect of the combination of the UCV and anti-PD-1 blockade

(A and B) C57BL/6 mice were subcutaneously inoculated with 1×10^5 TC-1 cells. On days 6 and 13, mice were subcutaneously injected with 5 nmol of E and EUCV at the opposite dorsal side. Anti-PD-1 blockade was intraperitoneally injected on days 6, 9, 12, and 15 to check the synergistic effect. (A) Tumor volumes were measured 3 times a week using a caliper (n = 8 per group), and (B) the percentage of mouse survival was determined. (C) Individual tumor growth and survival profile of TC-1 tumor-bearing mice treated with the indicated vaccine and/or anti-PD-1 blockade for 60 days (n = 8 per group). (D) Gene expression was assessed via qRT-PCR using resected tumor tissue. (E) Tumor tissue slides were stained with anti-CD8, anti-perforin, and anti-granzyme B antibodies to identify T cells, followed by immunohistochemistry. Scale bar, 50 μm. Data are representative of three independent experiments, and the results are presented as the mean ± SEM. Statistical significance was analyzed using Student's t test, and survival days were analyzed using the Kaplan-Meier method with the log rank (Mantel-Cox) test (*p < 0.05, **p < 0.01, ***p < 0.001). PD-1, programmed cell death protein 1; CXCL-9, chemokine (C-X-C motif) ligand 9.

is potentially advantageous in eliciting antigen-specific immune responses. In contrast, another study demonstrated that a Pam3Csk4-conjugated lipopeptide engaged APCs via TLR2 activation, nevertheless, the internalization process occurred independently of TLR2 and necessitated both clathrin- and caveolae-mediated endocytosis.[49] Together, these investigations highlight the significant role of TLR2 in endocytosis and activation, with the specific mechanism depending on the physicochemical properties of the ligand. MHC class I cross-presentation, facilitated by TLR2 and clathrin-mediated antigen uptake, encompasses multiple pathways. Typically, internalized antigens are processed in a common route: they are transported to the cytoplasm, degraded by the proteasome, and presented via MHC class I in the endoplasmic reticulum.[50] Our study revealed that UCVs partially rely on CS, contributing to the vacuolar pathway, an alternative, TAP-independent route. The reduction in antigen presentation with CS indicates its partial role (Figure 2F). Clathrin-dynamin-mediated endocytosis is closely tied to the endocytic recycling compartment (ERC), a source of MHC class I for the vacuolar pathway. Notably, the vesicles containing ERC-derived MHC class I are optimized in the vacuolar pathway through Myd88-dependent TLR signaling, eventually fusing with phagosomes carrying TLR ligands.[51] The vacuolar pathway can counteract immune evasion strategies used by oncogenic viruses or the TME, which hinder the TAP function from evading immune surveillance.[52–54] Consequently, the processes of clathrin-dynamin-mediated uptake and TLR2/6-driven cellular activation support the effectiveness of UCVs in efficiently inducing antigen-specific cellular immune responses.

SLP antigens offer the advantages of enhancing the antitumor immune response, including efficient processing and preventing immunological tolerance, as demonstrated in clinical studies.[55,56] In contrast to synthetic short peptides, which may lead to suboptimal presentation or immune tolerance on non-professional APCs, SLP antigens have the potential to efficiently prime $CD8^+$ T cells through cross-presentation, especially compared with whole protein antigens.[57] While research into therapeutic cancer vaccines explores various platforms, including nucleic acid vaccines, such as DNA and RNA, these systems often present challenges related to immunogenicity, potential integration into the host genome, leading to gene dysfunction or inactivation, and the complexity of conservation processes.[58,59] This work suggests that the covalent coupling of natural immune stimulators, such as U-conjugated SLP antigens, can be a means of overcoming these challenges and improving pharmacokinetic limitations.

Over 70% of patients with cervical cancer are infected with HPV, particularly HPV-16 as the major subtype.[60] Prophylactic vaccines primarily target HPV viral capsid proteins L1 and L2, whereas therapeutic vaccines target major oncoproteins E6 and E7. Among them, E7 is considered a vaccine antigen due to its high carcinogenicity prevalence, low somatic mutation frequency, and relatively high human leukocyte antigen (HLA)-type coverage in patients.[61–64] In our study, we selected highly immunogenic candidates with predicted CD8 epitopes within the E7 or E6 oncoproteins, as identified in previous research.[65] Among the various SLPs derived from E7 and E6, the data revealed that $E7_{43–62}$ SLP elicited a robust antigen-specific immune response. Consequently, we developed UCV using $E7_{43–62}$ SLP as the antigen. Notably, both *in vivo* results using U and prediction scores generated using the NetCTL server (http://www.cbs.dtu.dk/services/NetCTL) consistently identified $E7_{43–62}$ as the most promising SLP, indicating the alignment between experimental results and the prediction model.[19] Notably, the $E7_{11–19}$ epitope, predominantly presented on HLA molecules, can be inhibited by the $E7_{49–57}$ epitope.[66] This suggests the need for a unique strategy when combining different antigens, as multiple antigens potentially inhibit subsequent immune responses. While accurate assessment in this context may pose challenges, the results indicated an increase in the $E7_{11–19}$-specific $CD8^+$ T cell response due to U, warranting further investigation.

In the clinical context of cervical cancer, PD-1 ICIs have shown promise in countering the immunosuppressive TME. Nevertheless, low response rates in some patients are linked to insufficient T cell activation and limited tumor infiltration.[67–69] This study explored combination therapy involving UCV and a PD-1 ICI in a mouse tumor model. The results demonstrated effective tumor suppression and improved survival rates, coupled with a notable increase in activated $CD8^+$ T cell infiltration, resulting in complete tumor suppression in some cases (Figures 5A–5C).[70,71] Blocking the PD-1 pathway alone can face resistance in the absence of robust T cell priming, suggesting that T cell priming is a crucial step to unlocking the potential of PD-1 ICIs.[35] These findings highlight the synergistic potential of the conjugated vaccine and PD-1 ICIs, offering a combined therapeutic approach to enhance response rates and overcome immune barriers in cervical cancer treatment.

In conclusion, our study introduces a novel immunotherapy approach employing a conjugated vaccine incorporating U, an endogenous immune-activating domain obtained from human CARS1. Our results highlight the enhanced anticancer efficacy of this conjugated, attributed to its advantageous biodistribution and specific antigen delivery to functional cells, compared with that of conventional peptide vaccines. Notably, the conjugated vaccine exhibited a synergistic effect when used in combination with PD-1 ICIs, and no toxicity was observed when administered at high doses. This research underscores the potential of U-based conjugate vaccines as promising tools for cancer immunotherapies with the capacity to enhance the effectiveness of established treatments.

MATERIALS AND METHODS

Cell lines

THP-1 (ATCC, RRID: CVCL_0006), TC-1 (from Dr. C.Y. Kang/Seoul National University, RRID: CVCL_4699), E.G7-OVA (ATCC, RRID: CVCL_3505), DC2.4 (Millipore, RRID: CVCL_J409), A20 (ATCC, RRID: CVCL_1940), and B3Z (from Dr. S.B. Kim/Sahmyook University, from RRID: CVCL_6277) cells were cultured in RPMI-1640 medium (HyClone, no. SH30255.01) supplemented with 10% heat-inactivated FBS (HyClone, no. SV30207.02) and 1%

penicillin-streptomycin (HyClone, no. SV30010). EL4 (ATCC, RRID: CVCL_0255) and Raw 264.7 (ATCC, RRID: CVCL_0493) were cultured in DMEM (HyClone, no. SH30243.01) supplemented with 10% heat-inactivated FBS and 1% penicillin-streptomycin. THP-1, DC2.4, A20, and B3Z cell culture medium were added with 50 μM β-mercaptoethanol (Thermo Fisher Scientific, no. 21985-023). To differentiate THP-1 cells, PMA (Sigma-Aldrich, no. P8139-1MG) was diluted with 50 ng/mL into a culture medium and incubated for 24 h. After a day, the differentiation medium was exchanged with the culture medium and incubated for 24 h. All cell lines were cultured for a limited number of passages (<10 passages) and maintained at 37°C and 5% CO_2 in an incubator. The cell lines were tested for *Mycoplasma* contamination by PCR method (Bionicsro).

Animal

$TLR2^{-/-}$ mice were kindly provided by Dr. Myung Hee Kim (Korea Research Institute of Bioscience and Biotechnology, South Korea) and Dr. Eun-Kyeong Jo (Chungnam National University, South Korea). Six-week-old female C57BL/6 mice were purchased from DooYeolBiotech. All animals were maintained in the pathogen-free authorized facility at Yonsei University, and all experiments were performed under the approval of the Institutional Animal Care and Use Committee (IACUC-202212-1578-01).

Peptide

All SLP peptide antigens were predicted from HPV16 E6 (P03126) and E7 (P03129) sequences using the prediction systems of Immune Epitope Database & Tools (https://www.iedb.org/) or by manual screening. SLP peptide antigens and epitope peptides were purchased from GL Biochem (Shanghai, China) and reconstituted in 4 mg/mL in distilled water. The amino acid sequence information for all antigens used is listed in Table S3.

Antibodies

For flow cytometry analysis, antibodies to CD11c (clone N418, RRID: AB_313778), CD3 (clone 17A2, RRID: AB_312661), CD8 (clone 53–6.7, RRID: AB_312750), CD45 (clone 30-F11, RRID: AB_312971), CD80 (clone 16-10A1, RRID: AB_313126), CD86 (clone GL-1, RRID: AB_313148), CD40 (clone 3/23, RRID: AB_1134090), CD45R/B220 (clone RA3-6B2, RRID: AB_893355), CD11b (clone M1/70, RRID: AB_2129374), CD19 (clone 6D5, RRID: AB_313643), F4/80 (clone BM8, RRID: AB_893493), CD103 (clone 2E7, RRID: AB_535948), CD172 (clone P84, RRID: AB_2563549), CD64 (clone X54.5/7.1, RRID: AB_10613497), XCR1 (clone ZET, RRID: AB_2564363), HLA-DR (clone L243, RRID: AB_893574), CD11c (clone Bu15, RRID: AB_1236439), CD1c (clone L161, RRID: AB_10644008), CD141 (clone M80, RRID: AB_10899578), CD123 (clone 6H6, RRID: AB_493576), CD80 (clone 2D10, RRID: AB_314501), CD83 (clone HB15e, RRID: AB_314514), CD86 (clone BU63, RRID: AB_2721573), Perforin (clone S16009A, RRID: AB_2721638), Granzyme B (clone QA16A02, RRID: AB_2687031), IFN-γ (clone XMG1.2, RRID: AB_315402), TNF-α (clone MP6-XT22, RRID: AB_315426), FasL (clone MFL3, RRID: AB_313278), and $OVA_{257–264}$ (SIINFEKL) peptide bound to H-2Kb monoclonal antibody (clone 25-D1.16, RRID: AB_10895905) were purchased from BioLegend, and CD69 (clone H1.2F3, RRID: AB_396675) was purchased from BD, and CD207 (Langerin, clone eBioL31, RRID: AB_763452), CD282 (TLR2, clone 6C2, RRID: AB_465440), IgG2b kappa isotype control (clone eB149/10H5, RRID: AB_470004), and IgG2a kappa isotype control (clone eBR2a, RRID: AB_493963) were purchased from Thermo Fisher, and CD286 (TLR6, clone 148601, RRID: AB_2256201) was purchased from R&D Systems. For immunohistochemistry staining, antibodies to Granzyme B (no. ab53097, RRID: AB_2114427) and Perforin (no. ab16074, RRID: AB_302236) were purchased from Abcam, and CD8 (no. 14-0081-82, RRID: AB_467087) was purchased from Thermo Fisher. For western blot detection, rabbit polyclonal anti-His (Santa Cruz, clone H-3, no. sc-8036, RRID: AB_627727) and goat anti-mouse IgG (H+L), HRP (Thermo Fisher Scientific, no. 31430, RRID: AB_228307) were purchased.

BMDC differentiation

Bone marrow was harvested from femurs and tibias of C57BL/6 mice. Red blood cells were lysed with Pharm Lyse lysing solution (BD, no. 555899), and the remaining cells were washed using DPBS (HyClone, no. SH30378.02), followed by centrifugation to the collection. Bone marrow cells were resuspended at a concentration of 1×10^6 cells/mL in RPMI-1640 medium supplemented with 10% FBS, 1% streptomycin-penicillin, and 20 ng/mL GM-CSF (R&D Systems, no. 415-ML-005). On day 3, add an additional medium of the same supplemented as described above. BMDCs were harvested on day 6 and seeded at 3×10^5 cells/mL in 24-well plates for stimulation.

DNA construct

HPV16 $E7_{43–62}$ SLP (GQAEPDRAHYNIVTFCCKCD) and $OVA_{247–264EAAAAK}$ SLP (DEVSGLEQLESIINFEKLAAAAK) sequences were synthesized (GENEWIZ) and cloned into a pET28a expression vector (Bionicsro). The U sequence was inserted at the back of the SLP sequence, and the space was filled by a rigid ($EAAAK_3$) or flexible ($GSSSS_3$) peptide linker sequence.[20] The amino acid and DNA sequences of DNA construct are listed in Tables S1 and S2.

Conjugated vaccine expression and purification

All constructs were transformed with E. coli BL21-CodonPlus (DE3)-RIPL competent cells (Agilent, no. 230280), and a single colony was overnight inoculated into 3 mL LB medium with 50 μg/mL kanamycin at 37°C. After incubation, 1 mL inoculated LB medium was transferred to fresh 1 L LB medium, and 0.5 mM of IPTG to induce protein expression when fresh LB medium OD_{600nm} reached 0.4–0.5. After inducing at 4°C for 16 h, the cells were harvested and resuspended in lysis buffer (50 mM Tris [pH 7.5], 300 mM NaCl, 5% glycerol). Suspended cells were sonicated in ice and centrifuged at 5,000 × *g* for 30 min at 4°C. The soluble fraction passed through a 0.45-μm filter was bound twice to the Ni-NTA resin (Thermo Fisher Scientific, no. 88221). After fraction loading, the column was washed with washing buffer (50 mM Tris [pH 7.5], 300 mM NaCl, 5% glycerol, and 15 imidazole) and eluted with elution buffer (50 mM Tris

[pH 7.5], 300 mM NaCl, 5% glycerol, and 300 mM imidazole). Eluted proteins were dialyzed using dialysis tubing (Thermo Fisher Scientific, no. 68100) with dialysis buffer (300 mM NaCl and 15% glycerol containing PBS) for 16 h at 4°C. For endotoxin removal, proteins were mixed with endotoxin buffer containing 2% Triton X-114 (Sigma-Aldrich, no. X114-500ML) based on dialysis buffer. As a result of confirmation using the LAL assay kit (Thermo Fisher Scientific, no. 88282) to measure the remaining LPS, it was found that about 0.04 EU/mg remained.

CD spectroscopy

CD spectroscopy was used to demonstrate the thermal stability of conjugated vaccines, and far-UV CD spectra were recorded on a Chirascan spectrometer (Applied Photophysics) using a cell with a path length of 1 mm. To compare the structural conformation of the native or boiled form of conjugated vaccines, indicated samples were analyzed to far-UV CD measurements at 20°C. CD spectra were obtained over the wavelength range of 190–260 nm with 1.0 nm bandwidth and modified as molar ellipticity, [θ] (degree cm^2 $dmol^{-1}$).

Western blot

Isolated protein concentration was calculated using a BCA protein assay kit (Thermo Fisher Scientific, no. 23225) and boiled in 1× reducing sample buffer for 10 min at 95°C. Reduced protein samples (1 μg) were run on an SDS-PAGE gel and blotted on an Immobilon-P PVDF membrane (Merck, no. IPVH00010) using the Trans-blot semi-dry transfer cell (Bio-Rad). After blocking with 5% skim milk (Difco, no. 232100) for 1 h at room temperature, membranes were incubated overnight at 4°C with His-probe (1:1,000) followed by detection with a goat anti-mouse secondary antibody, HRP (1:10,000). The target band was visualized using the Absignal detection reagent (Abclon, no. ABC-3001).

SLP screening

Seven-week-old C57BL/6 mice were immunized subcutaneously in the right back twice at 1-week intervals using SLPs (HPV16 $E6_{23-42}$, $E6_{43-62}$, $E6_{123-143}$, E_{75-25}, $E7_{43-62}$, and $E7_{76-95}$; 20 μg per SLP) with U (100 μg). After 7 days at final immunization, cells were isolated from the spleen and seeded in the ELISpot well in triplicates (5×10^5 cells per well) with 10 μg/mL of re-stimulatory 8–9 mer epitope peptides (HPV16 $E6_{29-37}$, $E6_{49-57}$, $E6_{129-138}$, $E7_{11-19}$, $E7_{49-57}$, and $E7_{82-90}$), respectively. Antigen-specific immune responses were detected by AID EliSpot Reader (AID, no. ELR08).

In vitro myeloid cell stimulation

Differentiated THP-1, BMDC (3×10^5 cells per well) were seeded, and after 24 h starved with serum-free-medium for 1 h. Each sample was treated and reacted for 4 or 16 h. To measure the inflammatory cytokine level, the culture medium was harvested, centrifuged at $500 \times g$ for 10 min at 4°C, and the supernatant was separated. The expression of inflammatory cytokines was measured using hTNF-α (BD, no. 555212), IL-12p70 (BD, no. 555256), IL-12p40 (BD, no. 555165), and IL-6 (BD, no. 555240) ELISA kits according to the manufacturer's instructions. To investigate the expression of costimulatory molecules on the cell surface, cells were harvested using dissociation buffer (5 mM EDTA in DPBS), washed, and stained with 1 μg/mL antibody, followed by flow cytometry analysis. Antibodies: anti-CD11c (BioLegend, no. 117309), anti-CD40 (BioLegend, no. 117309), anti-CD80 (BioLegend, no. 117309), and anti-CD86 (BioLegend, no. 117309).

HEK-Blue SEAP assay

HEK-Blue hTLR2/6 (Invivogen, no. hkb-htlr26), hTLR2/1 (Invivogen, no. hkb-htlr21), and hTLR4 (Invivogen, no. hkb-htlr4) cells were cultured in DMEM (HyClone, no. SH30243.01) supplemented with 10% heat-inactivated FBS, 1% penicillin-streptomycin, and 100 μg/mL normocin (Invivogen, no. ant-nr-1), with added HEK-Blue Selection (Invivogen, no. hb-sel) added in medium after the cells were passaged twice. For the SEAP assay, HEK-Blue cells were harvested and resuspended to 2.8×10^5 cells/mL in a culture medium. Different concentrations of U, conjugated vaccine, Pam2CSK4 (Invivogen, no. tlrl-pm2s-1), Pam3CSK4 (Invivogen, no. tlrl-pms), and LPS (Invivogen, no. tlrl-3pelps) were added in 96-well plates at 20 μL, and 80 μL of the cell suspension was seeded (5×10^4 cells/well). The plates were then incubated for 24 h at 37°C, and 5% CO_2 and the supernatant was collected. After removing the remaining cells by centrifugation, 20 μL of supernatant was transferred into a 96-well plate with 180 μL QUNATI-Blue solution (Invivogen, no. rep-pbs). All plates were incubated at 37°C, 5% CO_2 for 3–4 h, and absorbance was measured at 620 nm with a microplate reader (TECAN, no. 30087502).

Immunofluorescence

5-FAM-$E7_{43-62}$ peptide was synthesized from Anygen and recombinant proteins EUCV were labeled with NHS-5/6-FAM (Thermo Fisher Scientific, no. 46410) according to the manufacturer's instructions. After protein labeling, the concentration of protein was calculated using A493. BMDC cells (3×10^5) were seeded on the coverslip and placed in the 24-well plate. After 24 h, the cells were starved with serum-free medium for 1 h and treated with 100 nM FAM-E-, FAM-E+U-, and FAM-labeled EUCV for 6 or 24 h. After the sample treatment, the medium was removed from the well, replaced with a medium containing 100 nM Lysotracker (Thermo Fisher Scientific, no. L7528), and cultured for 1 h. Cells were washed twice with cold PBS and fixed with 4% paraformaldehyde (Biosesang, no. PC2031) for 10 min. After washing twice with cold PBS, coverslips were incubated with CAS-Block buffer (Thermo Fisher Scientific, no. 008120) for 10 min, followed by washing 2 times with cold PBS and incubation with Hoechst 33342 (1:500) for 10 min in the dark. After washing twice with PBS, coverslips were mounted on a slide glass and dried for 2 h in a dark place. The slides were observed using a confocal microscope (Nikon, A1Rsi).

In vitro antigen uptake and presentation

To investigate time-dependent uptake of antigens, isolated BMDC cells (3×10^5) were seeded in the 24-well plate and incubated with FAM-E-, FAM-E+U-, or FAM-labeled EUCV at 37°C, 5% CO_2 for 6 or 24 h. BMDCs were harvested and stained with 1 μg/mL of

APC anti-CD11c antibody for 30 min at 4°C, followed by antigen uptake was analyzed by flow cytometry. To examine TLR2-specific antigen uptake and presentation, BMDCs were isolated from $TLR2^{WT}$ and $TLR2^{-/-}$ mice as described above. BMDC cells (3×10^5) were seeded in the 24-well plate. BMDCs were incubated with 100 nM FAM-O-, FAM-O+U-, and FAM-labeled OUCV at 37°C, 5% CO_2 for 24 h. BMDCs were harvested and stained with 1 μg/mL of APC anti-CD11c antibody for 30 min at 4°C, followed by antigen uptake was analyzed by flow cytometry. For antigen presentation, BMDC cells (3×10^5) were seeded in the 24-well plate and incubated with 100 nM O, O+U, and OUCV at 37°C, 5% CO_2 for 24 h. BMDCs were harvested and stained with 1 μg/mL of APC anti-CD11c and PE anti-mouse H-$2K^b$ bound to SIINFEKL antibody for 30 min at 4°C, followed by antigen presentation, and analyzed by flow cytometry. To investigate the mechanism of uptake and presentation, the BMDCs were pretreated with 20 or 40 μM CM (Sigma-Aldrich, no. C8138), 20 or 40 μM dynasore (Sigma, no. 324410), 1 or 2 μM cathepsin S inhibitor (MCE, no. LY 3000328), 1 or 2 μM methyl-β-cyclodextrin (Sigma-Aldrich, no. C4555), or 1 or 2 mM amiloride (Merck, no. A7410) 1 mg/mL for 30 min. After pretreatment, labeled or native samples were treated in the wells, followed by antigen uptake or presentation analyzed by flow cytometry as described above.

TLR2 and TLR6 expression test

For the *in vitro* confirmation of TLR2/6 expression, we used mouse cancer cell lines E.G7-OVA (lymphoma) and TC-1 (lung cancers), along with immune cell lines EL4 (T cells), A20 (B cells), Raw 264.7 (macrophages), and DC2.4 (dendritic cells). The cells were stained with anti-TLR2 antibody, anti-TLR6 antibody, IgG2b kappa isotype control (TLR2 isotype control), and IgG2a kappa isotype control (TLR6 isotype control) and analyzed by flow cytometry. The expression level was normalized using the respective isotype controls for each TLR antibody. For the analysis of TLR 2/6 expression in immune cells *in vivo*, cells were isolated from LNs of naive mice. The various DC subtypes were categorized into two groups as follows: group 1 consists of T cells ($CD45^+$ and $CD3^+$), B cells ($CD3^-$ and $CD19^+$), macrophages ($CD11b^+$ and $F4/80^+$), and DCs ($CD11b^-$ and $CD11c^+$). Group 2 includes $CD8^+$ DCs ($CD11c^+$, $XCR1^+$, and $CD8^+$), $CD103^+$ DCs ($CD11c^+$, $XCR1^+$, and $CD103^+$), $CD172^+$ DCs ($CD11c^+$ and $CD172^+$), $B220^+$ DCs ($CD11c^+$ and $B220^+$), $Langerin^+$ cells ($CD11c^+$ and $Langerin^+$), and $CD64^+$ DCs ($CD11c^+$ and $CD64^+$). The expression was measured by flow cytometry after staining the cells with anti-TLR2 antibody, anti-TLR6 antibody, IgG2b kappa isotype control (TLR2 isotype control), and IgG2a kappa isotype control (TLR6 isotype control). The expression level was normalized using the appropriate isotype controls for each TLR antibody.

Human DC isolation and stimulation

Pan-DCs were isolated from human PBMCs (Lonza, no. CC-2703) using the Pan-DC Enrichment Kit (Miltenyi Biotec, no. 130-100-777), according to the Miltenyi Biotec protocol. By using the kit, non-DCs were retained within a magnetic field in the LS column, and the flowthrough containing pan-DCs was collected. Subsequently, the LS column was removed from the separator and non-DCs were collected using a plunger. The separated pan-DCs and mon-DCs were resuspended in RPMI-1640 medium supplemented with 50 μM β-mercaptoethanol, 1× non-essential amino acids (HyClone, no. SH30853.01), 1 mM sodium pyruvate (Thermo Fisher Scientific, no. 11360070), and 10% human normal serum (Merck, no. S1-100ML). Cells (1×10^5) were seeded in a 24-well plate and treated with LPS, E, U, E+U, and EUCV. After incubation at 37°C with 5% CO_2 for 24 h, the supernatant was collected for TNF-α quantification using an ELISA kit (BD). Then, cells were collected and subtyped using anti-HLA-DR, anti-CD11c, anti-CD1c, anti-CD141, and anti-CD123 antibodies, and the expression levels of costimulatory molecules were analyzed using anti-CD80, anti-CD83, and anti-CD86 antibodies through flow cytometry.

Flow cytometry

Flow cytometry analysis was performed using primary cells or cell line suspensions. Cells were first washed with PBS and incubated with blocking buffer (1% BSA in PBS) at 4°C for 20 min. After incubation in the blocking buffer, cells were stained for surface markers with antibodies diluted in the blocking buffer at 4°C for 30 min. After washing in the blocking buffer, cells were resuspended with the blocking buffer for detection. Antibodies used: anti-CD40, anti-CD80, anti-CD86, anti-CD11c, anti-CD3, anti-CD8, anti-CD45, anti-B220, anti-CD69, and anti-mouse H-$2K^b$ bound to SIINFEKL, all from BioLegend. For intracellular staining, cells were fixed and permeabilized using Cytofix/Cytoperm solution (BD, no. 554722) at 4°C for 20 min. After washing in Perm/Wash buffer (BD, no. 554723), cells were stained with intracellular antibodies diluted in Perm/Wash buffer at 4°C for 30 min. After washing in Perm/Wash buffer, cells were resuspended with blocking buffer for detection. Antibodies used: anti-IFN-γ and anti-TNF-α from BioLegend. Cells were acquired on an Accuri C6 plus flow cytometer (BD, no. 663931) and analyzed using FlowJo v.7 (FlowJo LLC).

Pharmacokinetics

Seven-week-old mice were intravenously or subcutaneously injected with 5 nmol labeled SLP or protein. At the indicated time point, blood was collected using ophthalmic bleeding into heparin-coated micro hematocrit capillary tubes (Fisherbrand, no. 22-362574). After being transferred blood into a microtube, plasma was isolated from blood by centrifugation at 4°C for 10 min at 1,000 × *g* to eliminate the cells, and then centrifugation at 4°C for 15 min 2,000 × *g* for removed platelets. The plasma was diluted 1:20 into PBS and measured on Infinite 200 PRO (TECAN) against a standard curve of analytes diluted into PBS (Ex/Em: 494/518 nm). All plasma was normalized to the maximum fluorescence detected in the individual injection sample and plotted as percent injection dose (% ID) versus time. The half-life was calculated by fitting the intravenous plasma curve using $C = C_0 \times e^{-kt}$. The subcutaneous curve was fit to $C = [F \times k_a \times C_0 / V_D(k_a - k)] \times (e^{-k \times t} - e^{-k_a \times t})$, where C is the plasma concentration of indicated times, C_0 is the initial serum concentration, k is the elimination rate constant, F is the systemic absorbance, k_a is the absorption rate constant, V_D is the volume of

distribution.[72] The curve graphs were represented using nonlinear regression on GraphPad Prism.

Biodistribution

Seven-week-old C57BL/6 mice were subcutaneously injected with 5 nmol FAM-E-, FAM-E+U-, or FAM-labeled EUCV, respectively. After 0.5, 2, 6, or 24 h, organs were collected and washed with PBS to remove any remaining blood. For biodistribution analysis, images were obtained using an In Vivo Imaging System (IVIS) Lumina II imaging systems (PerkinElmer; excitation 494 nm, emission 518 nm) and analyzed by Living Image software.

In vivo antigen uptake, presentation, and activation of DCs

To assess the effects of antigen uptake and cell activation *in vivo*, 7-week-old C57BL/6 mice were injected subcutaneously with 5 nmol FAM-E-, FAM-E+U-, or FAM-labeled EUCV, respectively. After 2 or 24 h, cells were harvested from the inguinal draining LN, and the remaining red blood cells lysed using Pharm Lyse lysing solution. For analysis of each immune cell, cells were stained with 1 μg/mL of anti-B220, anti-CD3, anti-F4/80, and anti-CD11c antibodies, and classified to identify T cells ($CD45^+$ and $CD3^+$), B cells ($CD3^-$ and $CD19^+$), macrophages ($CD11b^+$ and $F4/80^+$), and DCs ($CD11b^-$ and $CD11c^+$). For DC subtype analysis, cells were stained with 1 μg/mL of anti-CD11c, anti-B220, and anti-CD8 antibodies, and classified to identify $CD8^+$ DCs ($CD11c^+$, $XCR1^+$, and $CD8^+$), $CD103^+$ DCs ($CD11c^+$, $XCR1^+$, and $CD103^+$), $CD172^+$ DCs ($CD11c^+$ and CD172+), $B220^+$ DCs ($CD11c^+$ and $B220^+$), $Langerin^+$ cells ($CD11c^+$ and $Langerin^+$), and $CD64^+$ DCs ($CD11c^+$ and $CD64^+$). FITC signals were detected for antigen uptake and anti-CD80 antibody was used to measure activation level. For antigen presentation, mice were injected subcutaneously with O, O+U, and OUCV, respectively. Cells were prepared as described above, and the anti-mouse H-$2K^b$ bound to SIINFEKL antibody used to analyze antigen presentation through flow cytometry.

In vitro cross-presentation

BMDCs (from $TLR2^{WT}$ or $TLR2^{-/-}$) were seeded in 96-well plates at 1×10^5 cells/well and pulsed with 100 nM of O, O+U, and OUCV for 16 h. After washing with PBS, B3Z cells were added to the well at 2×10^5 cells/well. After co-culture, cells were centrifuged at room temperature at 500 × *g* for 2 min, followed by washing with PBS. Cells were incubated with 100 μL of CPRG (Merck, no. 10884308001) lysis solution (0.5% NP-40 and 91 μg/mL CPRG powder containing PBS) at 37°C for 4 h and the absorbance at 570 with 650 nm measured as the reference, to estimate the activation of B3Z cells. For T cell activation marker and cytokine analysis, co-cultured cells were harvested, and cells or supernatant were separated by centrifugation. The cells were stained with 1 μg/mL anti-CD45, anti-CD3, anti-CD8, anti-CD69, anti-Perforin, anti-Granzyme B, anti-IFN-γ, and anti-FasL antibodies, followed by flow cytometry analysis. Cytokine levels were measured using the IL-2 (BD, no. 555148) ELISA kit using the supernatant according to the manufacturer's instructions.

In vivo $CD8^+$ T cell priming

Seven-week-old C57BL/6 mice were subcutaneously injected in the right flank twice at 1-week intervals with 5 nmol of Flagellin (Invivogen, no. vac-fla), MPLA (Invivogen, no. vac-mpls), poly(I:C) (Invivogen, no. vac-pic), U plus E, E alone, or EUCV. After 7 days at final injection, splenocytes and inguinal draining LN cells were harvested, and the red blood cells lysed using Pharm Lyse lysing solution. For ELISpot, cells (5×10^5 cells per well) were seeded in an ELISpot plate and proceeded to mouse IFN-γ ELISpot assay (Mabtech, no. 3321-4APT) according to the manufacturer's instructions. To analyze antigen-specific $CD8^+$ T cell frequency, cells were stained with 1 μg/mL of anti-CD45, anti-CD3, anti-CD8, and H-$2D^b$ HPV16 $E7_{49–57}$ tetramer (MBL, no. TB-5008-1), followed by flow cytometry analysis.

Depletion assay

C57/BL6 mice were administered 200 μL each of control liposomes (Formumax, no. F70101-A) and clodronate liposomes (Formumax, no. F70101C-A) via intraperitoneal injection once per week for 2 weeks. One day after liposome injection, E and EUCV were each administered at 5 nmol. Seven days after the last injection, cells were harvested from the inguinal draining LNs, and rbc lysis was performed. To analyze depletion, the cells were stained with anti-CD3, anti-CD11b, and anti-CD11c antibodies for 30 min at 4°C, then measured by flow cytometry. Antigen-specific immune responses were assessed by seeding an equal number of cells isolated from the inguinal draining LNs of mice treated with each liposome, re-stimulated with E7 (49–57 aa), and evaluated via ELISpot. Antigen-specific $CD8^+$ T cells were measured using the $E7_{49–57}$ tetramer.

Tumor implantation and immunization

TC-1 tumor cells expressing HPV16-E6 and E7 proteins and E.G7-OVA cells expressing chicken OVA were used to generate of mouse tumor model. For the therapeutic cancer vaccine model, 7-week-old C57BL/6 mice were subcutaneously injected on the right flank with TC-1 cells (1×10^5 cells per mouse) in 100 μL DPBS. On day 6 when tumors measured 100 mm^3 in diameter, treatment with 5 nmol of E, E+U, and EUCV was initiated as indicated in the schedule. On day 20 mice were sacrificed, and tumor tissues were collected for TILs analysis. For the anti-PD-1 antibody combination model, 5 nmol of E and EUCV was injected subcutaneously, and 200 μg of anti-PD-1 antibody (BioXcell, clone 29F.1A12, no. BE0273) was injected intraperitoneally as indicated in the schedule. On day 20 mice were sacrificed, and tumor tissues were collected for qRT-PCR and immunohistochemistry analysis. Survival was recorded by observing the mice up to 60 days and mice were euthanized when tumor volume reached 1,500 mm^3. For the E.G7-OVA tumor model, 7-week-old C57BL/6 mice were subcutaneously injected on the right flank with E.G7-OVA cells (1×10^6 cells per mouse) in 100 μL DPBS. On day 3 when the tumor measured 100 mm^3 in diameter, treatment with 5 nmol of O, O+U, and OUCV was initiated as indicated in the schedule. On day 17 mice were sacrificed, and tumor tissue was collected for TIL analysis. In all experiments, tumors were measured 3 times a week using a caliper and calculated using the

formula V (volume : mm^3) $= L$ (length) $\times W^2$(width) $\times$ 0.52,[5] and mice were euthanize when tumor volume reached 1,500 mm^3.

Tumor and organ dissociation and analysis

TC-1 and E.G7-OVA tumors were collected after euthanasia and transferred to a dissociation buffer consisting of collagenase type IV (Roche, no. 17104019) and DNase I (Roche, no. 89836) in RPMI-1640 complete medium. The tumors were cut into small pieces (2–3 mm) using scissors and incubated for 1 h at 37°C. After incubation, dissociated tumors were passed through a 70-μm cell strainer (Miltenyi Biotec, no. 130-110-916) to obtain single-cell suspensions and centrifuged at 450 × g for 5 min at 4°C. The cells were resuspended in 45% Percoll solution (Cytiva, no. 17089101) layered on top of 70% Percoll solution and centrifuged at 400 × g for 45 min at 20°C (with accel = 0 and brake = 0). After suctioning out the lipid layer in the top parts of the supernatant, cells were collected in the middle parts of the supernatant using a dropping pipette. Red blood cells were removed using Pharm Lyse lysing solution and washed with 10 mL of PBS. To analyze TILs, cells were resuspended in a staining buffer (2% BSA in PBS) and stained with 1 μg/mL of anti-CD3, anti-CD8, anti-CD45, and H-2D^b HPV16 E7$_{49–57}$ tetramer for 30 min at 4°C, followed by flow cytometry analysis. For intracellular staining, TILs were re-stimulated with 2 μg/mL of E7$_{49–57}$ peptide for 16 h in the presence of GolgiPlug (1:1,000, BD, nos. 555029) and fixed using Cytofix/Cytoperm solution (BD) according to the manufacturer's instructions. Cells were stained with 1 μg/mL of anti-CD3, anti-CD8, anti-IFN-γ, and anti-TNF-α for 30 min at 4°C followed by flow cytometry analysis. Spleen and inguinal draining LNs were collected immediately after euthanasia and transferred to RPMI-1640 complete medium. Each organ was processed through a 70-μm cell strainer, and red blood cells were removed using Pharm Lyse lysing solution. All cells were washed 2 times with 10 mL of PBS.

qRT-PCR

Total RNA was extracted from tumor tissue using RNAmini (QIAGEN, no. 74004), quantified by measuring the absorbance at 260 nm using NanoDrop (Thermo Fisher, no. ND-2000), and reverse transcribed into cDNAs using the Maxima First Strand cDNA Synthesis Kit (Thermo Fisher, no. K1642) according to the manufacturer's instructions. Gene expression was analyzed by qRT-PCR with the Thermal Cycler Dice Real-Time System III (Takara). PCR amplification reactions were performed in a final reaction volume of 25 μL with TB Green premix Ex Taq (Takara, no. RR420), forward and reverse primer (10 μM, Bionicsro), Template cDNA (<10 ng), and sterile purified water. Primer sequences used are listed in Table S4 and GAPDH was used as a reference gene for normalizing gene expression. All reactions were performed in triplicate and relative expression was calculated by the $2^{-\Delta\Delta Ct}$ method. Gene expression levels were validated in threshold cycle (Ct) using Thermal Cycler Dice Real-Time System Software and compared using the t test.

Immunohistochemistry

Tumor tissues were harvested, fixed with 4% paraformaldehyde for a day, and embedded in paraffin for generation paraffin block. Five-micromillimeter-thick sections were cut from paraffin-embedded tumors using a section tool and collected on glass slides. For staining, section slides were incubated for 10 min at 60°C oven and deparaffinized using a processor. For antigen retrieval, slides were boiled with citrate buffer for 10 min and blocked with blocking buffer (goat serum) for 10 min at room temperature. After blocking, slides were stained with primary antibodies diluted in blocking buffer overnight at 4°C in a humidified chamber. On the following day, slides were washed with wash buffer (0.05% Tween 20 containing PBS) and stained with secondary antibodies for 1 h at room temperature. After incubation, slides were washed with wash buffer and developed with chromogen solution. Next, slides were washed with deionized water for 5 min and stained with hematoxylin solution for 5 min at room temperature for counterstaining. For rehydration, slides were incubated in the processor and covered with coverslips (Marienfeld, no. 010115) using mounting medium (Biomeda, no. M01), followed by drying in a fume hood. All slides were recorded using a slide scanner with CaseViewer software (3DHISTECH) and H-score analysis was performed using ImageJ software (FIJI). All of these analyses were performed in three random fields per slide at 400× magnification.

ELISpot

Pre-coated ELISpot plates (Mabtech, no. 3321-4AST-10) were washed 3 times with PBS and blocked using 100 μL of RPMI medium supplemented with 10% FBS, 1% streptomycin, and penicillin for 1 h. After blocking, splenocytes and inguinal draining LN cells from C57BL/6 mice were seeded in the ELISpot plate with duplicates (5 × 10^5 cells per well) and incubated for 24 h at 37°C and 5% CO_2 with the following stimuli: 2 μg/mL of HPV16 E6 (29–37, 49–57, 129–138), HPV16 E7 (11–19, 49–57, 81–90), and OVA (257–264). After 24 h, plates were washed 4 times with PBS, and anti-mouse IFN-γ (Mabtech) was added at 100 μL/well (1:1,000 in 0.5% FBS containing PBS) for 2 h at room temperature. Next, the plates were washed 4 times with PBS, and SA-ALP (Mabtech) was added at 100 μL/well (1:1,000 in 0.5% FBS containing PBS) for 2 h at room temperature. After incubation, the plates were washed 4 times with PBS, and developed by adding 100 μL/well BCIP solution (Mabtech). The ELIspot plates were read using an AID EliSpot Reader and analyzed with the AID EliSpot software 7.0.

In vitro and *in vivo* toxicity

The *in vitro* cytotoxicity test was assessed using Cell Counting Kit (CCK)-8 assays (Dojindo, no. CK04). DC2.4, Raw 264.7, and TC-1 cells were seeded onto 96-well plates for 24 h before treating them with different concentrations of EUCV and doxorubicin. A 10% CCK-8 solution was added to each well 18 h after treatment, and the plates were incubated at 37°C in a 5% CO_2 atmosphere for 30 min to 4 h. The absorbance at 450 nm was detected using an instrument. Relative cell viability was calculated as a percentage of untreated control cells. To assess *in vivo* toxicity, E7 (100.6 nmol) and EUCV at 25 mpk (25.2 nmol) and 100 mpk (100.6 nmol) were subcutaneously injected into the backs of C57BL/6 mice. As controls, we used poly(I:C) (2.5 nmol) and Pam2CSK4 (100.6 nmol). Mouse body weight was measured immediately after administration and at

24 h intervals, and at 48 h the mice were euthanized to measure spleen size to evaluate splenomegaly. After vaccine administration, blood was drawn at 2, 8, and 24 h, and cytokines IL-6 (BD, no. 555240) and IL-12p40 (BD, no. 555165) in serum were quantified using ELISA.

Statistical analysis

All statistical analysis was performed using GraphPad Prism software v.7.0, and data were presented as mean ± SD or SEM. Significance values for each result were calculated using a Student's t test and two-way ANOVA (Tukey multiple comparison tests), and $p < 0.05$ was considered as statistically significant. For the survival curve, statistical significance analysis was performed using Kaplan-Meier with the log rank (Mantel-Cox) test.

DATA AND CODE AVAILABILITY

All data related to the research is incorporated in the article or provided as supplemental information. The information can be obtained from the corresponding authors upon a reasonable request.

SUPPLEMENTAL INFORMATION

Supplemental information can be found online at https://doi.org/10.1016/j.ymthe.2024.07.014.

ACKNOWLEDGMENTS

We would like to thank Eun-Kyeong Jo and Myeong Hee Kim for providing TLR2$^{-/-}$ mice. This work was supported by the National Research Foundation of Korea (NRF) grant funded by the Korean government (MSIT) (2021R1A3B1076605), NRF grant funded by Korean government (MSIT) (2021R1C1C1006496, 2022R1C1C1013481, and 2021R1C1C1013332), and Yonsei University Research Fund (2021-22-0293, 2021-22-0291, 2021-22-0084, and 2024-22-0060).

AUTHOR CONTRIBUTIONS

Conceptualization, H.Y.K., S.C., S.B.K., and S.K.; formal analysis, H.Y.K. and S.C.; validation, H.Y.K.; visualization, H.Y.K., S.C., and J.C.; methodology, H.Y.K., S.C., E.C.S., W.J., Y.G.S., J.H.S., I.Y., U.K., H.B., S.H., J.M., J.P., N.K., and Y.L.; writing – original draft, H.Y.K., S.C., and S.K.; writing – review & editing, S.K.; funding acquisition, S.C., S.B.K., I.Y., and S.K.; resources, S.B.K., M.H.K., and S.K.; investigation, E.C.S., W.J., Y.G.S., J.H.S., J.C., I.Y., U.K., H.B., S.H., J.M., J.P., N.K., and Y.L.; supervision, M.H.K. and S.K.; project administration, S.K.

DECLARATION OF INTERESTS

S.K. is an inventor on patents 10-2022-0070287 related to this paper and is a founder of Zymedi.

REFERENCES

1. Kwon, N.H., Fox, P.L., and Kim, S. (2019). Aminoacyl-tRNA synthetases as therapeutic targets. Nat. Rev. Drug Discov. *18*, 629–650. https://doi.org/10.1038/s41573-019-0026-3.
2. Nguyen, T.T.T., Choi, Y.H., Lee, W.K., Ji, Y., Chun, E., Kim, Y.H., Lee, J.E., Jung, H.S., Suh, J.H., Kim, S., and Jin, M. (2023). Tryptophan-dependent and -independent secretions of tryptophanyl- tRNA synthetase mediate innate inflammatory responses. Cell Rep. *42*, 111905. https://doi.org/10.1016/j.celrep.2022.111905.
3. Park, M.C., Goughnour, P.C., Jun, S., Cho, S., Song, E., Kim, S.B., Kim, H.Y., Hyun, J.K., Kim, P., Jung, H.S., and Kim, S. (2022). Two distinct receptor-binding domains of human glycyl-tRNA synthetase 1 displayed on extracellular vesicles activate M1 polarization and phagocytic bridging of macrophages to cancer cells. Cancer Lett. *539*, 215698. https://doi.org/10.1016/j.canlet.2022.215698.
4. Guo, M., Yang, X.L., and Schimmel, P. (2010). New functions of aminoacyl-tRNA synthetases beyond translation. Nat. Rev. Mol. Cell Biol. *11*, 668–674. https://doi.org/10.1038/nrm2956.
5. Cho, S., Kim, S.B., Lee, Y., Song, E.C., Kim, U., Kim, H.Y., Suh, J.H., Goughnour, P.C., Kim, Y., Yoon, I., et al. (2020). Endogenous TLR2 ligand embedded in the catalytic region of human cysteinyl-tRNA synthetase 1. J. Immunother. Cancer *8*, e000277. https://doi.org/10.1136/jitc-2019-000277.
6. Maeng, H.M., and Berzofsky, J.A. (2019). Strategies for developing and optimizing cancer vaccines. F1000Res. *8*. F1000 Faculty Rev-654. https://doi.org/10.12688/f1000research.18693.1.
7. Rosalia, R.A., Quakkelaar, E.D., Redeker, A., Khan, S., Camps, M., Drijfhout, J.W., Silva, A.L., Jiskoot, W., van Hall, T., van Veelen, P.A., et al. (2013). Dendritic cells process synthetic long peptides better than whole protein, improving antigen presentation and T-cell activation. Eur. J. Immunol. *43*, 2554–2565. https://doi.org/10.1002/eji.201343324.
8. Hollingsworth, R.E., and Jansen, K. (2019). Turning the corner on therapeutic cancer vaccines. NPJ Vaccin. *4*, 7. https://doi.org/10.1038/s41541-019-0103-y.
9. Janes, M.E., Gottlieb, A.P., Park, K.S., Zhao, Z., and Mitragotri, S. (2024). Cancer vaccines in the clinic. Bioeng. Transl. Med. *9*, e10588. https://doi.org/10.1002/btm2.10588.
10. Liu, H., Moynihan, K.D., Zheng, Y., Szeto, G.L., Li, A.V., Huang, B., Van Egeren, D.S., Park, C., and Irvine, D.J. (2014). Structure-based programming of lymph-node targeting in molecular vaccines. Nature *507*, 519–522. https://doi.org/10.1038/nature12978.
11. McLennan, D.N., Porter, C.J.H., and Charman, S.A. (2005). Subcutaneous drug delivery and the role of the lymphatics. Drug Discov. Today Technol. *2*, 89–96. https://doi.org/10.1016/j.ddtec.2005.05.006.
12. Arabpour, M., Paul, S., Grauers Wiktorin, H., Kaya, M., Kiffin, R., Lycke, N., Hellstrand, K., and Martner, A. (2022). An adjuvant-containing cDC1-targeted recombinant fusion vaccine conveys strong protection against murine melanoma growth and metastasis. Oncoimmunology *11*, 2115618. https://doi.org/10.1080/2162402X.2022.2115618.
13. Mehta, N.K., Pradhan, R.V., Soleimany, A.P., Moynihan, K.D., Rothschilds, A.M., Momin, N., Rakhra, K., Mata-Fink, J., Bhatia, S.N., Wittrup, K.D., and Irvine, D.J. (2020). Pharmacokinetic tuning of protein-antigen fusions enhances the immunogenicity of T-cell vaccines. Nat. Biomed. Eng. *4*, 636–648. https://doi.org/10.1038/s41551-020-0563-4.
14. Hailemichael, Y., Dai, Z., Jaffarzad, N., Ye, Y., Medina, M.A., Huang, X.F., Dorta-Estremera, S.M., Greeley, N.R., Nitti, G., Peng, W., et al. (2013). Persistent antigen at vaccination sites induces tumor-specific CD8(+) T cell sequestration, dysfunction and deletion. Nat. Med. *19*, 465–472. https://doi.org/10.1038/nm.3105.
15. Tzakos, A.G., Briasoulis, E., Thalhammer, T., Jäger, W., and Apostolopoulos, V. (2013). Novel oncology therapeutics: targeted drug delivery for cancer. J. Drug Deliv. *2013*, 918304. https://doi.org/10.1155/2013/918304.
16. Mueller, S.N. (2017). Spreading the load: Antigen transfer between migratory and lymph node-resident dendritic cells promotes T-cell priming. Eur. J. Immunol. *47*, 1798–1801. https://doi.org/10.1002/eji.201747248.
17. Vu Manh, T.P., Bertho, N., Hosmalin, A., Schwartz-Cornil, I., and Dalod, M. (2015). Investigating Evolutionary Conservation of Dendritic Cell Subset Identity and Functions. Front. Immunol. *6*, 260. https://doi.org/10.3389/fimmu.2015.00260.
18. Barry, K.C., Hsu, J., Broz, M.L., Cueto, F.J., Binnewies, M., Combes, A.J., Nelson, A.E., Loo, K., Kumar, R., Rosenblum, M.D., et al. (2018). A natural killer-dendritic cell axis defines checkpoint therapy-responsive tumor microenvironments. Nat. Med. *24*, 1178–1191. https://doi.org/10.1038/s41591-018-0085-8.

19. Jabbar, B., Rafique, S., Salo-Ahen, O.M.H., Ali, A., Munir, M., Idrees, M., Mirza, M.U., Vanmeert, M., Shah, S.Z., Jabbar, I., and Rana, M.A. (2018). Antigenic Peptide Prediction From E6 and E7 Oncoproteins of HPV Types 16 and 18 for Therapeutic Vaccine Design Using Immunoinformatics and MD Simulation Analysis. Front. Immunol. *9*, 3000. https://doi.org/10.3389/fimmu.2018.03000.

20. Chen, X., Zaro, J.L., and Shen, W.C. (2013). Fusion protein linkers: property, design and functionality. Adv. Drug Deliv. Rev. *65*, 1357–1369. https://doi.org/10.1016/j.addr.2012.09.039.

21. Patente, T.A., Pinho, M.P., Oliveira, A.A., Evangelista, G.C.M., Bergami-Santos, P.C., and Barbuto, J.A.M. (2018). Human Dendritic Cells: Their Heterogeneity and Clinical Application Potential in Cancer Immunotherapy. Front. Immunol. *9*, 3176. https://doi.org/10.3389/fimmu.2018.03176.

22. Ghimire, T.R., Benson, R.A., Garside, P., and Brewer, J.M. (2012). Alum increases antigen uptake, reduces antigen degradation and sustains antigen presentation by DCs *in vitro*. Immunol. Lett. *147*, 55–62. https://doi.org/10.1016/j.imlet.2012.06.002.

23. Haniffa, M., Collin, M., and Ginhoux, F. (2013). Identification of human tissue cross-presenting dendritic cells: A new target for cancer vaccines. Oncoimmunology *2*, e23140. https://doi.org/10.4161/onci.23140.

24. Kuhn, S., Yang, J., and Ronchese, F. (2015). Monocyte-Derived Dendritic Cells Are Essential for CD8(+) T Cell Activation and Antitumor Responses After Local Immunotherapy. Front. Immunol. *6*, 584. https://doi.org/10.3389/fimmu.2015.00584.

25. Chang, H.C., Zou, Z.Z., Wang, Q.H., Li, J., Jin, H., Yin, Q.X., and Xing, D. (2020). Targeting and Specific Activation of Antigen-Presenting Cells by Endogenous Antigen-Loaded Nanoparticles Elicits Tumor-Specific Immunity. Adv. Sci. *7*, 1900069. https://doi.org/10.1002/advs.201900069.

26. Joffre, O.P., Segura, E., Savina, A., and Amigorena, S. (2012). Cross-presentation by dendritic cells. Nat. Rev. Immunol. *12*, 557–569. https://doi.org/10.1038/nri3254.

27. Lee, C.H., Nawar, H.F., Mandell, L., Liang, S., Hajishengallis, G., and Connell, T.D. (2010). Enhanced antigen uptake by dendritic cells induced by the B pentamer of the type II heat-labile enterotoxin LT-IIa requires engagement of TLR2. Vaccine *28*, 3696–3705. https://doi.org/10.1016/j.vaccine.2010.03.016.

28. Shen, K.Y., Song, Y.C., Chen, I.H., Leng, C.H., Chen, H.W., Li, H.J., Chong, P., and Liu, S.J. (2014). Molecular mechanisms of TLR2-mediated antigen cross-presentation in dendritic cells. J. Immunol. *192*, 4233–4241. https://doi.org/10.4049/jimmunol.1302850.

29. Auray, G., Talker, S.C., Keller, I., Python, S., Gerber, M., Liniger, M., Ganges, L., Bruggmann, R., Ruggli, N., and Summerfield, A. (2020). High-Resolution Profiling of Innate Immune Responses by Porcine Dendritic Cell Subsets *in vitro* and *in vivo*. Front. Immunol. *11*, 1429. https://doi.org/10.3389/fimmu.2020.01429.

30. Villani, A.C., Satija, R., Reynolds, G., Sarkizova, S., Shekhar, K., Fletcher, J., Griesbeck, M., Butler, A., Zheng, S., Lazo, S., et al. (2017). Single-cell RNA-seq reveals new types of human blood dendritic cells, monocytes, and progenitors. Science *356*, eaah4573. https://doi.org/10.1126/science.aah4573.

31. Collin, M., and Bigley, V. (2018). Human dendritic cell subsets: an update. Immunology *154*, 3–20. https://doi.org/10.1111/imm.12888.

32. Randolph, G.J., Ivanov, S., Zinselmeyer, B.H., and Scallan, J.P. (2017). The Lymphatic System: Integral Roles in Immunity. Annu. Rev. Immunol. *35*, 31–52. https://doi.org/10.1146/annurev-immunol-041015-055354.

33. Ghosh, M., and Shapiro, L.H. (2012). *In vitro* Ag Cross-presentation and *in vivo* Ag Cross-presentation by Dendritic Cells in the Mouse. Bio. Protoc. *2*, e305. https://doi.org/10.21769/bioprotoc.305.

34. Liu, Y., Wu, L., Tong, R., Yang, F., Yin, L., Li, M., You, L., Xue, J., and Lu, Y. (2019). PD-1/PD-L1 Inhibitors in Cervical Cancer. Front. Pharmacol. *10*, 65. https://doi.org/10.3389/fphar.2019.00065.

35. Verma, V., Shrimali, R.K., Ahmad, S., Dai, W., Wang, H., Lu, S., Nandre, R., Gaur, P., Lopez, J., Sade-Feldman, M., et al. (2019). PD-1 blockade in subprimed CD8 cells induces dysfunctional PD-1(+)CD38(hi) cells and anti-PD-1 resistance. Nat. Immunol. *20*, 1231–1243. https://doi.org/10.1038/s41590-019-0441-y.

36. Chung, H.C., Ros, W., Delord, J.P., Perets, R., Italiano, A., Shapira-Frommer, R., Manzuk, L., Piha-Paul, S.A., Xu, L., Zeigenfuss, S., et al. (2019). Efficacy and Safety of Pembrolizumab in Previously Treated Advanced Cervical Cancer: Results From the Phase II KEYNOTE-158 Study. J. Clin. Oncol. *37*, 1470–1478. https://doi.org/10.1200/JCO.18.01265.

37. Marcovecchio, P.M., Thomas, G., and Salek-Ardakani, S. (2021). CXCL9-expressing tumor-associated macrophages: new players in the fight against cancer. J. Immunother. Cancer *9*, e002045. https://doi.org/10.1136/jitc-2020-002045.

38. Anwar, M.A., Shah, M., Kim, J., and Choi, S. (2019). Recent clinical trends in Toll-like receptor targeting therapeutics. Med. Res. Rev. *39*, 1053–1090. https://doi.org/10.1002/med.21553.

39. Roberts, E.W., Broz, M.L., Binnewies, M., Headley, M.B., Nelson, A.E., Wolf, D.M., Kaisho, T., Bogunovic, D., Bhardwaj, N., and Krummel, M.F. (2016). Critical Role for CD103(+)/CD141(+) Dendritic Cells Bearing CCR7 for Tumor Antigen Trafficking and Priming of T Cell Immunity in Melanoma. Cancer Cell *30*, 324–336. https://doi.org/10.1016/j.ccell.2016.06.003.

40. Ruhland, M.K., Roberts, E.W., Cai, E., Mujal, A.M., Marchuk, K., Beppler, C., Nam, D., Serwas, N.K., Binnewies, M., and Krummel, M.F. (2020). Visualizing Synaptic Transfer of Tumor Antigens among Dendritic Cells. Cancer Cell *37*, 786–799.e5. https://doi.org/10.1016/j.ccell.2020.05.002.

41. Bhardwaj, N., Friedlander, P.A., Pavlick, A.C., Ernstoff, M.S., Gastman, B.R., Hanks, B.A., Curti, B.D., Albertini, M.R., Luke, J.J., Blazquez, A.B., et al. (2020). Flt3 ligand augments immune responses to anti-DEC-205-NY-ESO-1 vaccine through expansion of dendritic cell subsets. Nat. Cancer *1*, 1204–1217. https://doi.org/10.1038/s43018-020-00143-y.

42. Eisenbarth, S.C. (2019). Dendritic cell subsets in T cell programming: location dictates function. Nat. Rev. Immunol. *19*, 89–103. https://doi.org/10.1038/s41577-018-0088-1.

43. Seya, T., Shime, H., Takeda, Y., Tatematsu, M., Takashima, K., and Matsumoto, M. (2015). Adjuvant for vaccine immunotherapy of cancer – focusing on Toll-like receptor 2 and 3 agonists for safely enhancing antitumor immunity. Cancer Sci. *106*, 1659–1668. https://doi.org/10.1111/cas.12824.

44. Tullett, K.M., Leal Rojas, I.M., Minoda, Y., Tan, P.S., Zhang, J.G., Smith, C., Khanna, R., Shortman, K., Caminschi, I., Lahoud, M.H., and Radford, K.J. (2016). Targeting CLEC9A delivers antigen to human CD141(+) DC for CD4(+) and CD8(+)T cell recognition. JCI Insight *1*, e87102. https://doi.org/10.1172/jci.insight.87102.

45. Lee, Y.S., O'Brien, L.J., Walpole, C.M., Pearson, F.E., Leal-Rojas, I.M., Masterman, K.A., Atkinson, V., Barbour, A., and Radford, K.J. (2021). Human CD141(+) dendritic cells (cDC1) are impaired in patients with advanced melanoma but can be targeted to enhance anti-PD-1 in a humanized mouse model. J. Immunother. Cancer *9*, e001963. https://doi.org/10.1136/jitc-2020-001963.

46. Supersaxo, A., Hein, W.R., and Steffen, H. (1990). Effect of molecular weight on the lymphatic absorption of water-soluble compounds following subcutaneous administration. Pharm. Res. *7*, 167–169. https://doi.org/10.1023/a:1015880819328.

47. Schliehe, C., Redaelli, C., Engelhardt, S., Fehlings, M., Mueller, M., van Rooijen, N., Thiry, M., Hildner, K., Weller, H., and Groettrup, M. (2011). CD8- dendritic cells and macrophages cross-present poly(D,L-lactate-co-glycolate) acid microsphere-encapsulated antigen *in vivo*. J. Immunol. *187*, 2112–2121. https://doi.org/10.4049/jimmunol.1002084.

48. Zhang, T., Aipire, A., Li, Y., Guo, C., and Li, J. (2023). Antigen cross-presentation in dendric cells: From bench to bedside. Biomed. Pharmacother. *168*, 115758. https://doi.org/10.1016/j.biopha.2023.115758.

49. Khan, S., Bijker, M.S., Weterings, J.J., Tanke, H.J., Adema, G.J., van Hall, T., Drijfhout, J.W., Melief, C.J.M., Overkleeft, H.S., van der Marel, G.A., et al. (2007). Distinct uptake mechanisms but similar intracellular processing of two different toll-like receptor ligand-peptide conjugates in dendritic cells. J. Biol. Chem. *282*, 21145–21159. https://doi.org/10.1074/jbc.M701705200.

50. Pishesha, N., Harmand, T.J., and Ploegh, H.L. (2022). A guide to antigen processing and presentation. Nat. Rev. Immunol. *22*, 751–764. https://doi.org/10.1038/s41577-022-00707-2.

51. Blander, J.M. (2018). Regulation of the Cell Biology of Antigen Cross-Presentation. Annu. Rev. Immunol. *36*, 717–753. https://doi.org/10.1146/annurev-immunol-041015-055523.

52. Marijt, K.A., Griffioen, L., Blijleven, L., van der Burg, S.H., and van Hall, T. (2022). Cross-presentation of a TAP-independent signal peptide induces CD8 T immunity

to escaped cancers but necessitates anchor replacement. Cancer Immunol. Immunother. *71*, 289–300. https://doi.org/10.1007/s00262-021-02984-7.

53. Croft, N.P., Shannon-Lowe, C., Bell, A.I., Horst, D., Kremmer, E., Ressing, M.E., Wiertz, E.J.H.J., Middeldorp, J.M., Rowe, M., Rickinson, A.B., and Hislop, A.D. (2009). Stage-specific inhibition of MHC class I presentation by the Epstein-Barr virus BNLF2a protein during virus lytic cycle. Plos Pathog. *5*, e1000490. https://doi.org/10.1371/journal.ppat.1000490.

54. Hansen, S.G., Powers, C.J., Richards, R., Ventura, A.B., Ford, J.C., Siess, D., Axthelm, M.K., Nelson, J.A., Jarvis, M.A., Picker, L.J., and Früh, K. (2010). Evasion of CD8+ T cells is critical for superinfection by cytomegalovirus. Science *328*, 102–106. https://doi.org/10.1126/science.1185350.

55. Bijker, M.S., van den Eeden, S.J.F., Franken, K.L., Melief, C.J.M., van der Burg, S.H., and Offringa, R. (2008). Superior induction of anti-tumor CTL immunity by extended peptide vaccines involves prolonged, DC-focused antigen presentation. Eur. J. Immunol. *38*, 1033–1042. https://doi.org/10.1002/eji.200737995.

56. Bezu, L., Kepp, O., Cerrato, G., Pol, J., Fucikova, J., Spisek, R., Zitvogel, L., Kroemer, G., and Galluzzi, L. (2018). Trial watch: Peptide-based vaccines in anticancer therapy. Oncoimmunology *7*, e1511506. https://doi.org/10.1080/2162402X.2018.1511506.

57. Khazaei, S., Varela-Calvino, R., Rad-Malekshahi, M., Quattrini, F., Jokar, S., Rezaei, N., Balalaie, S., Haririan, I., Csaba, N., and Garcia-Fuentes, M. (2024). Self-assembled peptide/polymer hybrid nanoplatform for cancer immunostimulating therapies. Drug Deliv. Transl Res. *14*, 455–473. https://doi.org/10.1007/s13346-023-01410-y.

58. Li, Y.D., Chi, W.Y., Su, J.H., Ferrall, L., Hung, C.F., and Wu, T.C. (2020). Coronavirus vaccine development: from SARS and MERS to COVID-19. J. Biomed. Sci. *27*, 104. https://doi.org/10.1186/s12929-020-00695-2.

59. Qin, F., Xia, F., Chen, H., Cui, B., Feng, Y., Zhang, P., Chen, J., and Luo, M. (2021). A Guide to Nucleic Acid Vaccines in the Prevention and Treatment of Infectious Diseases and Cancers: From Basic Principles to Current Applications. Front. Cell Dev. Biol. *9*, 633776. https://doi.org/10.3389/fcell.2021.633776.

60. Khairkhah, N., Bolhassani, A., and Najafipour, R. (2022). Current and future direction in treatment of HPV-related cervical disease. J. Mol. Med. *100*, 829–845. https://doi.org/10.1007/s00109-022-02199-y.

61. Hallez, S., Brulet, J.M., Vandooren, C., Maudoux, F., Thomas, S., Heinderickx, M., Bollen, A., Wattiez, R., and Jacquet, A. (2004). Pre-clinical immunogenicity and anti-tumour efficacy of a deleted recombinant human papillomavirus type 16 E7 protein. Anticancer Res. *24*, 2265–2275.

62. Zehbe, I., Wilander, E., Delius, H., and Tommasino, M. (1998). Human papillomavirus 16 E6 variants are more prevalent in invasive cervical carcinoma than the prototype. Cancer Res. *58*, 829–833.

63. Karadza, M., Zidovec Lepej, S., Planinic, A., Grgic, I., Corusic, A., Planinic, P., Coric, M., Hosnjak, L., Fujs Komlos, K., Poljak, M., and Vince, A. (2021). Distribution of human papillomavirus genotypes in women with high-grade cervical intraepithelial lesions and cervical carcinoma and analysis of human papillomavirus-16 genomic variants. Croat. Med. J. *62*, 68–79. https://doi.org/10.3325/cmj.2021.62.68.

64. Keskin, D.B., Reinhold, B., Lee, S.Y., Zhang, G., Lank, S., O'Connor, D.H., Berkowitz, R.S., Brusic, V., Kim, S.J., and Reinherz, E.L. (2011). Direct identification of an HPV-16 tumor antigen from cervical cancer biopsy specimens. Front. Immunol. *2*, 75. https://doi.org/10.3389/fimmu.2011.00075.

65. Bahmani, B., Amini-Bayat, Z., Ranjbar, M.M., Bakhtiari, N., and Zarnani, A.H. (2021). HPV16-E7 Protein T Cell Epitope Prediction and Global Therapeutic Peptide Vaccine Design Based on Human Leukocyte Antigen Frequency: An In-Silico Study. Int. J. Pept. Res. Ther. *27*, 365–378. https://doi.org/10.1007/s10989-020-10089-5.

66. Tseng, S.H., Liu, L., Peng, S., Kim, J., Ferrall, L., Hung, C.F., and Wu, T.C. (2021). Control of Spontaneous HPV16 E6/E7 Expressing Oral Cancer in HLA-A2 (AAD) Transgenic Mice with Therapeutic HPV DNA Vaccine. J. Biomed. Sci. *28*, 63. https://doi.org/10.1186/s12929-021-00759-x.

67. Darvin, P., Toor, S.M., Sasidharan Nair, V., and Elkord, E. (2018). Immune checkpoint inhibitors: recent progress and potential biomarkers. Exp. Mol. Med. *50*, 1–11. https://doi.org/10.1038/s12276-018-0191-1.

68. Colombo, N., Dubot, C., Lorusso, D., Caceres, M.V., Hasegawa, K., Shapira-Frommer, R., Tewari, K.S., Salman, P., Hoyos Usta, E., Yañez, E., et al. (2021). Pembrolizumab for Persistent, Recurrent, or Metastatic Cervical Cancer. N. Engl. J. Med. *385*, 1856–1867. https://doi.org/10.1056/NEJMoa2112435.

69. Trujillo, J.A., Sweis, R.F., Bao, R., and Luke, J.J. (2018). T Cell-Inflamed versus Non-T Cell-Inflamed Tumors: A Conceptual Framework for Cancer Immunotherapy Drug Development and Combination Therapy Selection. Cancer Immunol. Res. *6*, 990–1000. https://doi.org/10.1158/2326-6066.CIR-18-0277.

70. Wu, M., Huang, Q., Xie, Y., Wu, X., Ma, H., Zhang, Y., and Xia, Y. (2022). Improvement of the anticancer efficacy of PD-1/PD-L1 blockade via combination therapy and PD-L1 regulation. J. Hematol. Oncol. *15*, 24. https://doi.org/10.1186/s13045-022-01242-2.

71. Lee, W.S., Yang, H., Chon, H.J., and Kim, C. (2020). Combination of anti-angiogenic therapy and immune checkpoint blockade normalizes vascular-immune crosstalk to potentiate cancer immunity. Exp. Mol. Med. *52*, 1475–1485. https://doi.org/10.1038/s12276-020-00500-y.

72. Ahmed, T.A. (2015). Pharmacokinetics of Drugs Following IV Bolus, IV Infusion, and Oral Administration. In Basic Pharmacokinetic Concepts and Some Clinical Applications. https://doi.org/10.5772/61573.

YMTHE, Volume *32*

Supplemental Information

Specific targeting of cancer vaccines to antigen-presenting cells via an endogenous TLR2/6 ligand derived from cysteinyl-tRNA synthetase 1

Hyeong Yun Kim, Seongmin Cho, Sang Bum Kim, Ee Chan Song, Wonchul Jung, Yun Gyeong Shin, Ji Hun Suh, Jihye Choi, Ina Yoon, Uijoo Kim, Hamin Ban, Sunkyo Hwang, Jeongwon Mun, Joohee Park, Nayoung Kim, Youngjin Lee, Myung Hee Kim, and Sunghoon Kim

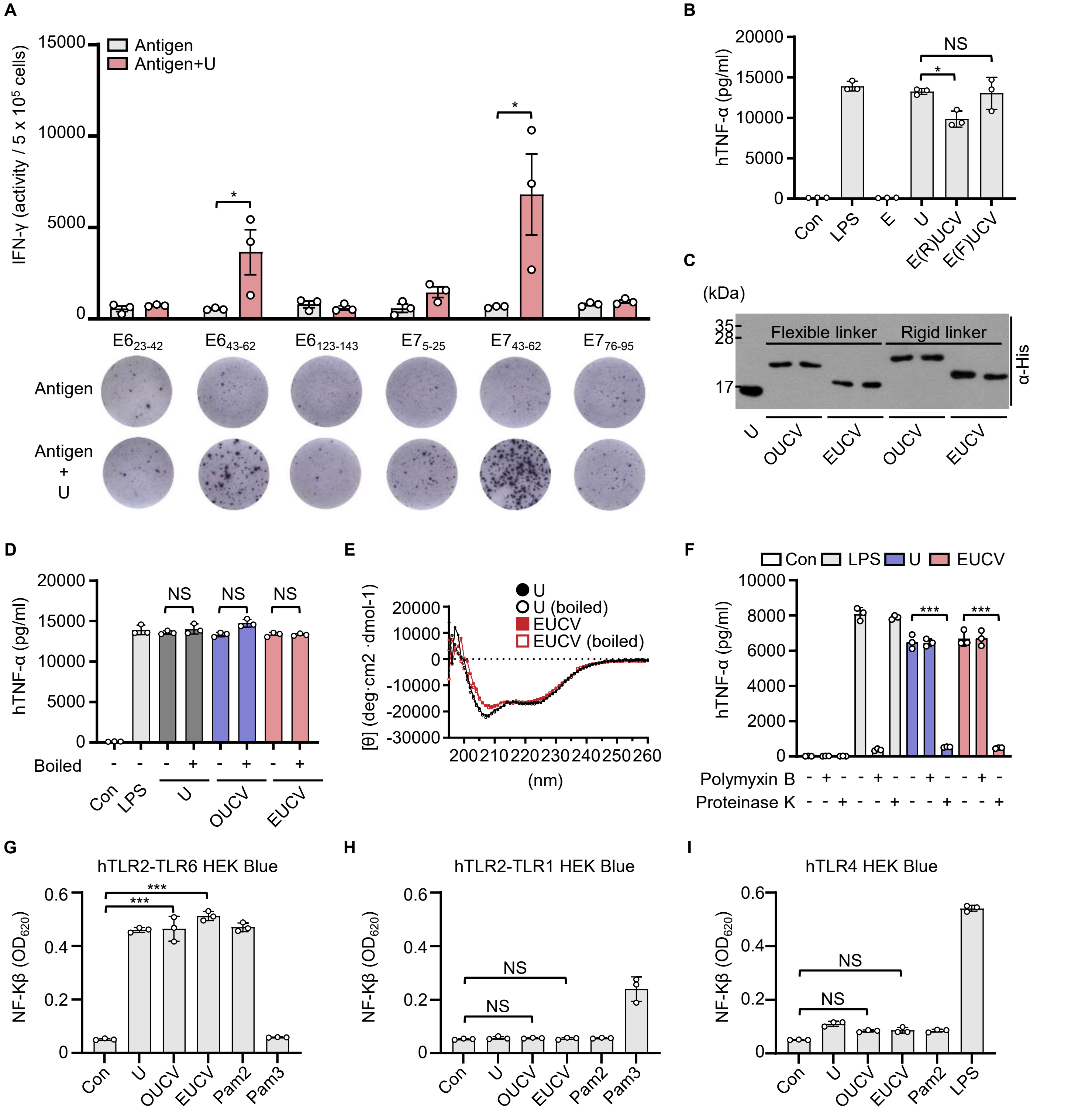

Fig S1. Optimization and physicochemical characterization of the UCV. (**A**) C57BL/6 mice were immunized twice at 2-week intervals with each of the HPV16 E6 or E 7 peptide antigens in combination with U (lower). Each peptide-specific IFN-γ-producing T-cell from the spleen was quantified using ELISpot after 1 week from final immu nization. (**B**) Production of TNF-α was measured by ELISA after the treatment of 100 nM of U, E(R)UCV, or E(F)UCV on PMA-differentiated THP-1 cells for 4 h. Some cel ls were treated with the E7 peptide and LPS as negative and positive controls, respectively. (**C**) Western blot analysis of U, EUCV, and OUCV (1 μg of total protein per lan e). Target bands were detected with an anti-His antibody. (**D**) PMA-differentiated THP-1 cells were treated with 100 nM of native and boiled U, EUCV, and OUCV for 4 h, TNF-α production was measured by ELISA. (**E**) Circular dichroism (CD) spectroscopy analysis of U and EUCV under native or boiled conditions at 195–260 nm. (**F**) PMA -differentiated THP-1 cells were treated with 100 nM U and EUCV for 4 h. U or EUCV were preincubated with proteinase K (50 μg/ml) for 1 h. Before treating U and EUC V, some cells were preincubated with polymyxin B (10 μg/ml) for 1 h, and LPS was used as a positive control. After 4 h of treatment, TNF-α production was measured by ELISA. (**G**–**I**) U, EUCV, and OUCV were treated with (**G**) hTLR2/6-, (**H**) hTLR2/1-, and (**I**) hTLR4- expressing HEK-Blue cells. SEAP activity was measured at OD_{620}. Pam 2CSK4, Pam3CSK4, and LPS were used as positive controls. Data are representative of three independent experiments, and the results are presented as mean ± SD. S tatistical significance was analyzed using Student's t-test (**P* < 0.05, ***P* < 0.01, ****P* < 0.001).

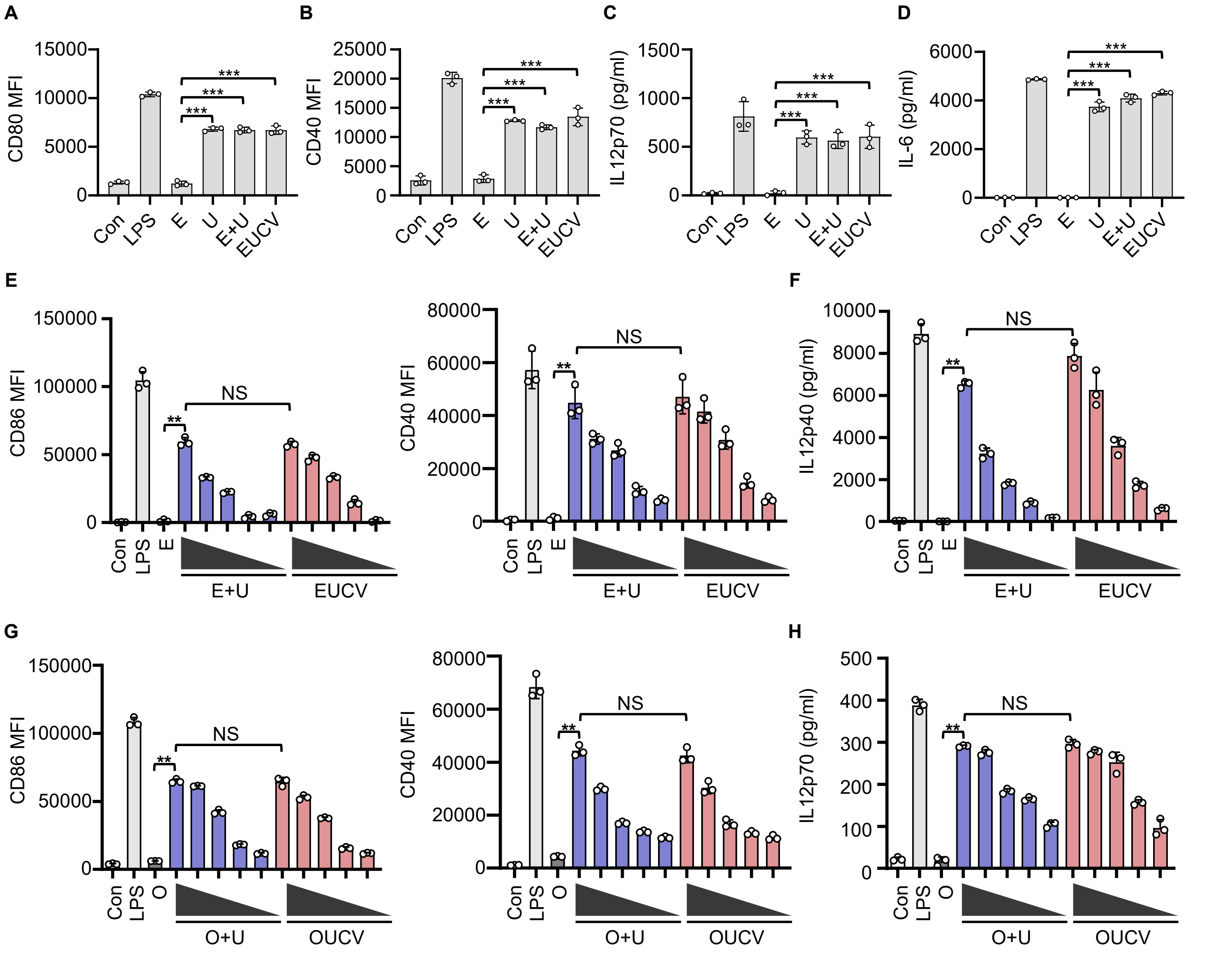

Fig S2. Comparison of the immune stimulation activities among UCV in APCs. (**A**–**D**) BMDCs were incubated with 100 nM E, U, E+U, and EUCV for 24 h. Costimulatory molecules were analyzed from the $CD11c^+$ gating population, and (**A**) CD80 or (**B**) CD40 expression was evaluated by flow cytometry. The production of (**C**) IL-12p70 and (**D**) IL-6 was quantified by ELISA. E and LPS were used as negative and positive controls, respectively. (**E**, **F**) BMDCs were incubated with 100 nM E, E+U, and EUCV for 24 h. (**E**) Costimulatory molecules were analyzed from the $CD11c^+$ gating population, and CD86 or CD40 expression was evaluated by flow cytometry. (**F**) IL-12p40 production was quantified by ELISA. E and LPS were used as negative and positive controls, respectively. (**G**, **H**) BMDCs were incubated with 100 nM O, O+U, and OUCV for 24 h. (**G**) Costimulatory molecules were analyzed from the $CD11c^+$ gating population, and CD86 and CD40 expression was evaluated by flow cytometry. (**H**) IL-12p70 production was quantified by ELISA. O and LPS were used as negative and positive controls, respectively. Data are representative of three independent experiments, and the results are presented as the mean ± SD. Statistical significance was analyzed using Student's t-test (***$P < 0.001$). NS, not significant.

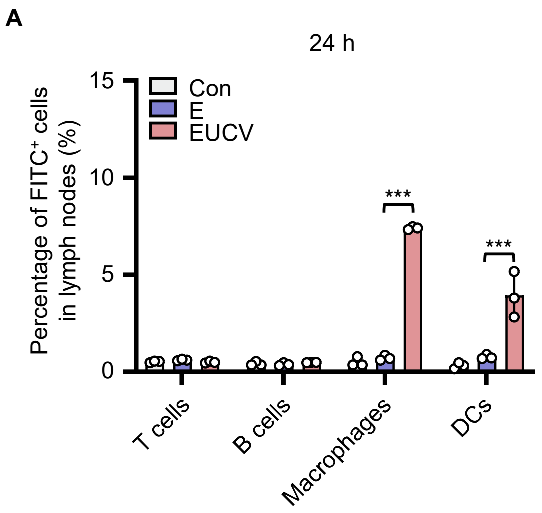

Fig S3. Comparison of antigen uptake among different cell types in lymph nodes. (**A**) C57BL/6 mice were subcutaneously injected with 5 nmol of FAM-labeled E or EUCV. Immune cells were harvested from excised inguinal lymph nodes at 24 h after injection, and the 5-FAM signal in various cell types was assessed by flow cytometry (*n* = 3 per group). Data are representative of three independent experiments, and the results are presented as the mean ± SEM. Statistical significance was analyzed usi ng two-way ANOVA (**P* < 0.05, ***P* < 0.01, ****P* < 0.001).

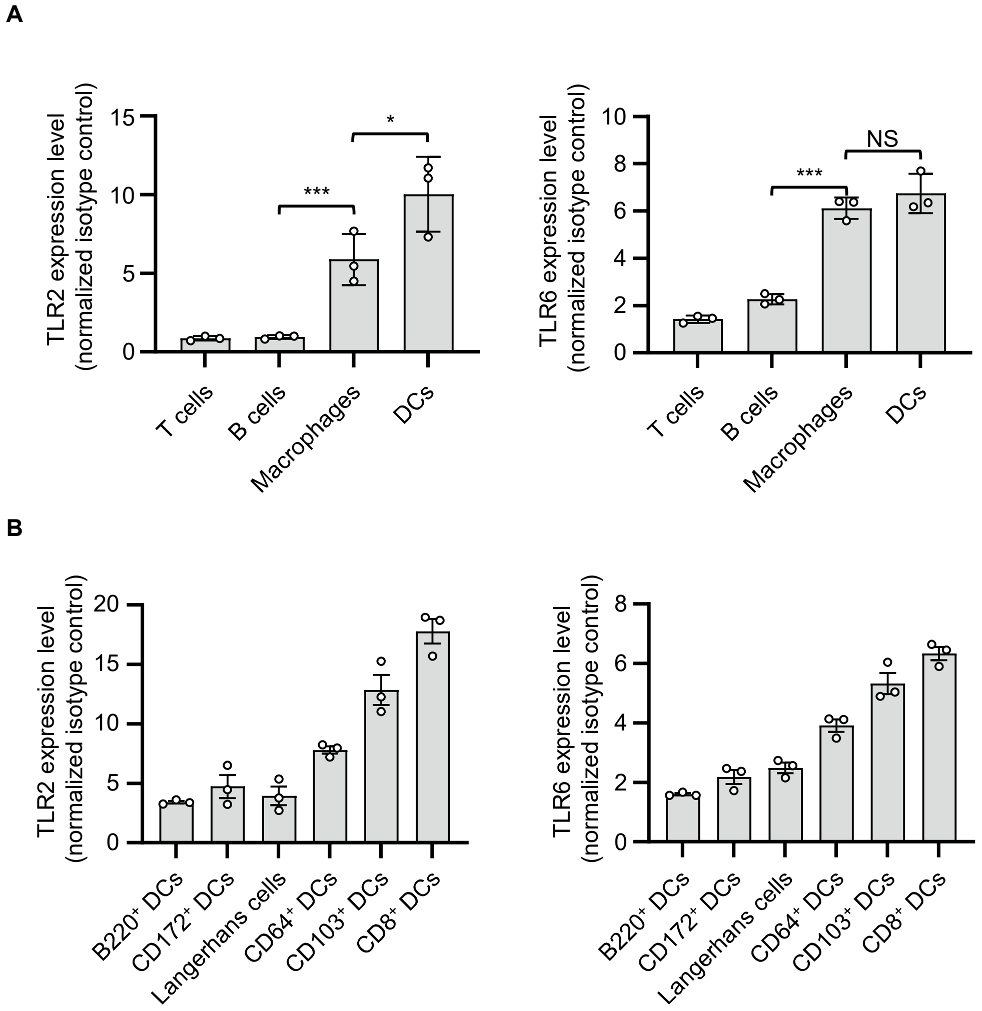

Fig S4. Comparison of TLR2 and TLR6 expression levels by DC subtype. Immune cells were isolated from lymph nodes extracted from naïve mice. TLR2 and TLR6 expression for each isolated (**A**) Immune cells and (**B**) DC subtype was measured using flow cytometry, and then normalized with isotype controls. Data are representative of three independent experiments, and the results are presented as the mean ± SD. Statistical significance was analyzed using Student's t-test (*P < 0.05, **P < 0.01, ***P < 0.001).

A

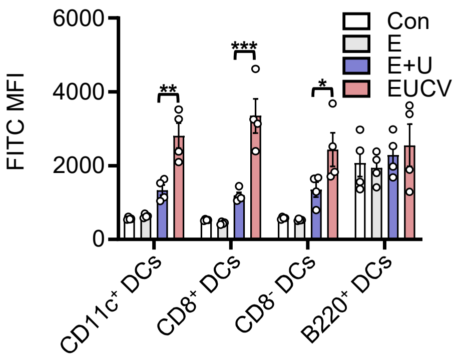

Fig S5. Evaluation of antigen uptake by DC subtypes. (**A**) C57/BL6 mice were subcutaneously injected with 5 nmol of FAM-labeled E, E+U, and EUCV at the right bac k. Antigen uptake was evaluated from DC subtypes residing in the inguinal LN 24 h after subcutaneous injection (*n* = 4 per group). Data are representative of three independent experiments, and the results are presented as the mean ± SD. Statistical significance was analyzed using Student's t-test (*P < 0.05, **P < 0.01, ***P < 0.001).

A

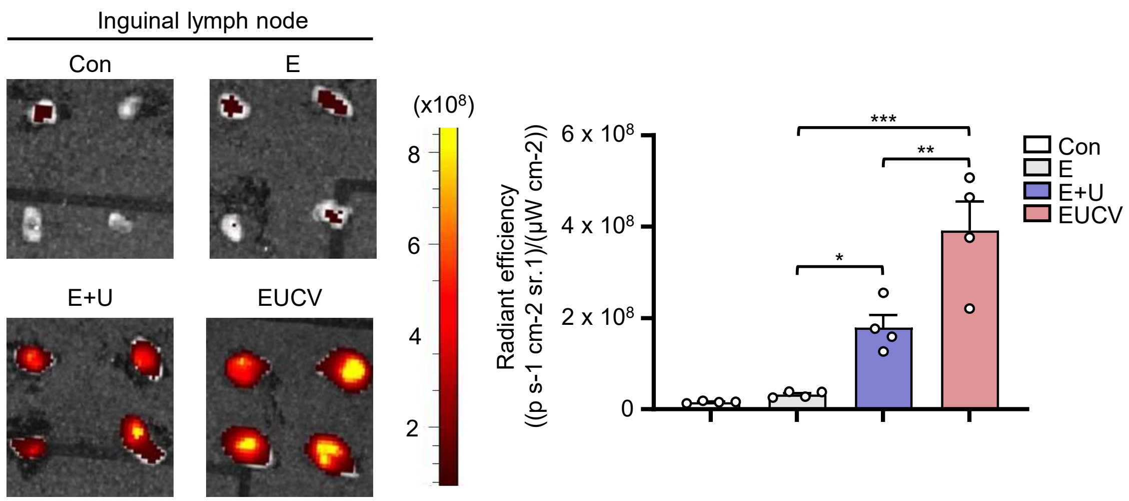

Fig S6. Analysis of vaccine localization patterns in inguinal lymph nodes among different E7 vaccine treatments. (**A**) C57BL/6 mice were subcutaneously injecte d with 5 nmol FAM-labeled E, E+U, and EUCV. After 24 h, lymph nodes isolated from each mouse were imaged, and the signal intensities were quantified using IVIS (*n* = 4 per group). Data are representative of three independent experiments, and the results are presented as mean ± SEM. Statistical significance was analyzed using Stude nt's t-test (*$P < 0.05$, **$P < 0.01$, ***$P < 0.001$).

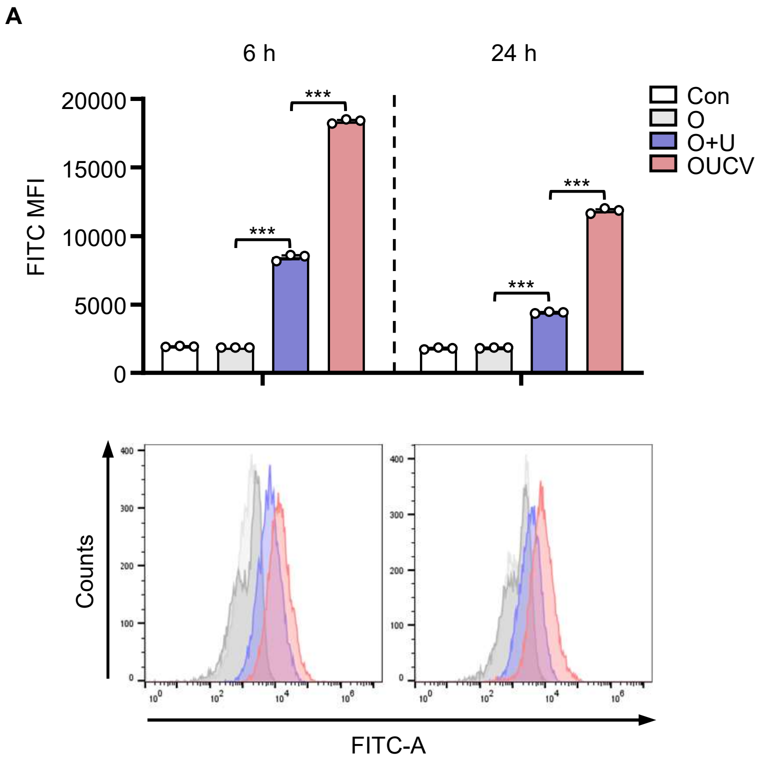

Fig S7. Comparison of OVA uptake among different OVA-based vaccine treatments. (**A**) To investigate the time-dependent uptake of antigens, isolated BMDCs (3× 10^5) were seeded in 24-well plates and incubated with FAM-labeled O, O+U, and OUCV at 37 °C and 5% CO_2 for 6 or 24 h. BMDCs were harvested and stained with 1 μ g/ml APC-conjugated anti-CD11c antibody for 30 min at 4 °C, and antigen uptake was analyzed by flow cytometry. Data are representative of three independent experime nts, and the results are presented as the mean ± SD. Statistical significance was analyzed using Student's t-test (*P < 0.05, **P < 0.01, ***P < 0.001).

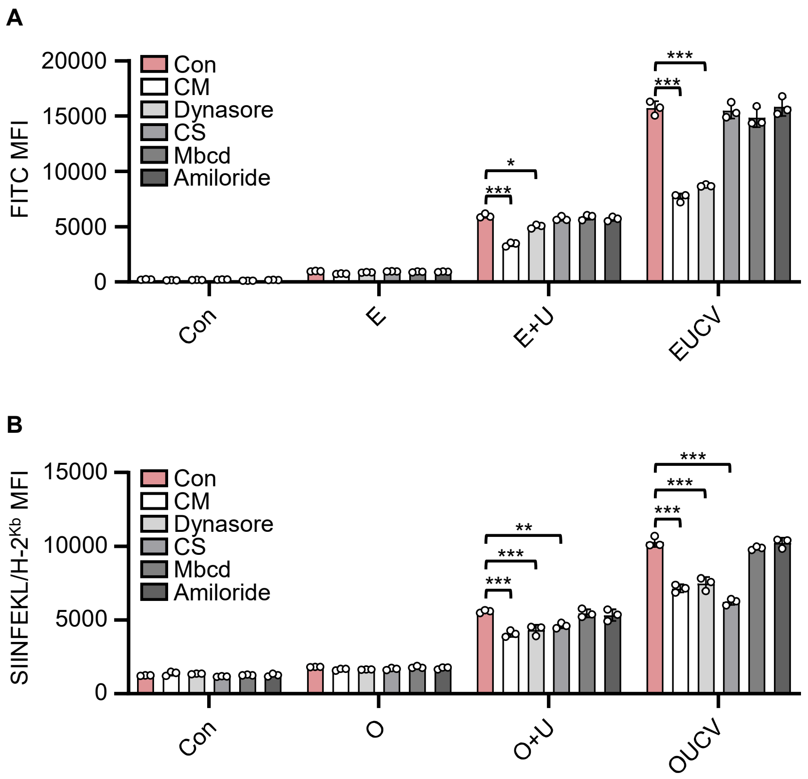

Fig S8. Analysis of the Impact of Inhibitors on Internalization or Antigen Presentation. (**A**) BMDCs isolated from naïve mice were treated with CM (20 μM), Dynaso re (10 μM), CS (1 μM), Mbcd (1 μM) and Amiloride (1 mM) for 30 minutes. To assess antigen uptake, FAM-labeled E, E+U, and EUCV were each treated at 100 nM, and after 24 hours, the FITC+ signal was analyzed by flow cytometry to evaluate the uptake. (**B**) Antigen presentation was evaluated by treating BMDCs with O, O+U, and OUCV after applying each inhibitor, and then measuring the SIINFEKL/H-2Kb signal via flow cytometry 24 hours later. Data are representative of three independent experiments, and the results are presented as the mean ± SD. Statistical significance was analyzed using Student's t-test (*P < 0.05, **P < 0.01, ***P < 0.001).

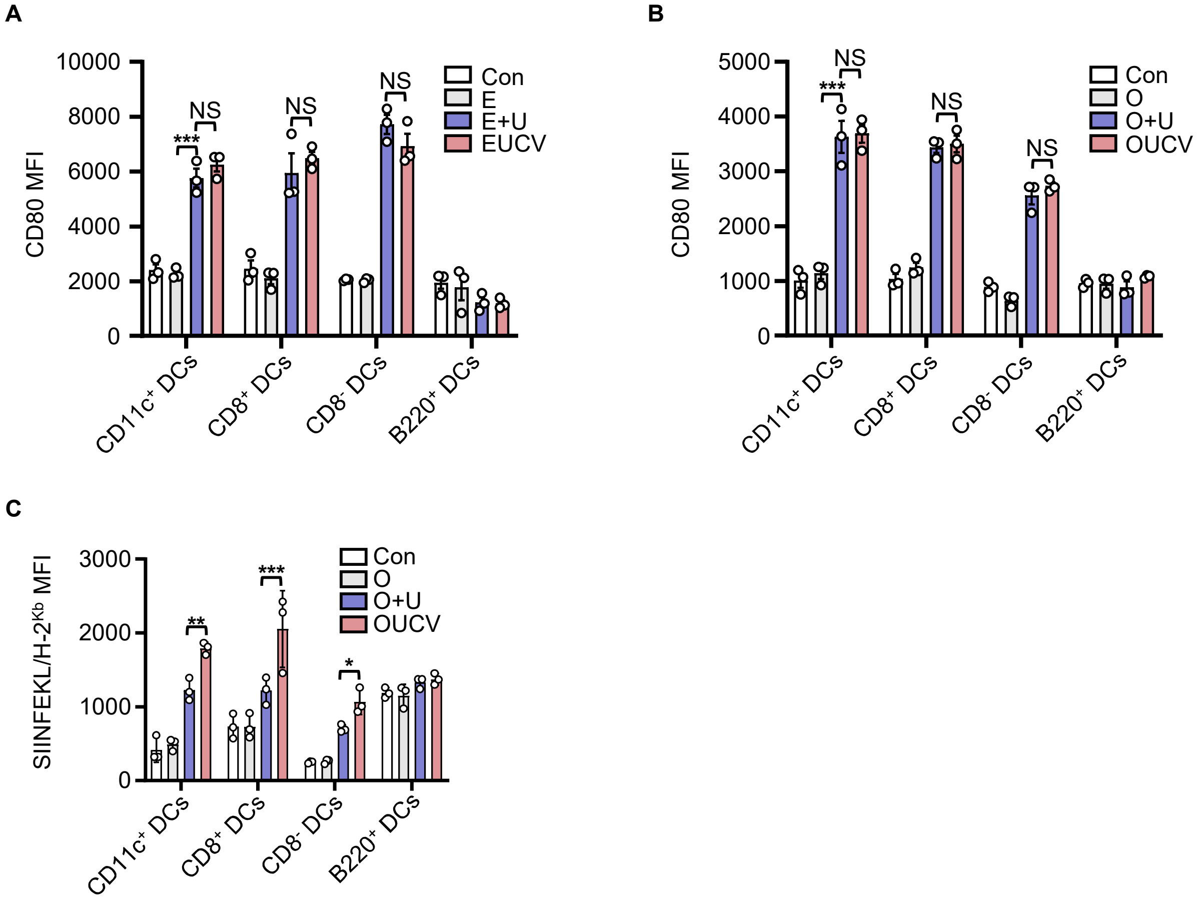

Fig S9. Comparison of costimulatory molecule expression and antigen presentation on DCs injected with different E7- or OVA-based vaccines. (**A**, **B**) Expressi on of the costimulatory molecule CD80 was determined in DC subtypes residing in inguinal lymph nodes 24 h after subcutaneous injection with E7- (**A**) and OVA-based (**B**) vaccines (*n* = 3 per group). (**C**) Flow cytometric analysis of SIINFEKL/H-2Kb expression on LN-residing DC subtypes was performed after subcutaneous injection with 5 nmol of O, O+U, or OUCV, respectively (*n* = 3 per group). Data are representative of three independent experiments, and the results are presented as the mean ± SD. Statistical significance was analyzed using Student's t-test (*P < 0.05, **P < 0.01, ***P < 0.001).

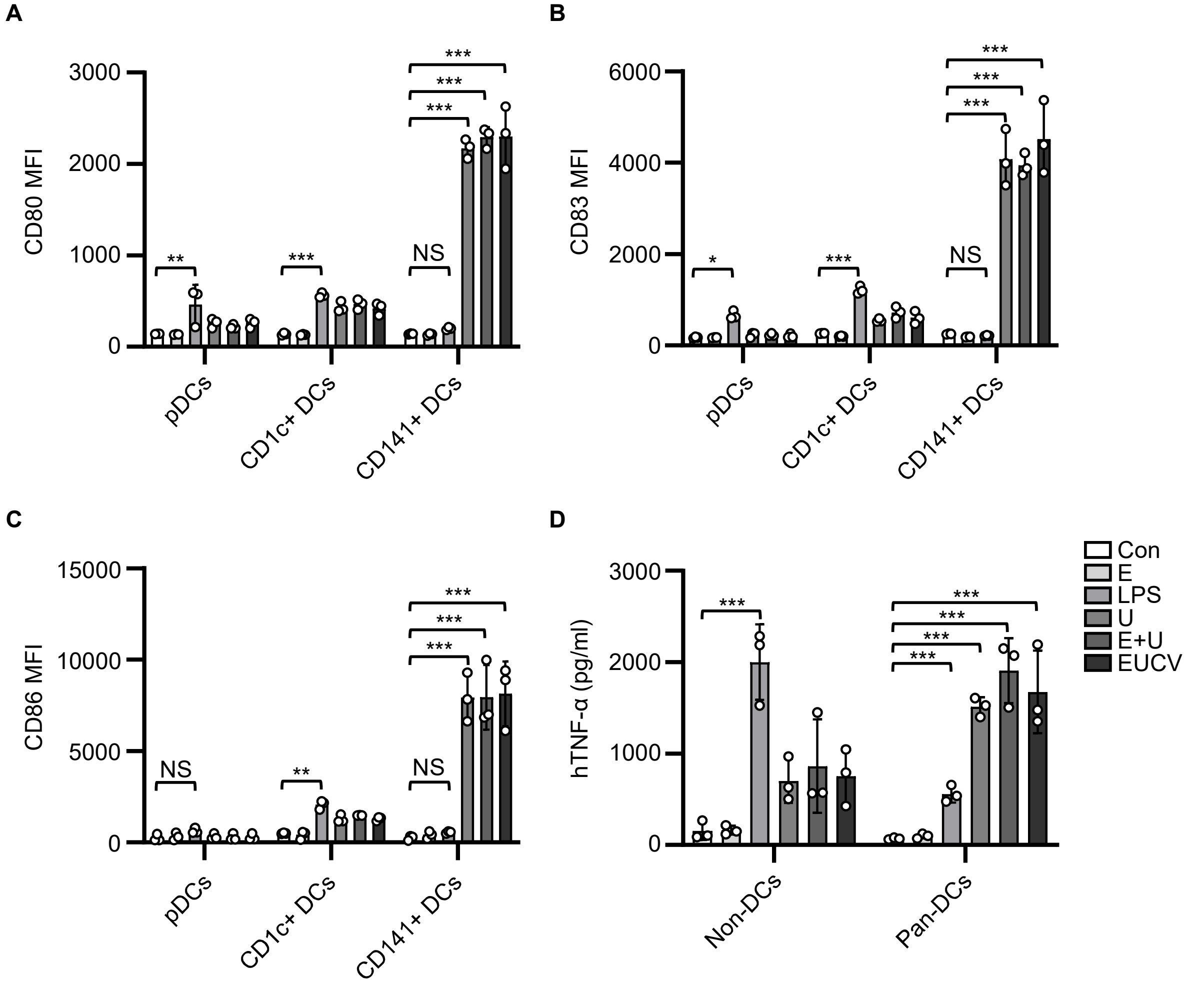

Fig S10. Comparison of the immune stimulation activities of the UCV in Human PBMC isolated DCs. The expression of costimulatory molecules (**A**) CD80, (**B**) CD 83, and (**C**) CD86 was measured using flow cytometry 24 h after treating Pan-DCs isolated from human PBMCs with E, LPS, Poly(I:C), U, E+U, and EUCV, respectively (*n* = 3). (**D**) The inflammatory cytokine hTNF-α levels were quantified using ELISA in Pan-DCs and Non-DCs isolated from human PBMCs, treated with E, LPS, Poly(I:C), U, E+U, and EUCV for 24 hours (*n* = 3). Data are representative of three independent experiments, and the results are presented as the mean ± SD. Statistical significance was analyzed using Student's t-test (*P < 0.05, **P < 0.01, ***P < 0.001).

A

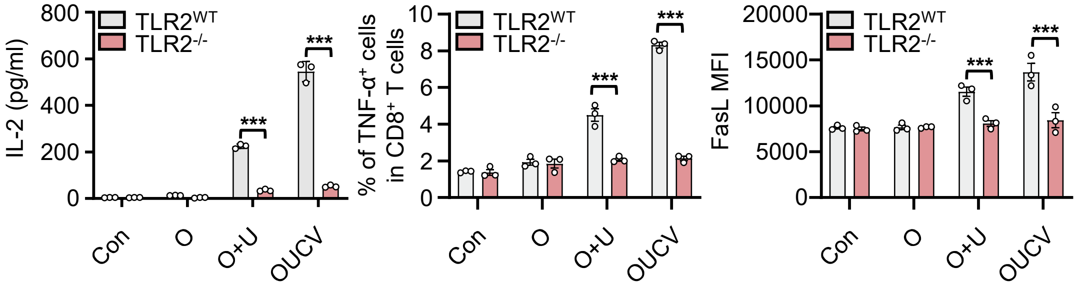

Fig S11. Comparison of $CD8^+$ T-cell stimulation among different OVA-based vaccine treatments. (**A**) BMDCs from TLR2 WT and $TLR2^{-/-}$ mice were incubated for 24 h with O, O+U, or OUCV and cocultured overnight in the presence of B3Z $CD8^+$ T cells. T-cell activation was quantified by IL-2 expression using ELISA, and TNF-α^+ $CD8^+$ T cells population or FasL expression using flow cytometry. Data are representative of three independent experiments, and the results are presented as the mean ± SEM. Statistical significance was analyzed using Student's t-test (****P* < 0.001). NS, not significant.

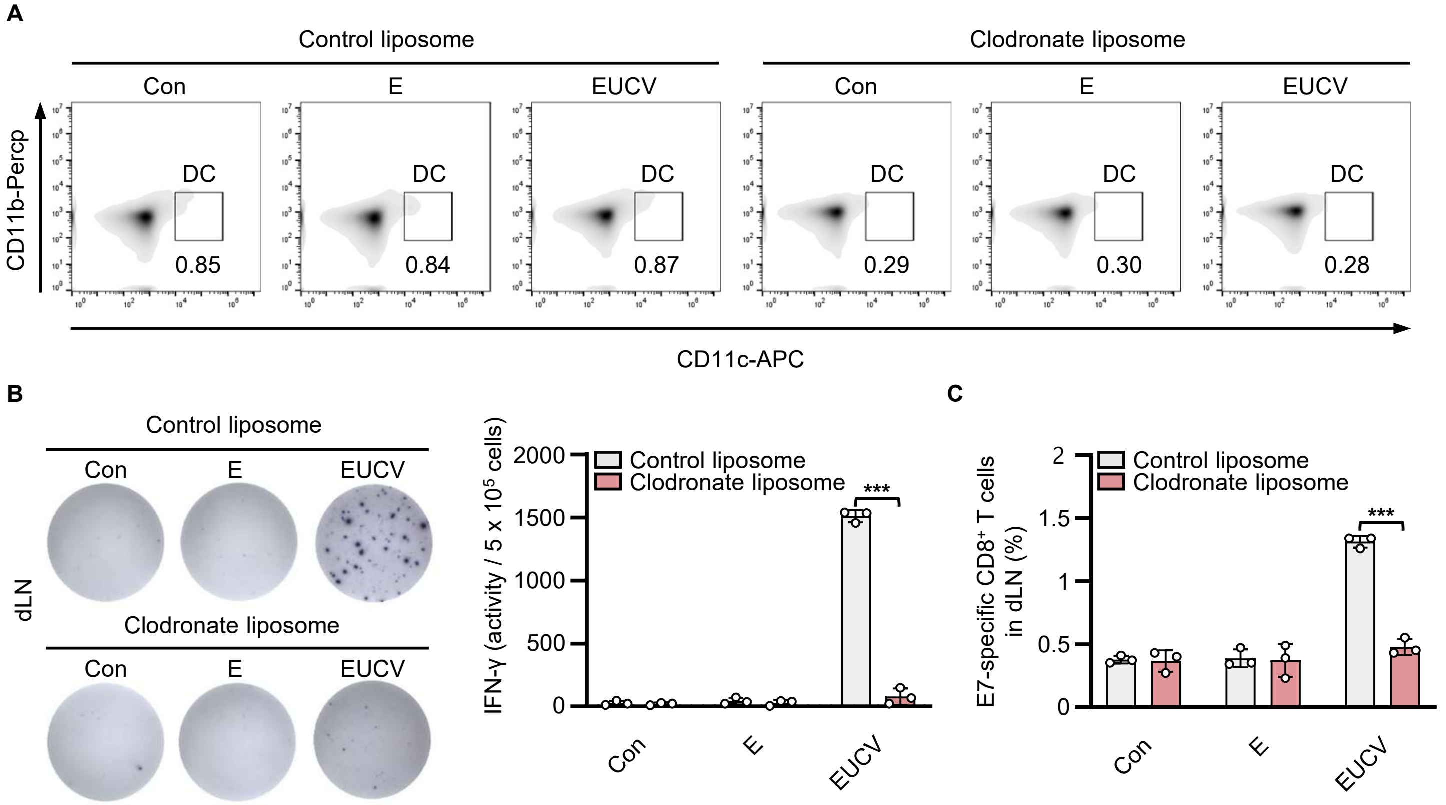

Fig S12. Investigation of DC-dependent Immune Response of EUCV using DC depletion. (**A-C**) C57/BL6 mice were administered 200 μL each of control liposomes and clodronate liposomes via intraperitoneal injection once per week. One day after liposome injection, E and EUCV were each administered at 5 nmol. (**A**) A week after t he last injection, mouse lymph nodes were isolated to confirm DC depletion by flow cytometry (n=3 per group). (**B**) The isolated immune cells (5×10^5 cells) from lymph node were ex vivo stimulated with the E7 epitope (2 μg/ml) for 48 h and analyzed by an ELISpot reader (n = 3 per group). (**C**) Percentages of E7-specific CD8+ T cells in the lymph node were measured using E7 tetramers by flow cytometry (n = 3 per group). Data are representative of three independent experiments, and the results are pr esented as the mean ± SEM. Statistical significance was analyzed using Student's t-test (***$P < 0.001$). NS, not significant.

A

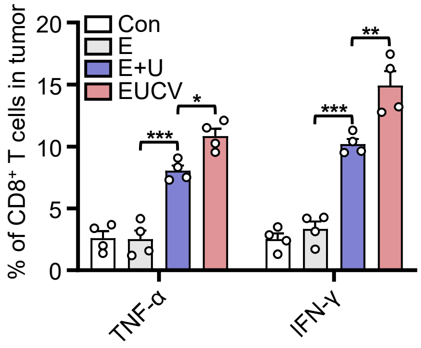

Fig S13. Comparison of CD8+ T-cell stimulation among different E7-based vaccine treatments. (**A**) C57BL/6 mice were subcutaneously inoculated with 1×10^5 TC-1 cells. On day 6 and 13, mice were subcutaneously injected with 5 nmol of E, E+U, and EUCV, and tumors were excised on day 20, and TILs were isolated from tumor tiss ues. The frequencies of TNF-α+ or IFN-γ+ CD8+ T cells were assessed by flow cytometry. Data are representative of three independent experiments, and the results are presented as the mean ± SEM. Statistical significance was analyzed using Student's t-test (*P < 0.05, **P < 0.01, ***P < 0.001). TNF-α, tumor necrosis factor-alpha; IFN-γ, interferon-gamma;

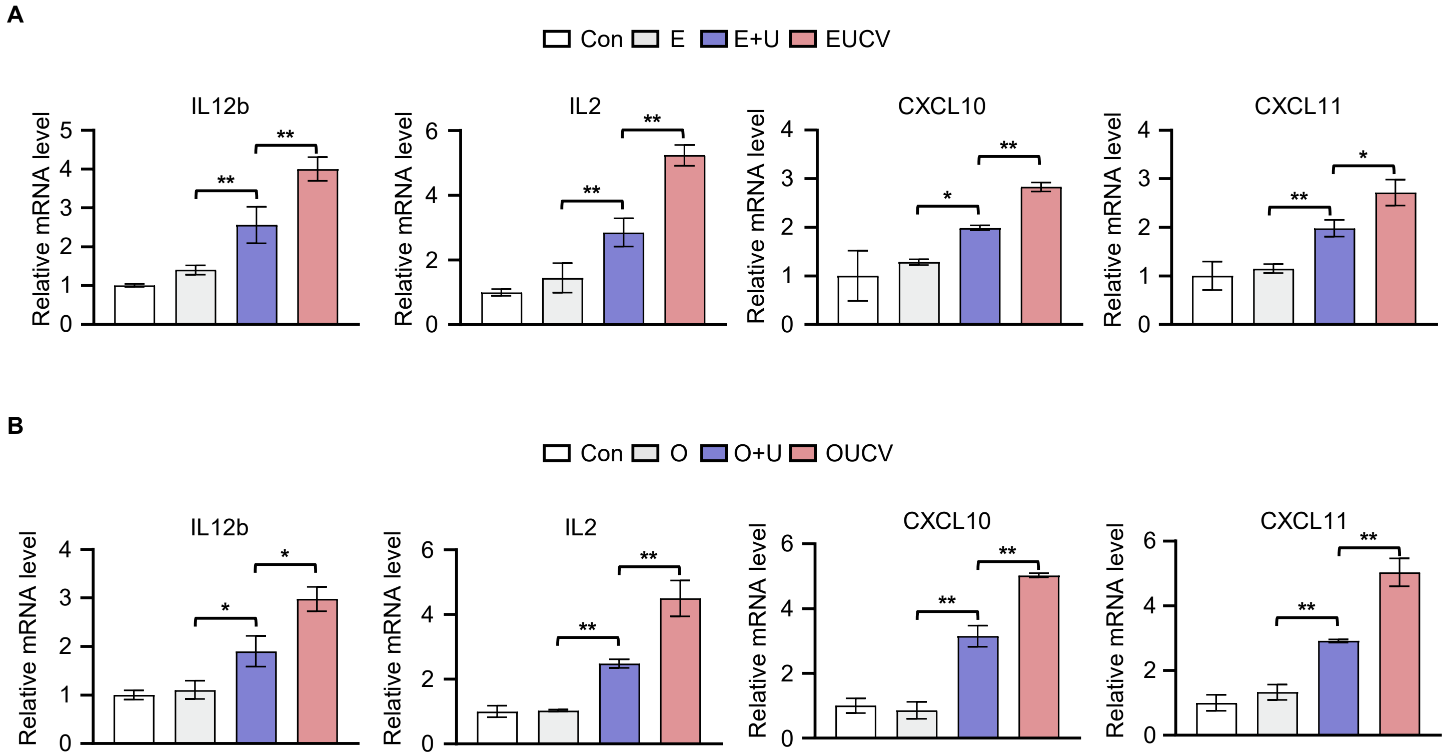

Fig S14. Analysis of CD8⁺ T Cell activation-related gene expression using tumor tissue. (**A-B**) The assessment of gene expression was performed via qPCR using resected TC-1 tumor tissue (**A**) and E.G7-OVA tumor tissue (**B**). Data are representative of three independent experiments, and the results are presented as the mean ± SEM. Statistical significance was analyzed using Student's t-test (*P < 0.05, **P < 0.01, ***P < 0.001).

A

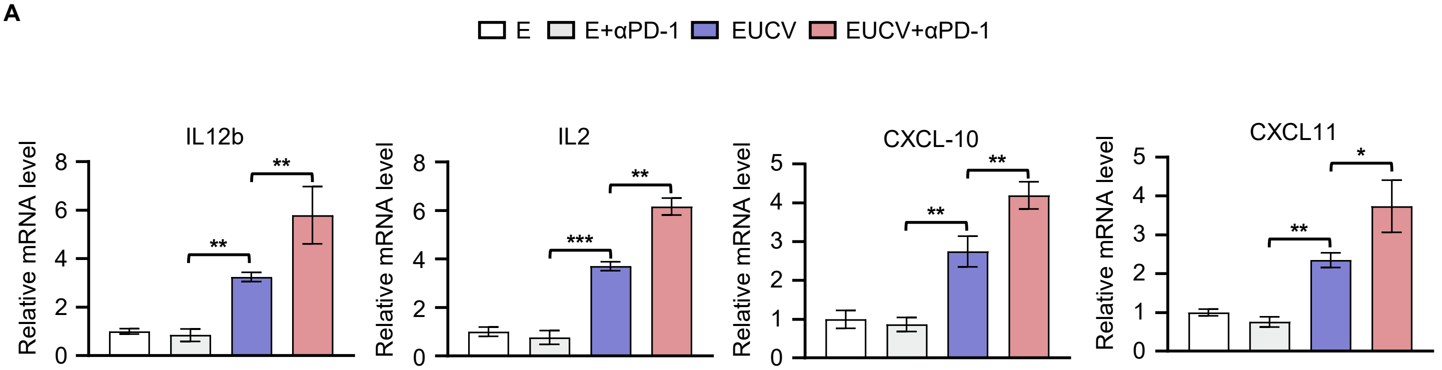

Fig S15. Investigation of CD8⁺ T cell-related gene expression following combined administration of EUCV and PD-1 in a TC-1 tumor model. (**A**) The analysis of gene expression was performed via qPCR using resected tumor tissue. Data are representative of three independent experiments, and the results are presented as the mean ± SEM. Statistical significance was analyzed using Student's t-test (*P < 0.05, **P < 0.01, ***P < 0.001).

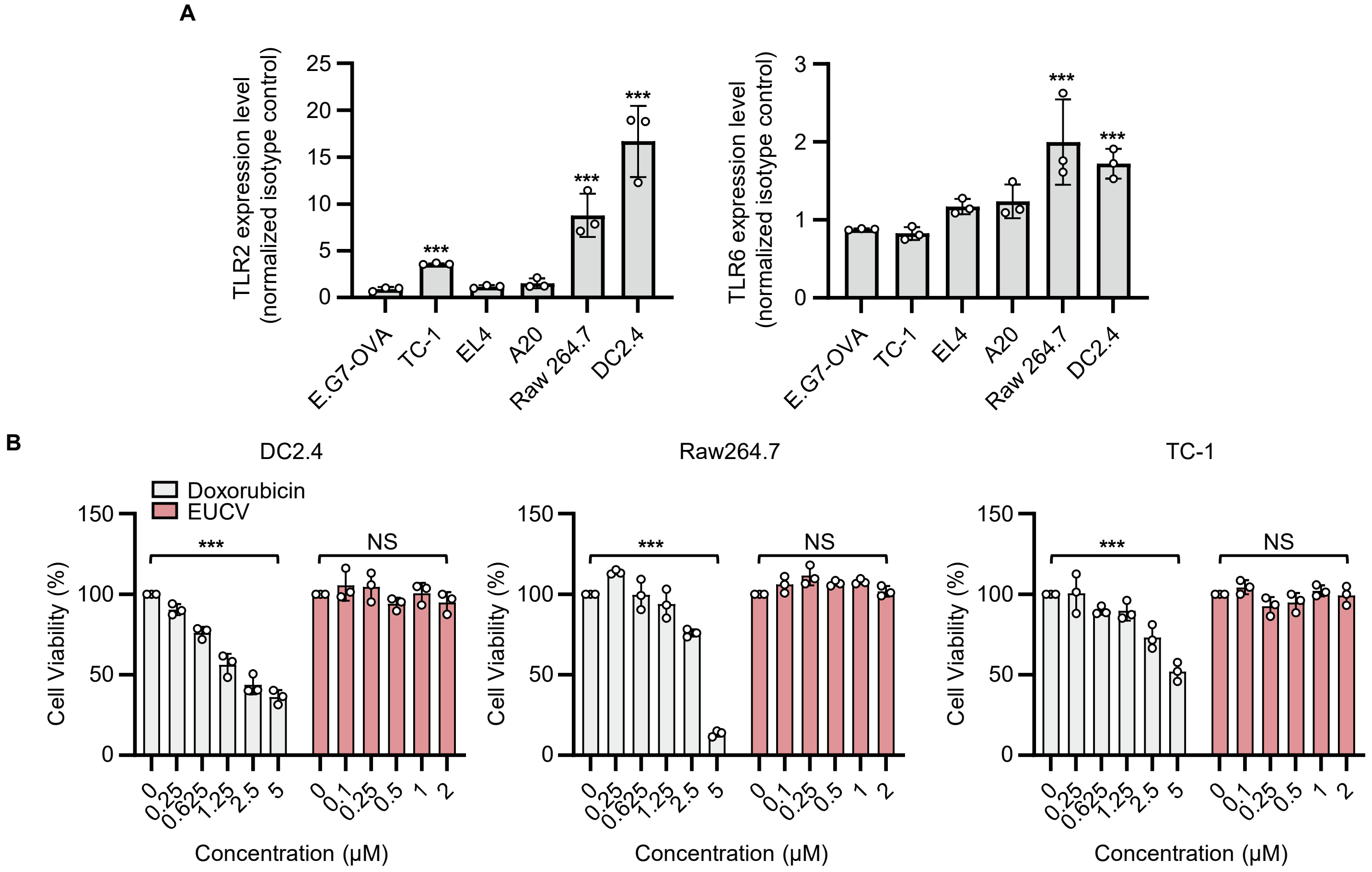

Fig S16. Detection of TLR2 and TLR6 expression levels and verification of EUCV cytotoxicity in cell line. (**A**) TLR2 and TLR6 expression levels were measured by flow cytometry in mouse cancer cell lines E.G7-OVA (lymphoma) and TC-1 (lung cancers), along with immune cell lines EL4 (T cells), A20 (B cells), Raw 264.7 (Macrophages), and DC2.4 (Dendritic cells). The measured signals were normalized using each isotype control. (**B**) DC2.4, Raw264.7, TC-1 cells were seeded onto 96-w ell plates for 24 h before treating with different concentrations of EUCV and Doxorubicin. Cell viability was measured using the Cell Counting Kit-8 (CCK-8) 18 hours after treatment. Data are representative of three independent experiments, and the results are presented as the mean ± SEM. Statistical significance was analyzed using Stud ent's t-test (*P < 0.05, **P < 0.01, ***P < 0.001).

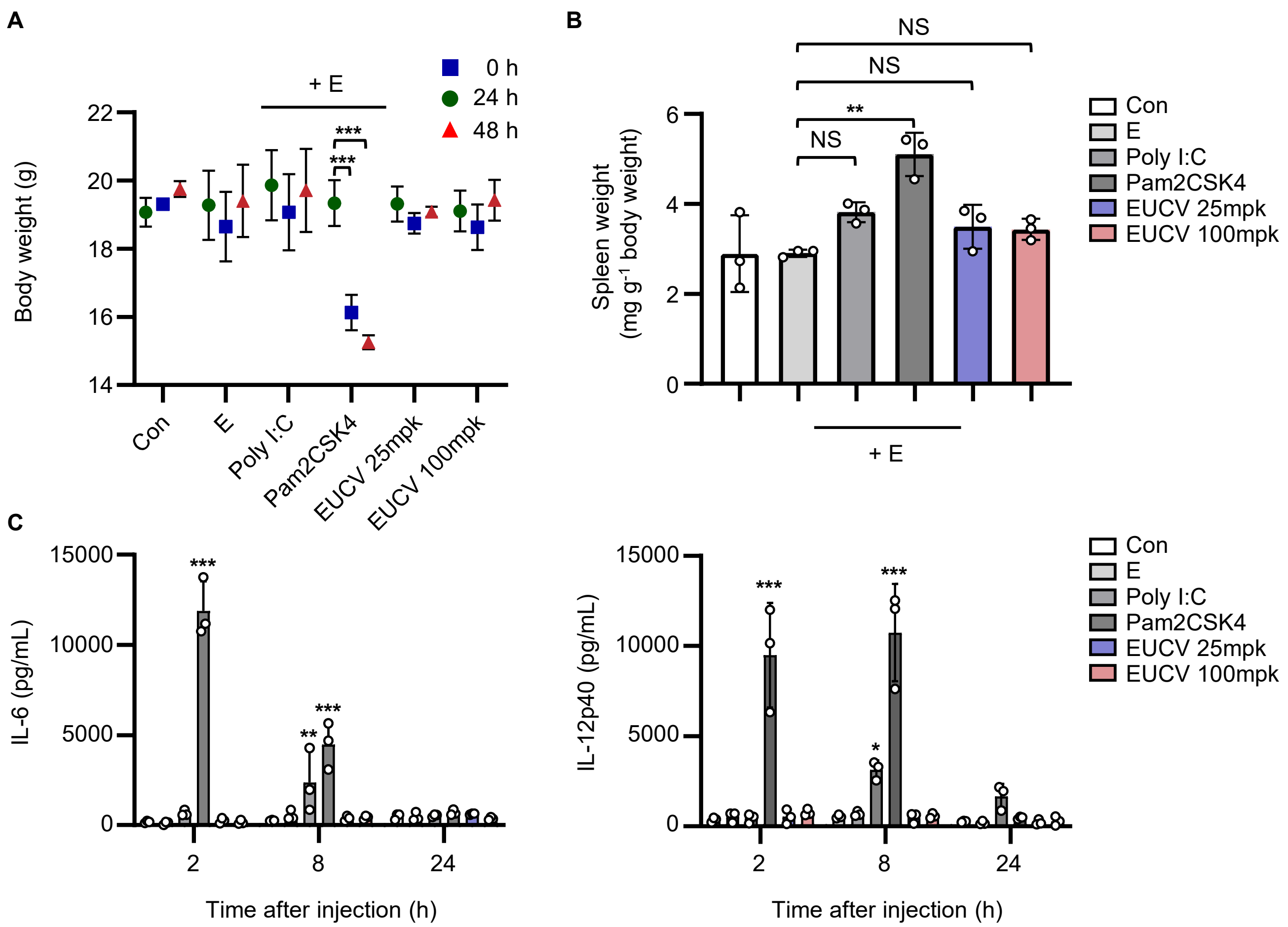

Fig S17. Evaluation of In vivo safety of EUCV. (**A-C**) C57BL/6 mice were administered E, Poly I:C, Pam2CSK4, or EUCV (25 or 100 mpk). (**A**) Body weight was measured every 24 hours, and 48 hours after administration, (**B**) the mice were euthanized to measure spleen weight. (**C**) Proinflammatory cytokines IL-6 and IL-12p40 were measured using ELISA with serum extracted from the mice. Data are representative of three independent experiments, and the results are presented as the mean ± SEM. Statistical significance was analyzed using Student's t-test (*P < 0.05, **P < 0.01, ***P < 0.001).

A

Fig S1C

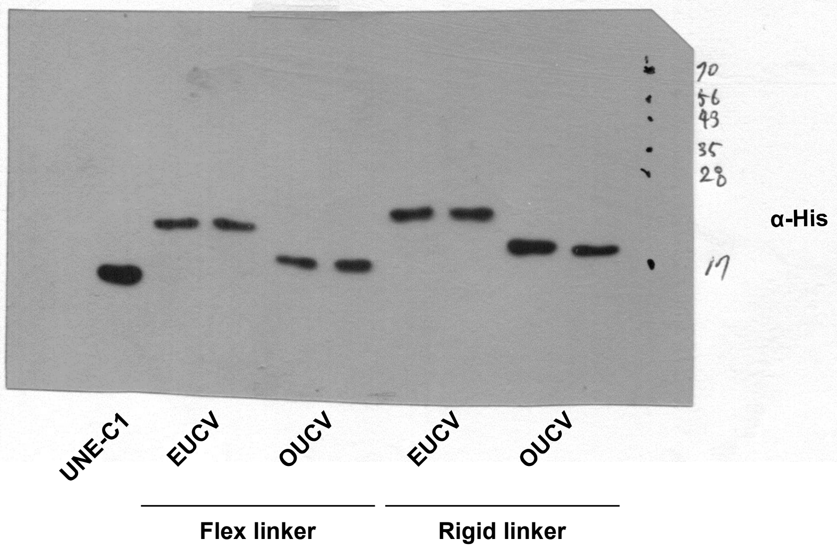

Fig S18. Raw data for Western blots included in the figures. (**A**) Uncropped image of western blot displayed in Fig S1C.

Table S1. The amino acid sequences of SLP antigens and the UCVs used in this study.

Abbreviation	Sequence
E (HPV16 E7 oncoprotein, 43~62 aa)	GQAEPDRAHYNIVTFCCKCD
O (Ovalbumin, 247~264 aa + AAAAK)	DEVSGLEQLESIINFEKLAAAAAK
EUCV (HPV 16 E7 43-62 aa conjugated UNE-C1)	GQAEPDRAHYNIVTFCCKCDGGGGSGGGGSGGGGSARQNHL FEQYREKRPEAAQLLEDVQAALKPFSVKLNETTDPDKKQMLER IQHAVQLATEPLEKAVQSRLTGEEVNSCVEVLLEEAKDLLSDWL DSTLGCDVTDNSIFSKLPKFWEGDFHRDME
OUCV (Ovalbumin 247-264 aa +AAAAK conjugated UNE-C1)	DEVSGLEQLESIINFEKLAAAAAKGGGGSGGGGSGGGGSARQ NHLFEQYREKRPEAAQLLEDVQAALKPFSVKLNETTDPDKKQM LERIQHAVQLATEPLEKAVQSRLTGEEVNSCVEVLLEEAKDLLS DWLDSTLGCDVTDNSIFSKLPKFWEGDFHRDME

Table S2. The DNA sequences of the UCVs used in this study.

Plasmid	Sequence
pET28a-E(F)UCV → The sequence of 6xHis-E7$_{43-62}$-(GGGGS)$_3$-UNE-C1-6xHis as shown in left was inserted into pET28a back bone using NdeI and XhoI. 20729.93	TATACCATGGGCAGCAGCCATCATCATCATCATCACAGCAGCGGCCTGGTGCCGCGCGGCAGC<br>CATATG<br>GGTCAAGCTGAACCAGATCGTGCTCACTATAACATTGTTACTTTCTGTTGTAAATGTGAT<br>GGTGGTGGTGGTTCTGGTGGTGGTGGTTCTGGTGGTGGTGGTTCT<br>GCCCGGCAGAACCACCTGTTCGAGCAGTATCGGGAGAAGAGGCCTGAAGCGGCACAGCTCTTGGA<br>GGATGTTCAGGCCGCCCTGAAGCCATTTTCAGTAAAATTAAATGAGACCACGGATCCCGATAAAAAG<br>CAGATGCTCGAACGGATTCAGCACGCAGTGCAGCTTGCCACAGAGCCACTTGAGAAAGCTGTGCAG<br>TCCAGACTCACGGGAGAGGAAGTCAACAGCTGTGTGGAGGTGTTGCTGGAAGAAGCCAAGGATTTG<br>CTCTCTGACTGGCTGGATTCTACACTTGGCTGTGATGTCACTGACAATTCCATCTTCTCCAAGCTGCC<br>CAAGTTCTGGGAGGGGGACTTCCACAGAGACATGGAA<br>CTCGAGCACCACCACCACCACCACTGA
pET28a-E(R)UCV → The sequence of 6xHis-E7$_{43-62}$-(EAAAK)$_3$-UNE-C1-6xHis as shown in left was inserted into pET28a back bone using NdeI and XhoI.21.195	TATACCATGGGCAGCAGCCATCATCATCATCATCACAGCAGCGGCCTGGTGCCGCGCGGCAGC<br>CATATG<br>GGTCAAGCTGAACCAGATCGTGCTCACTATAACATTGTTACTTTCTGTTGTAAATGTGAT<br>GAAGCTGCTGCTAAAGAAGCTGCTGCTAAAGAAGCTGCTGCTAAA<br>GCCCGGCAGAACCACCTGTTCGAGCAGTATCGGGAGAAGAGGCCTGAAGCGGCACAGCTCTTGGA<br>GGATGTTCAGGCCGCCCTGAAGCCATTTTCAGTAAAATTAAATGAGACCACGGATCCCGATAAAAAG<br>CAGATGCTCGAACGGATTCAGCACGCAGTGCAGCTTGCCACAGAGCCACTTGAGAAAGCTGTGCAG<br>TCCAGACTCACGGGAGAGGAAGTCAACAGCTGTGTGGAGGTGTTGCTGGAAGAAGCCAAGGATTTG<br>CTCTCTGACTGGCTGGATTCTACACTTGGCTGTGATGTCACTGACAATTCCATCTTCTCCAAGCTGCC<br>CAAGTTCTGGGAGGGGGACTTCCACAGAGACATGGAA<br>CTCGAGCACCACCACCACCACCACTGA
pET28a-O(F)UCV → The sequence of 6xHis-OVA$_{247-264(AAAAK)}$-(GGGGS)$_3$-UNE-C1-6xHis as shown in left was inserted into pET28a back bone using NdeI and XhoI. 21006.25	TATACCATGGGCAGCAGCCATCATCATCATCATCACAGCAGCGGCCTGGTGCCGCGCGGCAGC<br>CATATG<br>GATGAAGTTTCTGGTTTAGAACAATTAGAATCTATTATTAACTTCGAAAAATTAGCTGCTGCTGCTGCT<br>AAA<br>GGTGGTGGTGGTTCTGGTGGTGGTGGTTCTGGTGGTGGTGGTTCT<br>GCCCGGCAGAACCACCTGTTCGAGCAGTATCGGGAGAAGAGGCCTGAAGCGGCACAGCTCTTGGA<br>GGATGTTCAGGCCGCCCTGAAGCCATTTTCAGTAAAATTAAATGAGACCACGGATCCCGATAAAAAG<br>CAGATGCTCGAACGGATTCAGCACGCAGTGCAGCTTGCCACAGAGCCACTTGAGAAAGCTGTGCAG<br>TCCAGACTCACGGGAGAGGAAGTCAACAGCTGTGTGGAGGTGTTGCTGGAAGAAGCCAAGGATTTG<br>CTCTCTGACTGGCTGGATTCTACACTTGGCTGTGATGTCACTGACAATTCCATCTTCTCCAAGCTGCC<br>CAAGTTCTGGGAGGGGGACTTCCACAGAGACATGGAA<br>CTCGAGCACCACCACCACCACCACTGA
pET28a-O(R)UCV → The sequence of 6xHis-OVA$_{247-264(AAAAK)}$-(EAAAK)$_3$-UNE-C1-6xHis as shown in left was inserted into pET28a back bone using NdeI and XhoI. 21471.97	TATACCATGGGCAGCAGCCATCATCATCATCATCACAGCAGCGGCCTGGTGCCGCGCGGCAGC<br>CATATG<br>GATGAAGTTTCTGGTTTAGAACAATTAGAATCTATTATTAACTTCGAAAAATTAGCTGCTGCTGCTGCT<br>AAA<br>GAAGCTGCTGCTAAAGAAGCTGCTGCTAAAGAAGCTGCTGCTAAA<br>GCCCGGCAGAACCACCTGTTCGAGCAGTATCGGGAGAAGAGGCCTGAAGCGGCACAGCTCTTGGA<br>GGATGTTCAGGCCGCCCTGAAGCCATTTTCAGTAAAATTAAATGAGACCACGGATCCCGATAAAAAG<br>CAGATGCTCGAACGGATTCAGCACGCAGTGCAGCTTGCCACAGAGCCACTTGAGAAAGCTGTGCAG<br>TCCAGACTCACGGGAGAGGAAGTCAACAGCTGTGTGGAGGTGTTGCTGGAAGAAGCCAAGGATTTG<br>CTCTCTGACTGGCTGGATTCTACACTTGGCTGTGATGTCACTGACAATTCCATCTTCTCCAAGCTGCC<br>CAAGTTCTGGGAGGGGGACTTCCACAGAGACATGGAA<br>CTCGAGCACCACCACCACCACCACTGA

Table S3. The amino sequences of SLPs and epitope peptides used in this study.

Type	HPV 16 Protein	Start	End	Peptide sequence
SLP	E6	23	42	CTELQTTIHDIILECVYCKQ
	E6	43	62	QLLRREVYDFAFRDLCIVYR
	E6	123	143	QRHLDKKQRFHNIRGRWTGRC
	E7	5	25	TPTLHEYMLDLQPETTDLYCY
	E7	43	62	GQAEPDRAHYNIVTFCCKCD
	E7	76	95	IRTLEDLLMGTLGIVCPICS
Epitope peptide	E6	29	37	TIHDIILEC
	E6	49	57	VYDFAFRDL
	E6	129	138	KQRFHNIRGR
	E7	11	19	YMLDLQPETT
	E7	49	57	RAHYNIVTF
	E7	82	90	LLMGTLGIV

Table S4. The DNA sequences of PCR primers used in this study.

Target	Sequence (5' → 3')
Mus IFN-γ	F: ACTGGCAAAAGGATGGTGAC<br>R: TGAGCTCATTGAATGCTTGG
Mus Perforin	F: GATGTGAACCCTAGGCCAGA<br>R: GGTTTTTGTACCAGGCGAGA
Mus Granzyme B	F: GACAACACTCTTGACGCTGG<br>R: TGATCTCCCCTGCCTTTGTCC
Mus CXCL-9	F: CTTGAGCCTAGTCGTGATAAC<br>R: CCAGCTTGGTGAGGTCTATC
Mus Il12b	F: CCAGAGACATGGAGTCATAG<br>R: AGATGTGAGTGGCTCAGAGT
Mus IL-2	F: GCGGCATGTTCTGGATTTGACTC<br>R: CCACCACAGTTGCTGACTCATC
Mus Granulysin	F: GATAAGCCCACCCAGAGAAGTG<br>R: CGTGACCTCCCCGTCCTA
Mus FasL	F: CACAAATCTGTGGCTACCG<br>R: GCCCATATCTGTCCAGTAG
Mus CXCL10	F: CCACGTGTTGAGATCATTGC<br>R: AGTAGCAGCTGATGTGACG
Mus CD69	F: AGGCTTGTACGAGAAGTTGGA<br>R: AGTTCACCAGAATATCGCTTCAG
Mus tbx21	F: CAACCAGCACCAGACAGAGA<br>R: CCACATCCACAAACATCCTG
Mus GAPDH	F: TCAATGAAGGGGTCGTTGAT<br>R: CGTCCCGTAGACAAAATGGT
